# A new nurse frog of the *Allobates tapajo*s species complex (Anura: Aromobatidae) from the upper Madeira River, Brazilian Amazonia

**DOI:** 10.7717/peerj.13751

**Published:** 2022-08-03

**Authors:** Miquéias Ferrão, James Hanken, Albertina P. Lima

**Affiliations:** 1 Museum of Comparative Zoology, Harvard University, Cambridge, MA, United States of America; 2 Coordenação de Pesquisas em Biodiversidade, Instituto Nacional de Pesquisas da Amazônia, Manaus, Amazonas, Brazil

**Keywords:** Advertisement call, Breeding behavior, Morphology, Phylogeny, Biodiversity, Riparian forest, Tadpole, Taxonomy

## Abstract

Cryptic diversity is extremely common in widespread Amazonian anurans, but especially in nurse frogs of the genus *Allobates*. There is an urgent need to formally describe the many distinct but unnamed species, both to enable studies of their basic biology but especially to facilitate conservation of threatened environments in which many are found. Here, we describe through integrative taxonomy a new species of the *Allobates tapajos* species complex from the upper Madeira River, southwestern Amazonia. Species delimitation analyses based on molecular data are congruent and delimit five candidate species in addition to *A. tapajos* sensu stricto. The new species is recovered as sister to *A. tapajos* clade F, a candidate species from Teles-Pires River, southeastern Amazonia. The new species differs from nominal congeners in adult and larval morphology and in male advertisement call. Egg deposition sites differ between east and west banks of the upper Madeira River, but there is no evidence of corresponding morphologic or bioacoustic differentiation. The new species appears to be restricted to riparian forests; its known geographic range falls entirely within the influence zone of reservoirs of two large dams, which underscores the urgent need of a conservation assessment through long-term monitoring. This region harbors the richest assemblage of *Allobates* reported for Brazilian Amazonia, with six nominal species and four additional candidate species awaiting formal description.

## Introduction

Many species of Amazonian frogs once thought to be geographically widespread are now known to contain numerous additional species, each with a narrow distribution (Lima, Ferrão & Silva, 2020; Vacher et al., 2020; Fouquet et al., 2021a; Fouquet et al., 2021b). This phenomenon is especially evident in nurse frogs of the genus *Allobates* (Zimmermann & Zimmermann, 1988). Although previously hidden diversity has been revealed in some species (*e.g.*, Grant et al., 2006; Grant et al., 2017; Lima, Ferrão & Silva, 2020), a recent widescale DNA barcoding study reported that approximately 50% of nominal species in the entire genus represent species complexes (Vacher et al., 2020). This finding and the high number of recently described species highlight the need for extensive taxonomic studies that fully reveal and organize the astonishing diversity of *Allobates*.

*Allobates* is characterized by a high degree of phenotypic conservatism among species despite their deep genotypic and acoustic differentiation (Kaefer et al., 2013). This conservatism originally misled taxonomists to regard subtle phenotypic differentiation among species as intraspecific variation, which for decades hampered the discovery and description of new taxa. This scenario has been rejected mostly in the last decade following the integration of multiple and independent lines of evidence (*e.g.*, morphology of adults, larvae and eggs, vocalization, and molecular data) in taxonomic studies. Indeed, 18 of the 19 species of *Allobates* named since 2010 have been described through integrative taxonomy (*e.g.*, Moraes, Pavan & Lima, 2019; Souza et al., 2020; Silva et al., 2022), while only one description was based solely on morphological characters (Anganoy-Criollo, 2012). Although integrative taxonomy may be useful in contemporary treatments of any taxon (Padial et al., 2010), it is an essential approach for deriving solid and reliable diagnoses of new species of *Allobates*, especially those that belong to species complexes (Lima, Ferrão & Silva, 2020).

*Allobates tapajos* (Lima, Simões & Kaefer, 2015) is a good model to study hidden diversity through integrative taxonomy. It has been considered a species complex in recent molecular studies, in which species delimitation methods recognize from three to five candidate species (Réjaud et al., 2020; Silva et al., 2022). While *A. tapajos* sensu stricto occurs in eastern Brazilian Amazonia, one candidate species inhabits northeastern Amazonia in the Guiana Shield, another one occurs in southeastern Amazonia, and three others occupy central and southwestern Brazilian Amazonia (Vacher et al., 2020; Silva et al., 2022). One of the latter candidate species was known until recently from only a single specimen, HJ 285, collected in the upper Madeira River (Vacher et al., 2020). In this study, we integrate external morphology of additional adults and larvae, advertisement call, breeding behavior and molecular data to describe this candidate as a new species.

## Materials and Methods

### Sampling

Specimens were collected between 2004 and 2019 in eight sampling modules in the upper Madeira Basin, municipality of Porto Velho, state of Rondônia, Brazil. Four modules are located along the west bank of the Madeira River (*Abunã Esquerdo* (−9.516000, −65.324900; ∼105 m above sea level; masl), *Jirau Esquerdo* (−9.317073, −64.743354; ∼85 masl), *Pedras* (−9.167074, −64.629109; ∼73 masl) and *Búfalos* (−9.133275, −64.497260; ∼71 masl)) and three along the east bank (*Abunã Direito* (−9.618408, −65.391275; ∼103 masl), *Jirau Direito* (−9.361940, −64.691940; ∼95 masl) and *Morrinhos* (−9.076111, −64.246111; ∼79 masl)). The eighth module is located along the Jací-Paraná River (Jací (−9.4125 −64.4425; ∼83 masl)), a tributary of the west bank of the Madeira River. Seventy-five adults were euthanized with a topical solution of 2% aqueous benzocaine, fixed in 10% neutral-buffered formalin (NBF) and stored in 70% ethanol.

Fifty-one tadpoles were obtained by A. P. Lima in January 2011 from clutches deposited in male territories in the *Morrinhos* (*n* = 1) and *Jirau Direito* (*n* = 2) sampling modules. Tadpoles were reared for morphological description in 40 × 60 × 10 cm plastic containers with water until they reached Gosner stages 34–40 the following month. They were killed with aqueous benzocaine solution and preserved in 5% NBF. Adults and tadpoles are deposited in the Herpetology Collection of the Instituto Nacional de Pesquisas da Amazônia (INPAH), Manaus, Brazil. Protocols of collection and animal care follow the Brazilian Federal Council for Biology resolution number 148/2012 (Conselho Federal de Biologia–CFBio, 2012). Specimens were collected under collection permit numbers 1337-1 and 02001.000508/2008-99 issued by the Instituto Brasileiro do Meio Ambiente e dos Recursos Naturais Renováveis –IBAMA (Ministry of Environment, Government of Brazil).

Advertisement calls of 16 males were recorded on the west bank (*Abunã Esquerdo* (*n* = 8), *Búfalos* (*n* = 5), *Jirau Esquerdo* (*n* = 2) and *Pedras* (*n* = 1)) and 14 on the east bank (*Abunã Direito* (*n* = 3), *Jirau Direito* (*n* = 5) and *Morrinhos* (*n* = 6)) with an AKG 568 EB microphone connected to a Sony WM-D6C tape recorder. The microphone was positioned approximately 1 m from each focal male. Raven 1.5 (Bioacoustics Research Program, 2015) was used to digitize call recordings from tapes and store them in WAV format with sampling rate of 44,100 Hz and 16 bits. Air temperature at the time of recording was measured with a digital thermometer and ranged from 23.8 to 28.6 °C. Recorded males ranged from 15.3 to 16.9 mm in snout–vent length. Call recordings are housed in the public repository Fonoteca Neotropical Jacques Vielliard (University of Campinas, Campinas, Brazil; https://www2.ib.unicamp.br/fnjv/) under accession numbers FNJV 50564–50593.

### Sequencing and phylogenetic analysis

Total genomic DNA was extracted from tissue samples of 14 specimens of the new species—six from the east bank of the upper Madeira River and eight from the west bank. We also extracted genomic DNA of two specimens from the *Jací* sampling module that resemble *Allobates tapajos* sensu lato. DNA extractions were obtained using the commercial kit Wizard (Promega Corp., Madison, WI, USA) following the manufacturer’s instructions. Fragments of the 16S rRNA mitochondrial gene were amplified by polymerase chain reaction (PCR) using the universal primers 16sar (CGCCTGTTTATCAAAAACAT) and 16sbr (CCGGTCTGAACTCAGATCACGT; Palumbi, 1996). The PCR and DNA sequencing protocols followed Maia, Lima & Kaefer (2017). Amplicons were visually checked and manually edited in Geneious 5.3.4 (Kearse et al., 2012). Sequences, ranging in length from 451 to 552 base pairs (bp), are deposited at GenBank under accession numbers MZ667618 –MZ667633.

Additional mitochondrial gene sequences were downloaded from GenBank to infer the phylogenetic relationship of the new species: 95 sequences of 16S and 64 sequences of 12S ribosomal RNA (16S, 12S), 50 sequences of cytochrome c oxidase I (COI), 66 sequences of cytochrome b (CYTB) and 41 sequences of NADH dehydrogenase 1 (ND1). Sequences of *Ameerega* Bauer (1986), *Anomaloglossus* Grant et al. (2006), *Aromobates* Myers, Paolillo-O. and Daly (1991), *Colostethus* Cope (1866), *Dendrobates* Wagler (1830), *Leucostethus* Grant et al. (2017), *Mannophryne* La Marca (1992), *Phyllobates* Bibron (1840), *Rheobates* Grant et al. (2006) and *Silverstoneia* Grant et al. (2006) were used to root the tree. Gene sampling followed Réjaud et al. (2020); taxon sampling followed Melo-Sampaio, Oliveira & Prates (2018), Réjaud et al. (2020) and Vacher et al. (2020). Table S1 lists voucher specimens and GenBank accession numbers.

Sequences were aligned independently for each gene through the MAFFT online server (https://mafft.cbrc.jp/) using the E-INS-i and G–INS–i strategies with default parameters for RNAs and protein-coding genes, respectively (Katoh & Standley, 2013). Alignments were concatenated in Mesquite 3.6 (Maddison & Maddison, 2019); the final concatenated matrix was composed of 6,235 bp and 108 terminals. The dataset was divided into 11 partitions, one for each noncoding gene (12S and 16S) and one for each codon of protein-coding genes (COI, CYTB and ND1). The following best-fit evolutionary models and partition schemes were determined through ModelFinder (Kalyaanamoorthy et al., 2017): TIM2+F+R5 for 16S, GTR+F+I+G4 for 12S, TIM3e+I+G4 for COI 1st codon, HKY+F+I+G4 for COI 2nd codon plus CYTB 2nd codon, TN+F+R4 for COI 3rd codon, GTR+F+I+G4 for ND1 1st–3rd codons plus CYTB 1st codon, and TIM+F+R4 for CYTB 3rd codon. Phylogenetic relationships were reconstructed using Maximum Likelihood inference (ML) in IQ-TREE (Trifinopoulos et al., 2016). Clade support was calculated through 10,000 ultrafast bootstrap approximation replicates (Hoang et al., 2018) with 10,000 maximum iterations (number of unsuccessful iterations to stop ≥ 100), a 0.99 minimum correlation coefficient and 10,000 replicates of the Shimodaira-Hasegawa approximate likelihood ratio test. Uncorrected (p) and Kimura two-parameter (K2P) genetic distances (Kimura, 1980) between the new species and other nurse frogs were calculated with MEGA 6 (Tamura et al., 2013).

### Morphology

Males and females were sampled during breeding season, and individuals collected while calling, mating, or carrying tadpoles on their back were considered to be adults. Sex was determined in the field based on breeding behavior (advertisement call by males, females surrounding active males, amplexus) and in the laboratory by examination of secondary sexual characters (*e.g.*, vocal sac in males, eggs visible through the belly skin in females). External morphology was assessed from 49 males and 26 females. Terminology, diagnostic characteristics and morphometric measurements follow Caldwell & Lima (2003), Grant et al. (2006) and Lima, Sanchez & Souza (2007). The following 23 measurements, accurate to the nearest 0.1 mm, were taken by using digital calipers or a micrometer coupled to a stereoscopic microscope: snout-vent length (SVL); head length (HL); head width (HW); snout length (SL); interorbital distance (IO); eye–nostril distance (EN); internarial distance (IN); eye diameter (EL); tympanum diameter (TYM); forearm length (FAL); arm length (AL); thigh length (THL); tibia length (TL); foot length (FL); hand length from the proximal edge of the palmar tubercle to the tip of fingers I–IV (HANDI–HANDIV); width of the disc on finger III (WFD); diameter of palmar tubercle (DPT); width of thenar tubercle at middle section (WTT); width of the third phalange of finger III at its mid-level (WPF); and width of the disc on toe IV (WTD). Raw data are provided in Table S2. Description of adult coloration in life is described from field notes and digital photographs by A. P. Lima. Description of adult morphology follows Lima, Simões & Kaefer (2014).

Tadpole developmental stages follow Gosner (1960). Terminology, diagnostic characters and morphometric measurements follow Altig & McDiarmid (1999) and Schulze, Jansen & Köhler (2015). The following 17 morphometric measurements were taken from 51 tadpoles: total length from tail tip to tip of snout (TL); body length from tip of snout to body-tail insertion (BL); tail length from tail tip to body-tail insertion (TAL); body width at level of spiracle (BW); body height at level of spiracle (BH); head width at level of eyes (HWLE); maximum width of tail muscle (TMW); maximum height of tail (MTH); maximum height of tail muscle (TMH); interorbital distance (IOD); internarial distance (IND); eye–nostril distance (END); nostril-snout distance (NSD); eye diameter (ED); vent-tube length (VTL), spiracle-tube length (STL); and oral disc width (ODW). Raw data are provided in Table S3.

### Bioacoustics

The advertisement call of the new species comprises a set of multiple notes separated by short silent intervals. Whenever possible, five calls of each structural arrangement were measured for each recorded male, totaling 476 analyzed calls. Acoustic parameters were obtained through Raven 1.5 with the following configuration: Window = Blackman, Discrete Fourier Transform = 1,024 samples and 3 dB filter bandwidth = 82 Hz. The following parameters were measured for each call: (CD) call duration, (ICI) inter-call interval, (NNC) notes per calls, (ND) note duration (quantified for the first, middle and last notes), (INOD) inter-note intervals (quantified between the first and second notes and before the last note) and (DF) dominant frequency. Dominant frequency was obtained using the function *Peak frequency* of Raven. These acoustic parameters were also measured for a recorded male of *Allobates tapajos* sensu lato from the *Jací* sampling module. Bioacoustic raw data are provided in Tables S4 and S5.

Call terminology and description follow Köhler et al. (2017) and Lima, Simões & Kaefer (2015), respectively. Graphic representation of advertisement calls was produced in R (R Core Team, 2021) using the package *seewave* v.2.1 (Sueur, Aubin & Simonis, 2008). *Seewave* was set up as follows: window = Hanning, FFT size = 256 samples, and FFT overlap = 85%.

### Species delimitation

Three single-locus methods of molecular species delimitation were implemented to infer putative species in *Allobates tapajos*: multi-rate Poisson Tree Processes (mPTP; Kapli et al., 2017), Generalized Mixed Yule Coalescent (GMYC; Pons et al., 2006; Fujisawa & Barraclough, 2013) and Assemble Species by Automatic Partitioning (ASAP; Puillandre, Brouillet & Achaz, 2021). mPTP considers the branch lengths in a phylogenetic tree and assumes that the substitution rate is significantly larger between heterospecific samples than between conspecific samples (Zhang et al., 2013; Kapli et al., 2017). GMYC uses speciation and neutral coalescent models to delimit genetic clusters by optimizing the set of nodes that define the transitions between inter-and intra-specific processes (Pons et al., 2006; Fujisawa & Barraclough, 2013). ASAP is a hierarchical clustering algorithm that uses pairwise genetic distances to estimate multiple potential barcode gaps and defines minimum distance thresholds, which are used to partition samples into putative species; selection of the best species partition in ASAP is based on a score system (Puillandre et al., 2012). mPTP and the single-threshold GMYC were run using default parameters in the webservers https://mptp.h-its.org/ and https://species.h-its.org/gmyc/, respectively; ASAP was run using uncorrected distances as the substitution model in the webserver https://bioinfo.mnhn.fr/abi/public/asap/.

As input mPTP needs a phylogenetic tree where branch lengths represent substitutional distances, therefore a 16S rRNA maximum likelihood tree was inferred through IQ-TREE using the GTR substitution model. Clade support was calculated through 1,000 ultrafast bootstrap approximation replicates with a maximum 1,000 iterations and a 0.99 minimum correlation coefficient. GMYC requires a single-locus, ultrametric phylogenetic tree as input. This tree was inferred through Bayesian inference in BEAST 2.6.3 (Bouckaert et al., 2019) using a lognormal uncorrelated relaxed clock model and Yule process tree prior; a run of 100 million generations was sampled every 10,000 steps. Stationarity and effective sample size of posterior distributions and priors were examined using Tracer v1.7.1 (Rambaut et al., 2018). Resulting trees were annotated into a maximum clade credibility (MCC) tree after discarding the first 10% using TreeAnotator v.2.6.3 (Bouckaert et al., 2019).

Although ratios are widely used in taxonomy to represent body shape and differentiate taxa, their use in some multivariate analyses (*e.g.*, principal component analysis; PCA) has been criticized mostly because the ratio X/Y is not usually a linear function of X and Y (see Atchley, Gaskins & Anderson, 1976). Nevertheless, this bias disappears in most cases by using log(X/Y) (Hills, 1978). To avoid issues related to nonlinearity of raw ratios in multivariate analyses (*e.g.*, HL/SVL, HW/SVL, FL/SVL), we performed multivariate ratio analysis (MRA; Baur & Leuenberger, 2011), a method that consists of algorithms adapted to both PCA (hereafter, shape PCA) and linear discriminant analysis (hereafter, LDA ratio extractor). While shape PCA allows the interpretation of principal components in terms of body shape and isometric size (isosize), the LDA ratio extractor reveals the morphometric ratios that most effectively separate groups. Analyses were run using the R script developed by Baur et al. (2014) and Baur & Leuenberger (2020) and provided in Script S1. Analyses of variance (ANOVAs) were then performed with the first two components from shape PCA and isosize as response variables, and clades as factors; statistical significance was calculated through Tukey’s test. ANOVAs and Tukey’s test were performed using the functions *aov* and *TukeyHSD* of the package Stats 3.4.1 (R Core Team, 2021), respectively. The number of retained components of shape and standard PCAs for further analyses were selected through scree plot inspection.

Shape PCA and LDA ratio extractor were conducted separately for males and females using 23 morphometric measurements (DPT, EL, EN, FAL, FL, HANDI–HANDIV, HL, HW, IN, IO, SL, SVL, THL, TL, TYM, UAL, WFD, WPF, WTD and WTT) of *Allobates tapajos* sensu stricto (clade A) and *A. tapajos* sensu lato (clade E) from the upper Madeira River (UMR). Morphometric measurements of adult *A. tapajos* sensu stricto were taken from 14 males (INPAH 34402–06, 34410–12, 34414–16, 34418, 34423 and 34425) and 10 females (INPAH 34407–09, 34413, 34417, 34419–22 and 34424) of the type series of *A. tapajos*.

To test for statistical difference between the bioacoustic multidimensional spaces occupied by *Allobates tapajos* sensu stricto (clade A) and *A. tapajos* sensu lato (clade E) from UMR, we performed a standard PCA associated with ANOVAs in which the first two PCs were treated as response variables and clades as factors; statistical significance was calculated through Tukey’s test. Five acoustic parameters (CD, ICI, ND, INOD and DF) were measured from 2-note calls of the holotype (INPAH 34425) and six paratypes (INPAH 34402, 34405, 34410, 34414–16) of *A. tapajos* (call vouchers FNJV 50556–62). Acoustic analyses were conducted only with parameters from 2-note calls because this is the most common structural arrangement among all recordings of *A. tapajos* sensu stricto.

### Species comparisons

Phenotypic comparisons between the new species and congeners were restricted to species of *Allobates* (Appendix 1) distributed in southwestern Amazonia (*A. caldwellae* Lima, Ferrão & Silva, 2020; *A. conspicuus* (Morales, 2002); *A. femoralis* Boulenger, 1884; *A. flaviventris* Melo-Sampaio, Souza & Peloso, 2013; *A. hodli* (Simões, Lima & Farias, 2010); *A. nidicola* (Caldwell & Lima, 2003); *A. pacaas* (Melo-Sampaio et al., 2020); *A. sieggreenae* Gagliardi-Urrutia et al., 2021; *A. subfolionidificans* (Lima, Sanchez & Souza, 2007); *A. tinae* (Melo-Sampaio, Oliveira & Prates, 2018); *A. trilineatus* (Boulenger, 1884), *A. velocicantus* Souza et al., 2020) and to phylogenetically close relatives belonging to the *A. caeruleodactylus* clade (*A. caeruleodactylus* (Lima & Caldwell, 2001); *A. gasconi* (Morales, 2002); *A. grillisimilis* Simões et al., 2013; *A. grillicantus* Moraes & Lima, 2021; *A. paleci* Silva et al., 2022; and *A. tapajos* Lima, Simões & Kaefer, 2015).

### Intraspecific variation

Sexual dimorphism is common in most anuran lineages (Shine, 1979) and evident in several morphological traits (Kupfer, 2007). Sexual dimorphism in SVL and 22 morphometric ratios of 26 females and 49 males of the new species was assessed through two-way ANOVAs for unbalanced design using the function *Anova* of the package *car* (Fox & Weisberg, 2019). Morphological traits were treated as response variables while sex and its interaction with riverbank were treated as factors. Prior to two-way ANOVAs, morphometric ratios were log-transformed to follow normal distribution, then scaled and centered using the function *scale* of the package *base* (R Core Team, 2021). Homogeneity of variances was visually inspected and tested through residuals vs. fitted values plot and Levene’s test (function *leveneTest* of the *car* package), respectively. Residuals’ normal distributions were inspected through Normal Q-Q plots and tested using the Shapiro–Wilk test (function *shapiro.test* of the *stats* package; R Core Team, 2021). Outliers were excluded from analyses to fit homogeneity and normality assumptions. *P*-values were adjusted for multiple comparisons using Benjamini & Hochberg correction (Benjamini & Hochberg, 1995) through the function *p.adjust* of the package *stats*.

Amazonian rivers exhibit contrasting effects on intraspecific morphological variation of anurans. For example, there is significant variation between populations of *Allobates femoralis* on opposite banks of the Madeira River (Simões et al., 2008) but not between populations of the *A. nidicola*–*masniger* complex (Kaefer et al., 2013). In the present study, we used shape and standard PCAs associated with ANOVAs to test whether shape, isosize and bioacoustic spaces occupied by the new species differ between opposite banks of the upper Madeira River. Shape PCA was conducted with 23 morphometric measurements (described above) and run separately for males and females. Standard PCA was conducted with five acoustic parameters (described above) of 16 and 14 recorded males from west and east banks, respectively. ANOVAs were then run with the first two shape PCs and isosize from shape PCA, and PC1–2 from standard PCA; PCs were treated as response variables and riverbank as factors.

### Nomenclatural acts

The electronic version of this article in Portable Document Format (PDF) will represent a published work according to the International Commission on Zoological Nomenclature (ICZN), hence the new names contained in the electronic version are effectively published under that Code from the electronic edition alone. This published work and the nomenclatural acts it contains have been registered in ZooBank, the online registration system for the ICZN. ZooBank LSIDs (Life Science Identifiers) can be resolved and the associated information viewed through any standard web browser by appending the LSID to the prefix http://zoobank.org/. The LSID for this publication is: urn:lsid:zoobank.org:pub:E2CB2367-561F-461E-87A4-672AC6205875. The online version of this work is archived and available from the following digital repositories: PeerJ, PubMed Central and CLOCKSS.

## Results

### Phylogenetic relationships and genetic distances

*Allobates* is recovered as monophyletic with high support in the multilocus phylogeny (Fig. S1). The composition of all major clades is mostly similar to that of the mitogenomic phylogeny of Réjaud et al. (2020). The main difference regards the monophyly of the *A. caeruleodactylus* clade, which is recovered as paraphyletic in the multilocus phylogeny; the clade grouping *A. tapajos* and *A. gasconi* species complexes is placed apart from the clade grouping *A. caeruleodactylus*, *A. grillicantus*, *A. grillisimilis* and *A. paleci* (Fig. S1).

**Figure 1:**
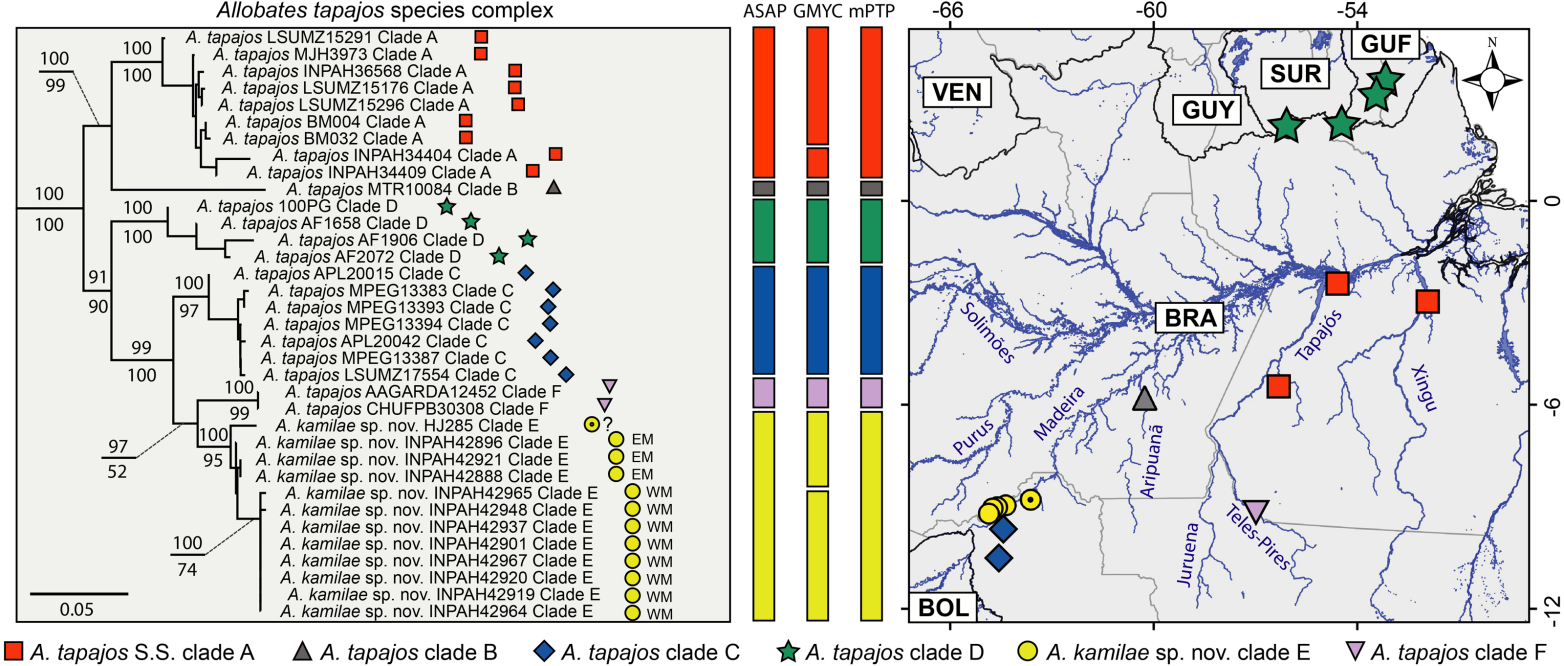
Maximum likelihood phylogenetic tree (left) based on five mitochondrial genes (12S, 16S, COI, ND1 and CYTB) and geographic distribution of the *Allobates tapajos* species complex. Bootstrap and SH-aLRT support of major clades is shown above and below nodes, respectively. Vertical bars show single-locus (16S) species delimitation through Assemble Species by Automatic Partitioning (ASAP), Generalized Mixed Yule Coalescent (GMYC) and Multi-rate Poisson Tree Processes (mPTP). The black-dotted yellow circle denotes the record of Vacher et al. (2020) for which geographic coordinates are uncertain. Relationships between the *A. tapajos* species complex and close relatives are illustrated in Fig. S1. Country abbreviations: BOL, Bolivia; BRA, Brazil; GUF, French Guiana; GUY, Guyana; SUR, Surinam; VEN, Venezuela.

Six major clades are recovered within the *Allobates tapajos* species complex (here identified as clades A–F). Clade A groups two paratypes of *A. tapajos* (APL12965 and 12968) with individuals from the lower Tapajos and Xingu rivers (Fig. 1) and nests with high support as sister to *A. tapajos* clade B from Aripuanã River, central Amazonia. All specimens from the banks of the upper Madeira River clump with specimen HJ 285 reported by Vacher et al. (2020); together, they comprise *A. tapajos* clade E (Fig. 1). This clade is sister to *A. tapajos* clade F, which clusters specimens from the Teles-Pires River, southeastern Amazonia. The other population from Madeira Basin (Jací-Paraná River and Guajará-Mirim State Park, Rondônia) comprises *A. tapajos* clade C and is recovered as sister to clade E+F. This major clade is recovered as sister to *A. tapajos* clade D, the only population distributed across the Guiana Shield, northeastern Amazonia.

Pairwise K2P genetic distances are low between specimens of *Allobates tapajos* clade E from the upper Madeira River, both between opposite banks (0.7–1.1%) and within banks (0.0–0.2% in the east bank; 0.0–0.4% in the west bank). The distance between the specimen reported by Vacher et al. (2020) (HJ 285) and our samples of *A. tapajos* clade E from east (0.6–0.8%) and west banks (1.5–1.7%) is relatively low. This evidence leads us to regard them as conspecific, yet suggests that HJ 285 belongs to the east population. The average distance between *A. tapajos* clade E and *A. tapajos* sensu stricto (clade A) is 5.9% (range 4.8–7.9%), and between *A. tapajos* clade E and its sister clade F is 3.8% (3.2–4.0%). Finally, the average distance between *A. tapajos* clade E and clade C, its geographically closest sibling, is 3.8% (2.6–4.8%). Table 1 lists K2P and p genetic distances between all clade pairs.

**Table 1:** Kimura two-parameter (K2P; upper diagonal) and uncorrected (p-distance; lower diagonal) pairwise genetic distances between species of the *Allobates tapajos* species complex included in our phylogenetic analyses. Clade A = *A. tapajos* sensu stricto, clade E = *A. kamilae* sp. nov. Distances are based on a 569 base-pair fragment of the 16S rRNA mitochondrial gene. Values depict mean percent distance (range). Intraspecific p-distances are in boldface.

Clades	Clade A	Clade B	Clade C	Clade D	Clade E	Clade F
*A. tapajos* clade A	**0.9 (0.0–2.5)**	5.1 (4.2–5.9)	5.8 (4.3–7.5)	6.7 (5.4–8.1)	5.6 (4.6–7.4)	5.2 (4.9–5.8)
*A. tapajos* clade B	5.3 (4.3–6.2)	–	7.2 (5.6–8.0)	6.1 (5.6–7.1)	5.9 (5.4–7.7)	5.9 (5.9–5.9)
*A. tapajos* clade C	6.1 (4.5–7.9)	7.6 (5.9–8.6)	**0.6 (0.0–1.1)**	5.4 (3.6–7.3)	3.7 (2.5–4.6)	3.3 (2.8–4.2)
*A. tapajos* clade D	7.1 (5.7–8.7)	6.4 (5.9–7.5)	5.7 (3.7–7.7)	**0.1 (0.0–0.3)**	5.0 (3.5–7.7)	4.6 (3.6–5.2)
*A. tapajos* clade E	5.9 (4.8–7.8)	6.2 (5.6–8.2)	3.8 (2.6–4.8)	5.2 (3.6–8.1)	**0.6 (0.0–1.7)**	3.5 (3.1–3.8)
*A. tapajos* clade F	5.4 (5.1–6.1)	6.2 (6.2–6.2)	3.4 (2.9–4.4)	4.8 (3.7–5.4)	3.7 (3.2–4.0)	**0.0 (0.0–0.0)**

### Species delimitation

According to the best-ranked partition computed by ASAP (score = 4.0; *p* = 0.240; *w* = 0.0003), the best threshold distance for the 16S dataset is 2.1%. In this partition, all six clades of the *Allobates tapajos* species complex are delimited as distinct putative species: *A. tapajos* sensu stricto (clade A) and five candidate species (clades B–F; Fig. 1). The same six putative species are delimited by mPTP (Fig. 1). GMYC is less conservative than ASAP and mPTP in delimiting eight putative species by splitting *A. tapajos* clades A and E into two putative species each (Fig. 1). ASAP and mPTP delimit as conspecific specimen HJ 285 (Vacher et al. (2020) and those of *A. tapajos* clade E from both banks of the upper Madeira River. GMYC, however, delimits HJ 285 and specimens from the east bank as heterospecific to specimens from the west bank (Fig. 1).

**Figure 2:**
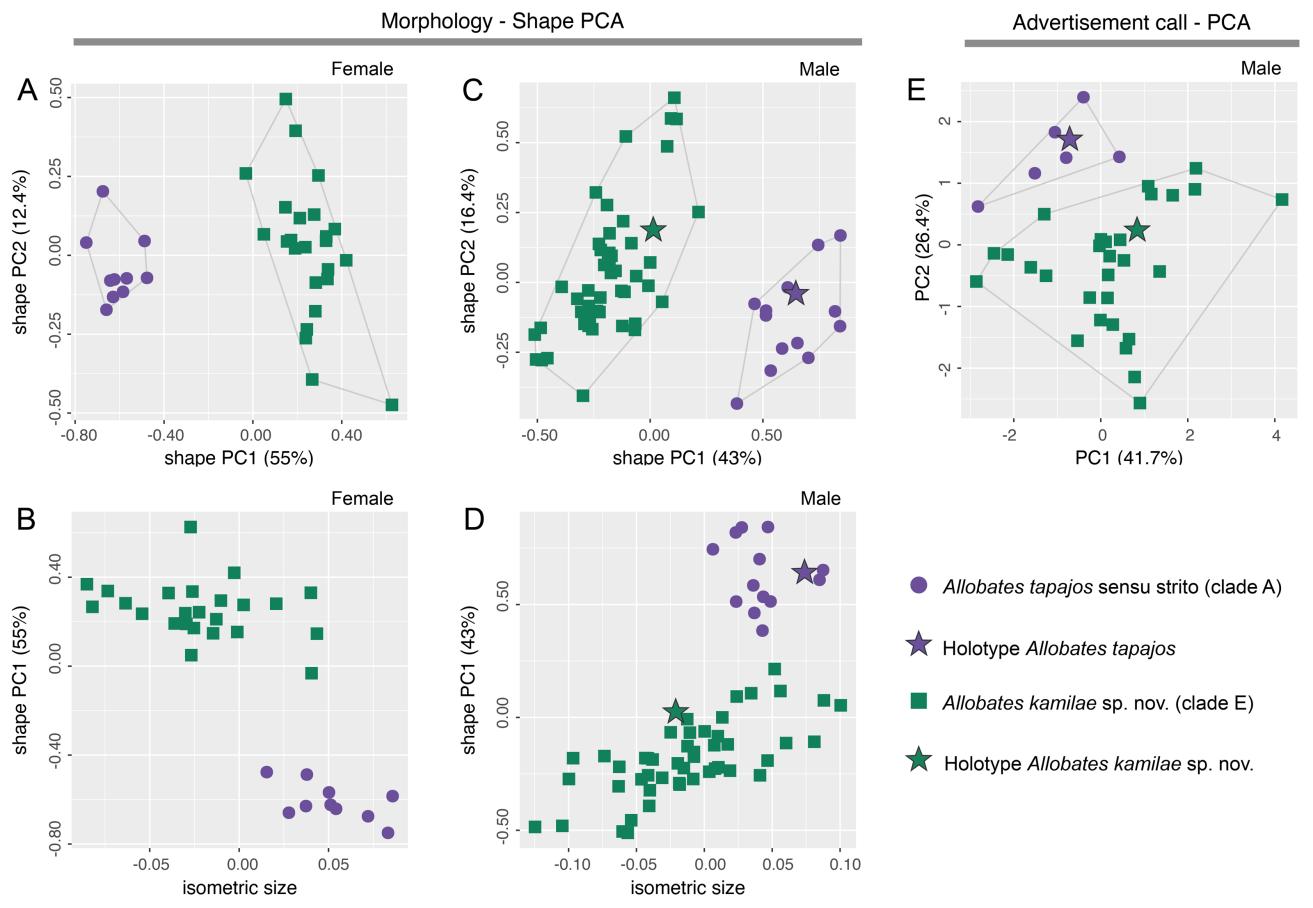
Shape (A–D) and standard (E) principal component analyses (PCA) of morphological and bioacoustic data of *Allobates tapajos* clade A (type series of *A. tapajos*) and clade E (*Allobates kamilae* sp. nov.). Morphological analyses are based on 23 morphometric measurements; the bioacoustic analysis is based on five acoustic parameters.

There is no overlap of body shape of either males (ANOVA: *S*^2^ = 7.182, *F* = 273, *df* = 61, *p* < 0.0001) or females (ANOVA: *S*^2^ = 5.263, *F* = 378, *df* = 32, *p* < 0.0001) between *Allobates tapajos* clades A and E in the first principal component (PC) of shape PCAs (Figs. 2A, 2C). Unlike in females, body shape of males of clades A and E also differs in the second shape PC (ANOVA: *S*^2^ = 0.290, *F* = 5.798, *df* = 61, *p* = 0.02). The isosize of both females (ANOVA: *S*^2^ = 0.038, *F* = 35.27, *df* = 32, *p* < 0.0001) and males (ANOVA: *S*^2^ = 0.035, *F* = 17.31, *df* = 61, *p* = 0.0001) also differs between clades, being larger in clade A (Figs. 2B, 2D). Table S6 reports the variation explained by the first two shape PCs and the loadings of morphometric variables.

The LDA ratio extractor reveals that EN/WFD (standard distance = 20.5; *δ* = 0.100) and FAL/HL (standard distance = 18.2; *δ* = 0.111) are the most discriminating body ratios separating females of clades A and E. On the other hand, most discriminating body ratios for males were WFD/WTD (standard distance = 11.5; *δ* = 0.100) and HANDIV/WFD (standard distance = 8.7; *δ* = 0.127). Low values of *δ* (0.100–0.127) indicate that discrimination between clades A and E is driven mainly by body shape and not body size, whereas high values of standard distances (8.7–20.5) reflect the power of those body ratios to discriminate taxa.

While there is slight overlap of the multidimensional bioacoustic spaces occupied by 2-note calls of *A. tapajos* clades A and E in the first two PCs of the standard PCA (Fig. 2E), the spaces occupied are significantly different in both PC1 (ANOVA: *S*^2^ = 8.24, *F* = 4.32, *df* = 35, *p* = 0.045) and PC2 (ANOVA: *S*^2^ = 19.19, *F* = 23.71, *df* = 35, *p* < 0.0001). Inter-note interval and inter-call interval are the most important bioacoustic variables contributing to PC1 and PC2, respectively. Table S7 reports the variation explained by each PC and the loadings of all bioacoustic variables.

Although not evaluated with a multivariate statistical approach, the advertisement call of *Allobates tapajos* clade E is easily distinguished from the calls of clades C and F with respect to call arrangements. Average note duration in 2-note calls of *A. tapajos* clade E (31 ± 6 ms) is shorter than in clade F (43 ± 3 ms), and average note duration in 3-note calls is longer in the former (140 ± 27 ms) than in the latter (86 ± 18 ms). On the other hand, average call duration and inter-note interval in 2- and 3-note calls of *A. tapajos* clade E are longer than in clade C (Table 2). Finally, calls of *A. tapajos* clade A also differs from those of clades C and F in temporal parameters, mainly call duration and inter-note interval (Table 2).

Recognizing the concordance of morphological, bioacoustic and molecular evidence, *Allobates tapajos* clade E from both banks of the upper Madeira River is described below as a new species. Molecular species delimitation further identified *A. tapajos* clades B–D and F as putative species, an hypothesis that is supported by bioacoustic data for clades C and F. Nevertheless, we refrain from formally describing these candidate species at this time pending acquisition of additional data that bolsters the above hypothesis.

## Taxonomy

### *Allobates kamilae* sp. nov.

LSID: urn:lsid:zoobank.org:act:350D7688-B957-41EF-AD81-82A417661ED9

**Table:** 

*Allobates* sp. 1: Dias-Terceiro et al. (2015), Dayrell et al. (2021).
*Allobates tapajos* “Allobates_63”: Vacher et al. (2020), SupInfo 4.
*Allobates* aff. *tapajos*: Moraes & Lima (2021), Silva et al. (2022).
*Allobates* aff. *tapajos* “3”: Réjaud et al. (2020), Fig. 1 and Figs. S1–S3.

### Holotype

INPAH 42923 (field number APL 14840), an adult male collected 10 March 2010 by A. P. Lima from the *Búfalos* sampling module (−9.133275, −64.497260; 102 masl), west bank of the upper Madeira River, municipality of Porto Velho, state of Rondônia, Brazil.

**Table 2:** Advertisement call parameters of four species of the *Allobates tapajos* species complex. Clade A = *A. tapajos* sensu stricto, clade E = *A. kamilae* sp. nov. Values depict mean ± standard error (range).

	Parameters	*A. tapajos* clade A (*n* = 7)	*A. tapajos* clade C (*n* = 1)	*A. tapajos* clade E (*n* = 30)	*A. tapajos* clade F (*n* = 9)
Two-note call	Call duration (ms)	211 ± 21 (169–280)	133 ± 5 (130–141)	211 ± 35 (141–333)	197 ± 19 (155–229)
	Note duration (ms)	38 ± 4 (30–45)	27 ± 1 (25–29)	31 ± 6 (19–47)	43 ± 3 (38–55)
	Inter-note interval (ms)	136 ± 16 (101–196)	79 ± 5 (74–86)	148 ± 29 (85–241)	107 ± 15 (74–138)
	Inter-call interval (ms)	442 ± 146 (315–1,188)	447 ± 86 (363–579)	692 ± 299 (302–2,077)	453 ± 45 (361–579)
	Dominant frequency (Hz)	5,631 ± 278 (5,124–6,072)	5,469 ± 30 (5,426–5,512)	5,744 ± 274 (5,211–6,201)	5,813 ± 123 (5,512–6,030)
Three-note call	Call duration (ms)	380 ± 8 (321–341)	242 ± 10 (231–259)	376 ± 60 (260–593)	299 ± 41 (252–418)
	Note duration (ms)	33 ± 2 (29–35)	29 ± 2 (26–34)	32 ± 6 (18–49)	41 ± 4 (30–52)
	Inter-note interval (ms)	115 ± 10 (104–137)	78 ± 6 (66–88)	140 ± 27 (96–247)	86 ± 18 (60–152)
	Inter-call interval (ms)	453 ± 88 (316–546)	385 ± 28 (362–418)	673 ± 299 (309–2,236)	431 ± 63 (341–609)
	Dominant frequency (Hz)	5,435 ± 36 (5,383–5,469)	5,469 ± 30 (5,426–5,512)	5,736 ± 267 (5,211–6,201)	5,728 ± 84 (5,512–6,030)
Four-note call	Call duration (ms)	–	–	527 ± 73 (387–794)	406 ± 11 (393–421)
	Note duration (ms)	–	–	32 ± 5 (19–49)	43 ± 3 (40–50)
	Inter-note interval (ms)	–	–	136 ± 28 (94–268)	77 ± 6 (67–90)
	Inter-call interval (ms)	–	–	678 ± 238 (299–1,778)	413 ± 37 (374–482)
	Dominant frequency (Hz)	–	–	5,719 ± 248 (5,211–6,201)	5,685 ± 0 (5,685–5,685)

**Notes.**

Abbreviations n number of males analyzed Hz Hertz

### Paratopotypes

Eight adults collected by A. P. Lima, same locality as the holotype. Six males (INPAH 42963, 42919 (GenBank MZ667626), 42902, 42937 (GenBank MZ667628), 42952, 42943 (field numbers APL 14789, 14791, 14837–39 and 14841, respectively)) and one female (INPAH 42964 (field number APL 14758, GenBank MZ667627)) collected in March 2010; one male, INPAH 42886 (field number APL 16854) collected 25 January 2011.

### Paratypes

Sixty-six adults collected by A. P. Lima in the upper Madeira River, municipality of Porto Velho, state of Rondônia, Brazil. *Abunã Esquerdo* sampling module (−9.516000, −65.324900; 116 masl): five females (INPAH 42931, 42906, 42938, 42889, 2950 (field numbers APL 2197–98, 2222, 2235 and RM 57, respectively)) and seven males (INPAH 42882, 42956, 42881, 42944, 42887, 42926 and 42936 (field numbers APL 2233–34, 2236–37, RM 81, 83 and 643, respectively)), collected in December 2004. *Jirau Esquerdo* sampling module (−9.317073, −64.743354; 136 masl): two female (INPAH 42912 and 42949 (field numbers APL 16349 and 14732)) and six males (INPAH 42913, 42917, 42967 (GenBank MZ667632), 42899, 42965 (GenBank MZ667629) and 42945 (field numbers APL 14593, 14608–09, 14630, 14700 and 14702, respectively)), collected in February 2010 and November 2010. *Pedras* sampling module (−9.167074, −64.629109; 103 masl): two females (INPAH 42925 and 42939 (field numbers APL 14959 and 16962, respectively)) and six males (INPAH 42920 (GenBank MZ667631), 42948 (GenBank MZ667630), 42966, 42947, 42929 and 42879 (field numbers APL 14970, 14972, 14973–75 and 16963, respectively)), collected in March 2010 and January 2011. *Abunã Direito* sampling module (−9.618408, −65.391275; 114 masl): three males (INPAH 42928, 42900 and 42932 (field numbers APL 2240 and 2242–43, respectively)) and six females (INPAH 42958, 42933, 42885, 42916, 42914 and 42953 (field numbers APL 2244–45, 2247; RM 280–81 and 309, respectively)), collected in December 2004. *Jirau Direito* sampling module (−9.361940, -64.691940; 132 masl): five females (INPAH 42962, 42934, 42915, 42908 and 42877 (field numbers APL 2041–43, 16612 and 16617, respectively)) and twelve males (INPAH 42911, 42924, 42930, 42935, 42959, 42880, 42892, 42897, 42904, 42878, 42905 and 42955 (field numbers APL 2241, 2040, 14592, 14751, 16413, 16421, 16611, 16615–16, 16619–20 and RM 781, respectively)), collected in November 2004, March 2010 and January 2011. *Morrinhos* sampling module (−9.076111, −64.246111; 105 masl): five females (INPAH 42894, 42941–42, 42940 and 42927 (field numbers APL 15832, 16433–34 and 16461–62, respectively)) and seven males (INPAH 42893, 42909, 42890, 42957, 42884, 42903 and 42961 (field numbers APL 15848, 15878, 15887, 15917, 16432, 16464 and RM 476, respectively)), collected in November 2010 and January 2011.

### Additional material

Six adults from the upper Madeira River, municipality of Porto Velho, state of Rondônia, Brazil. *Jirau Esquerdo* sampling module: INPAH 42901 (GenBank MZ667633, field number APL 14701), collected by A. P. Lima. *Morrinhos* sampling module: INPAH 42921 (GenBank MZ667620), 42888 (GenBank MZ667621), 42896 (GenBank MZ667622), 42895 (GenBank MZ667623) and 42922 (GenBank MZ667625) (field numbers APL 15879, 22564–65, 22570 and 22572, respectively) and APL22571 (GenBank MZ667624), collected by A. P. Lima, M. Ferrão and J. S. Dayrell.

### Etymology

The specific epithet *kamilae* honors Kamila Xavier Amaral, a very young and enthusiastic biologist who passed away prematurely after battling cancer. While an undergraduate student, Kamila was being trained in *Allobates* taxonomy by A. P. Lima. She was the first person to study the new species described in this study.

### Generic placement

The new species is assigned unambiguously to the genus *Allobates* because the tip of finger IV does not reach the distal subarticular tubercle of finger III, toe IV with basal webbing and lateral fringe on its preaxial side, pale paracloacal marks (characters 5, 43 and 50 of Grant et al., 2017), respectively) and its phylogenetic relationship as assessed by molecular data.

### Diagnosis

A small-bodied species of *Allobates* characterized by the following combination of characters: SVL 14.5–17.4 mm in males (*n* = 49) and 15.2–17.8 mm in females (*n* = 26); finger III not swollen in males; one subarticular tubercle on finger IV; basal webbing between toes II and III absent; EN/WFD = 2.67–3.67 in females; WFD/WTD = 0.67–1.00 and HANDIV/WFD = 3.58–5.60 in males; dorsal surface of thigh lacks red or yellow marks; pale dorsolateral stripe absent in preservative; white ventrolateral stripe absent in life; belly without dark and white marbling; throat in males bright yellow with scattered melanophores; throat in females with scattered melanophores; dorsal surface of fingers brown to orange-brown; advertisement calls predominantly arranged in groups of 2–5 unpulsed notes; first (135 ms ± 24) and last (142 ms ± 31) inter-note intervals are of similar duration and longer than note duration; dominant frequency 5,211–6,201 Hz; tadpoles have an oral disc and spiracle; five short papillae on each side of the lateral margin of the anterior labium; long papillae on the posterior labium; a “V”-shaped lower jaw sheath; and labial tooth rows P-3 and P-1 of similar length.

### Species comparisons

Characters of compared species are enclosed in parentheses if not otherwise stated. Morphological, bioacoustic and tadpole comparisons are presented separately.

*Allobates kamilae* sp. nov. is most similar to *A. tapajos* sensu stricto. However, *A. kamilae* sp. nov. differs from *A. tapajos* sensu stricto by the absence of a basal membrane between toes II and III (present), EN/WFD = 2.67–3.67 in females (EN/WFD = 1.15–1.50), WFD/WTD = 0.67–1.00 and HANDIV/WFD = 3.58–5.60 in males (1.43–2.00 and 1.92–2.70, respectively), and by a pale dorsolateral stripe absent in preservative (present), a bright yellow throat in males with scattered melanophores in life (bright yellow without melanophores), and scattered melanophores on the throat of females (melanophores absent).

*Allobates kamilae* sp. nov. differs from both *A. femoralis* and *A. hodli* by the absence of both red or yellow marks on the dorsal surface of the thigh and light and dark marbling on the anterior portion of the belly (present in both species) and by the maximum SVL 17.4 mm in males (minimum SVL 22.2 mm in *A. hodli*, 22.3 mm in *A. femoralis*; Simões et al., 2008; Simões, Lima & Farias, 2010); from *A. caeruleodactylus* by having brown to orange-brown fingers in life (sky-blue fingers); from *A. nidicola* by the maximum SVL 17.4 mm in males (minimum SVL 18.5 mm); from *A. flaviventris* by the maximum SVL of 17.8 mm in females (minimum SVL 19.3 mm); from *A. pacaas* by having one subarticular tubercle on finger IV (two subarticular tubercles); from *A. gasconi* by the absence of a swollen finger III in males (present); from *A. flaviventris*, *A. gasconi*, *A. grillicantus*, *A. grillisimilis*, *A. nidicola*, *A. pacaas*, *A. paleci*, *A. sieggreenae*, *A. subfolionidificans*, *A. tinae* and *A. trilineatus* by having males with a bright yellow throat with scattered melanophores in life (bright yellow without melanophores in *A. caldwellae*, violet-gray in *A. flaviventris*, white in *A. grillisimilis* and *A. subfolionidificans*, pale yellow without melanophores in *A. grillicantus*, light gray to black in *A. nidicola*, violet in *A. pacaas*, yellowish without melanophores in *A. paleci*, white with scattered melanophores in *A. sieggreenae*, and gray to dark gray in *A. gasconi* and *A. trilineatus*); from *A. caldwellae*, *A. conspicuus*, *A. grillicantus*, *A. sieggreenae*, *A. tinae*, *A. trilineatus* and *A. velocicantus* by the absence of a pale dorsolateral stripe in preservative (present in all species); from *A. caldwellae*, *A. conspicuus*, *A. grillicantus*, *A. grillisimilis*, *A. sieggreenae*, *A. tinae*, *A. trilineatus* and *A. velocicantus* by the absence of a pale ventrolateral stripe in life (present in all species).

Advertisement calls of *Allobates kamilae* sp. nov. and *A. tapajos* sensu stricto are similar: both are characterized by the continuous emission of notes arranged in groups. However, *A. tapajos* sensu stricto mostly emits groups of two notes and rarely groups of three notes, whereas *A. kamilae* sp. nov. calls mainly in groups of two, three and four notes and rarely in groups of up to 17 notes. *Allobates kamilae* sp. nov. differs from *A. caeruleodactylus*, *A. nidicola* and *A. subfolionidificans* (calls composed exclusively by a single note in all species); from *A. paleci* (17–61 notes) and *A. velocicantus* (66–138 notes); from *A. femoralis*, *A. flaviventris*, *A. hodli* and *A. nidicola* by its dominant frequency of 5,211–6,201 Hz (3,100–3,375 Hz in *A. femoralis*, 3,6178–4,651 Hz in *A. flaviventris*, 2,991–3,897 Hz in *A. hodli* and 3,759–4,689 Hz in *A. nidicola*); from *A. sieggreenae* by having an inter-note interval of 85–268 ms (260–1,720 ms); from *A. caldwellae* and *A. tinae* by having the first (135 ms ± 24) and last (142 ms ± 31) inter-note intervals with similar duration (124 ms ± 11 and 178 ± 27 in *A. caldwellae*, 126 ms ± 19 and 276 ± 58 in *A. tinae*); from *A. grillicantus* and *A. grillisimilis* by presenting inter-note intervals longer than the note duration (inter-note interval similar to note duration in both species); and from *A. trilineatus* by having unpulsed notes (pulsed notes). Advertisement calls of *A. conspicuus*, *A. gasconi* and *A. pacaas* are unknown.

Tadpoles of *Allobates kamilae* sp. nov. differ from those of *A. tapajos* sensu stricto by having labial tooth rows P-3 and P-1 of similar length (P-3 shorter than P-1) and A-2 and A-1 about the same length (A-2 longer than A-1). Tadpoles of *A. kamilae* sp. nov. differ from those of *A. nidicola* by the presence of an oral disc and a spiracle (both structures absent in *A. nidicola*); from *A. caeruleodactylus* by having five short papillae on each side of the lateral margin of the anterior labium (two papillae on each side); from *A. caldwellae*, *A. femoralis*, *A. hodli* and *A. velocicantus* by the presence of long papillae on the posterior labium (short papillae in all species); from *A. grillicantus* by having a “V”-shaped lower jaw sheath (arc-shaped); and from *A. grillisimilis* and *A. subfolionidificans* by having the labial tooth row P-3 shorter than P-1 and P-2 (P-1, P-2 and P-3 of similar length in all species). Tadpoles of *A. conspicuus*, *A. flaviventris*, *A. gasconi*, *A. pacaas*, *A. paleci*, *A. sieggreenae* and *A. trilineatus* are undescribed.

### Description of holotype

**Figure 3:**
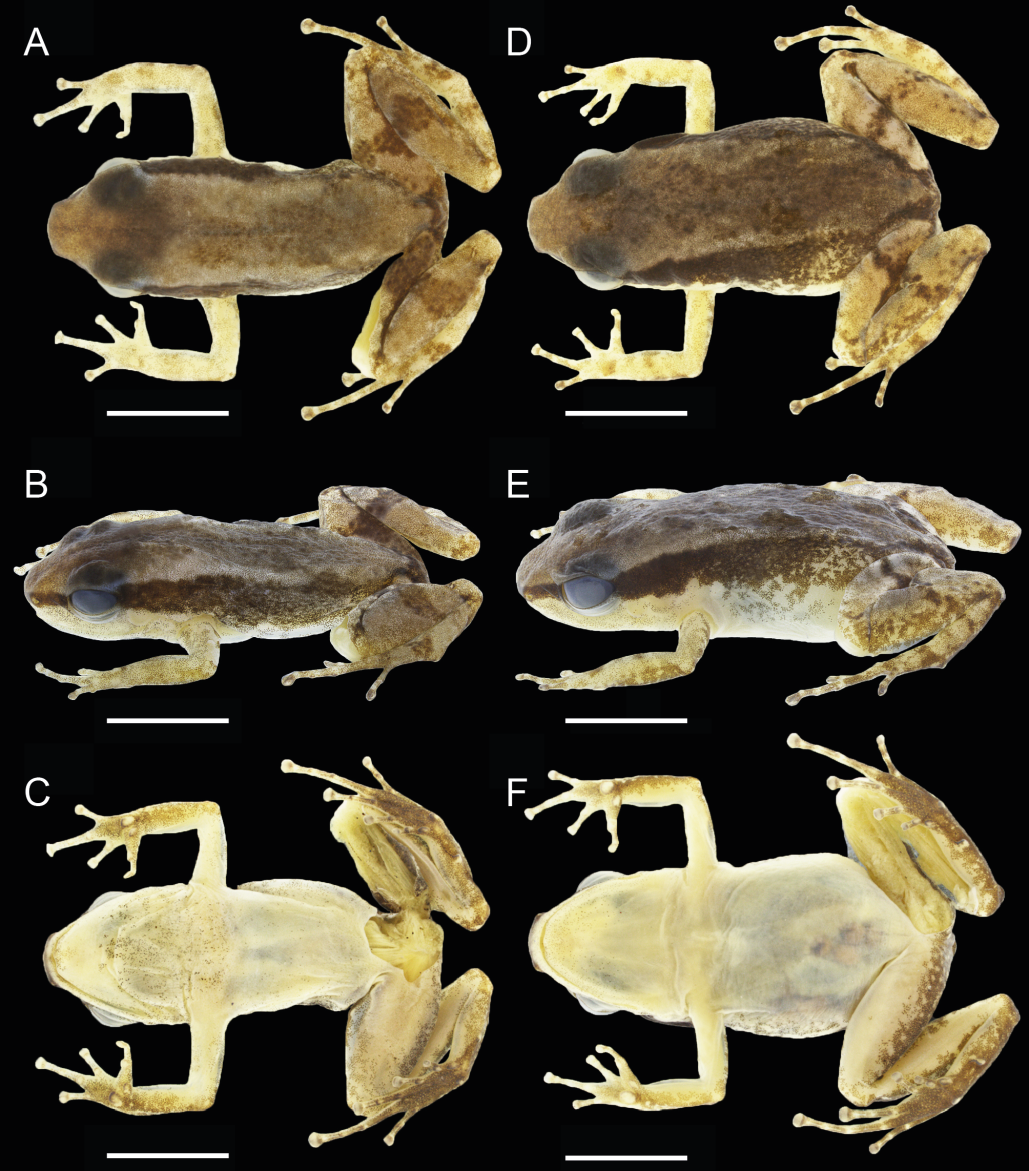
Dorsal, dorsolateral and ventral views of the male holotype, INPAH 42923 (A–C), and the female paratype, INPAH 42939 (D–F), of *Allobates kamilae* sp. nov. Scale bar: five mm.

**Figure 4:**
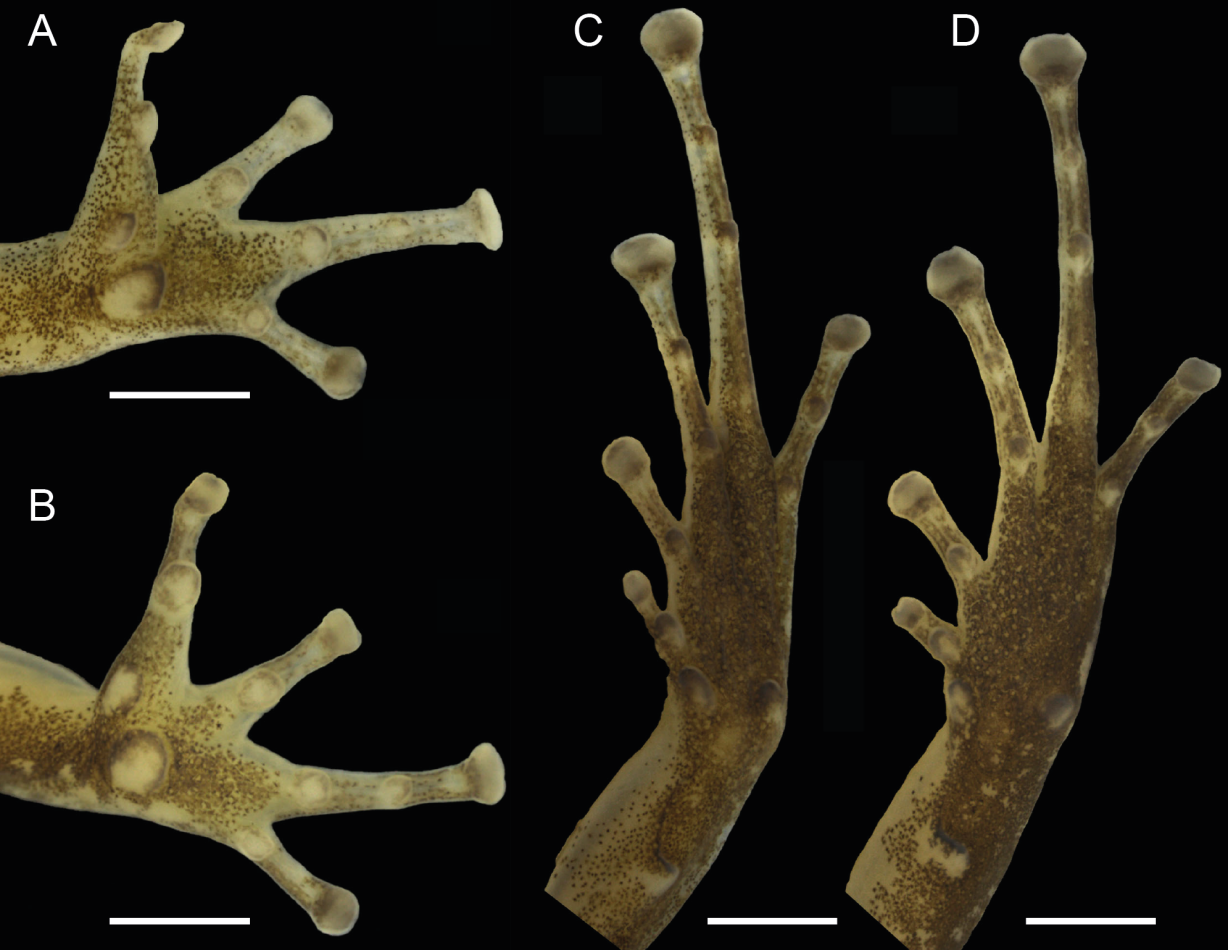
Ventral views of the hand and foot of the male holotype, INPAH 42923 (A, C), and the female paratype, INPAH 42939 (B, D), of *Allobates kamilae* sp. nov. Scale bar: one mm.

INPAH 42923 (field number APL 14840), an adult male (Figs. 3A–3C, 4A, 4C), SVL 16.0 mm; head slightly longer than wide (HW/HL = 0.98), head width 34% of SVL and head length 34% of SVL; snout truncate and rounded in dorsal and lateral views, respectively; snout length 42% of HL; internarial distance 39% of HW; canthus rostralis slightly convex in cross section; eye-nostril distance 69% of EL; nostril opening directed laterally; tympanum rounded, small, 44% of EL, tympanic annulus visible; tongue attached anteriorly, rounded posteriorly; median lingual process absent; vocal sac present, single, subgular.

Forearm slightly thicker and smaller than the upper arm (FAL/UAL = 0.91); forearm and upper arm lengths represent 20% and 22% of SVL, respectively; hand small, HANDIII 25% of SVL; finger II 0.25 mm longer than finger I; finger II 0.2 mm longer than finger IV; when appressed, finger IV does not reach the distal subarticular tubercle of finger III; finger II reaches the distal subarticular tubercle of finger III; relative length of fingers III >II >I = IV; finger III not swollen (Fig. 4A); lateral fringes and basal webbing absent on fingers; palmar tubercle elliptic, DPT 13% of HANDIII; thenar tubercle width 60% of DPT; two subarticular tubercles on finger III, a single subarticular tubercle on fingers I, II and IV (Fig. 4A); disc expanded on all fingers except finger I; width of disc on finger III 0.6 mm; width of third phalange of finger III 50% of WFD.

Hind limbs robust; thigh slightly longer than tibia (THL/TL = 1.01); thigh and tibia length 43% and 43% of SVL, respectively; foot length 99% of THL and equal to tibia length. Relative length of toes IV > III > V > II > I; basal webbing present between toes III and IV (Fig. 4C); tip of toe I reaches subarticular tubercle on toe II; tip of toe III reaches medial subarticular tubercle on toe IV; tip of toe V reaches mid-level of the proximal phalange of toe IV; disc on toe IV oval, slightly wider than disc on finger III (WTD/WFD = 1.08). Inner metatarsal tubercle oval and outer metatarsal tubercle rounded; median metatarsal tubercle absent (Fig. 4C). Metatarsal fold absent; tarsal keel in form of a curved fold, unconnected to inner metatarsal tubercle. A single subarticular tubercle on toes I and II, two subarticular tubercles on toes III and V, and three subarticular tubercles on toe IV; proximal tubercles poorly defined. Dorsum and shank smooth with scattered small tubercles; venter smooth (Figs. 3A–3C).

In preservative, dorsal surface of snout and head light brown; dorsum greyish brown with a faded brown hourglass-shaped mark; dorsal surface of arms and hands cream with several small, light brown blotches; dorsal surface of hindlimbs brownish cream with a transverse, irregular, dark-brown band on thigh, shank and tarsus. Pale dorsolateral stripe absent; dark brown lateral stripe from the snout to the cloacal region, with several small pale cream spots scattered on its central and inguinal portion; pale ventrolateral stripe absent.

### Intraspecific variation

**Figure 5:**
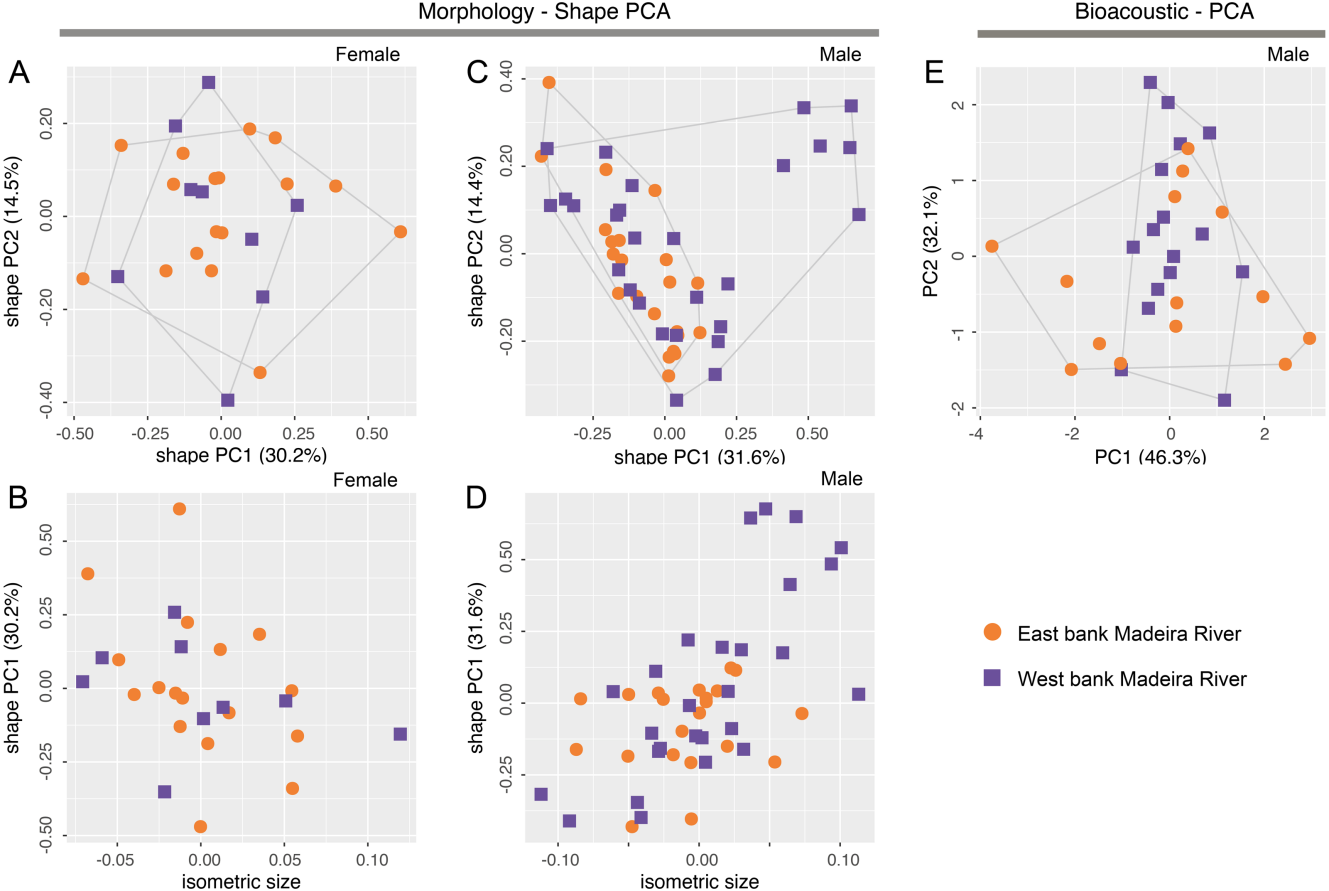
Shape (A–D) and standard (E) principal component analyses (PCA) of morphological and bioacoustic data of *Allobates kamilae* sp. nov. from east and west banks of the Madeira River, Brazilian Amazonia. Morphological analyses are based on 23 morphometric measurements; the bioacoustic analysis is based on four acoustic parameters.

Morphometric variables are summarized in Table 3. Body shape of male and female *Allobates kamilae* sp. nov. from opposite banks of the upper Madeira River overlap in the first two principal components of shape PCAs (Figs. 5A, 5B). Neither body shape (PC1 and PC2) nor isosize (Figs. 5C, 5D) of either sex differs statistically between banks (ANOVA *p* > 0.05 in shape PC1 and PC2). Similarly, multidimensional bioacoustic spaces of males from opposite banks overlap (Fig. 5E) and do not differ from one another (ANOVA *p* > 0.05 for PC1 and PC2). See Table S6 for variation and loadings of principal components from morphometric analyses and Table S7 for acoustic analyses.

**Table 3:** Morphometric measurements of the type series of *Allobates kamilae* sp. nov. and *Allobates tapajos*. Values depict mean ± standard error (range). Trait acronyms are defined in the text.

Traits	*Allobates kamilae* sp. nov.			*Allobates tapajos*	
	Females (*n* = 24)	Males (*n* = 49^*^)	Holotype	Females (*n* = 10)	Males (*n* = 14)
SVL	16.7 ± 0.7 (15.2–17.7)	15.8 ± 0.7 (14.5–17.4)	16.1	16.0 ± 0.5 (15.3–16.6)	15.0 ± 0.6 (14.0–16.1)
HL	5.7 ± 0.4 (5.1–6.7)	5.4 ± 0.4 (4.5–6.9)	5.5	4.9 ± 0.2 (4.5–5.1)	4.6 ± 0.2 (4.2–4.8)
HW	5.9 ± 0.4 (5.3–6.7)	5.5 ± 0.4 (4.4–6.4)	5.4	5.4 ± 0.1 (5.2–5.6)	5.2 ± 0.2 (5.0–5.6)
SL	2.3 ± 0.2 (2.0–2.7)	2.2 ± 0.3 (1.8–2.9)	2.3	2.4 ± 0.1 (2.3–2.7)	2.1 ± 0.2 (1.8–2.5)
EN	1.7 ± 0.1 (1.6–2.0)	1.6 ± 0.2 (1.2–2.3)	1.6	1.6 ± 0.1 (1.5–1.7)	1.4 ± 0.1 (1.3–1.6)
IN	2.3 ± 0.1 (2.0–2.5)	2.2 ± 0.1 (1.9–2.4)	2.1	2.4 ± 0.1 (2.3–2.5)	2.2 ± 0.1 (2.1–2.3)
EL	2.3 ± 0.1 (2.0–2.6)	2.3 ± 0.2 (1.8–2.9)	2.3	2.6 ± 0.1 (2.3–2.7)	2.4 ± 0.1 (2.2–2.7)
IO	4.7 ± 0.3 (3.9–5.0)	4.6 ± 0.2 (4.1–5.1)	4.6	4.8 ± 0.2 (4.3–4.9)	4.5 ± 0.1 (4.3–4.8)
TYM	1.0 ± 0.2 (0.6–1.4)	0.9 ± 0.2 (0.7–1.8)	1.0	1.0 ± 0.1 (0.8–1.2)	1.1 ± 0.1 (1.0–1.2)
FAL	3.3 ± 0.3 (2.8–3.7)	3.3 ± 0.2 (2.3–3.8)	3.2	3.6 ± 0.3 (3.1–3.9)	3.4 ± 0.2 (3.0–3.6)
UAL	3.5 ± 0.2 (3.0–3.9)	3.5 ± 0.4 (2.8–4.6)	3.5	4.1 ± 0.2 (4.0–4.5)	4.1 ± 0.3 (3.5–4.6)
HANDI	2.8 ± 0.2 (2.5–3.5)	2.8 ± 0.3 (2.1–3.7)	2.5	3.1 ± 0.1 (3.0–3.2)	3.0 ± 0.1 (2.7–3.2)
HANDII	2.7 ± 0.2 (2.4–3.3)	2.8 ± 0.2 (2.4–3.5)	2.8	2.8 ± 0.1 (2.7–3.0)	2.8 ± 0.1 (2.5–2.9)
HANDIII	3.9 ± 0.2 (3.4–4.3)	3.9 ± 0.3 (3.3–4.8)	4.0	3.9 ± 0.1 (3.6–4.1)	3.8 ± 0.2 (3.5–4.1)
HANDIV	2.6 ± 0.2 (2.1–2.9)	2.6 ± 0.2 (2.1–3.4)	2.6	2.7 ± 0.1 (2.5–2.9)	2.7 ± 0.1 (2.5–2.9)
WFD	0.5 ± 0.1 (0.5–0.7)	0.6 ± 0.1 (0.5–0.8)	0.6	1.2 ± 0.1 (1.1–1.3)	1.1 ± 0.1 (1.0–1.3)
TL	7.7 ± 0.4 (6.4–8.3)	7.5 ± 0.4 (6.5–8.3)	6.8	7.8 ± 0.2 (7.3–8.1)	7.5 ± 0.3 (6.6–8.0)
FL	6.9 ± 0.6 (5.0–8.0)	6.8 ± 0.4 (5.8–7.9)	6.8	7.0 ± 0.4 (6.5–7.6)	7.1 ± 0.3 (6.6–7.6)
THL	7.9 ± 0.4 (7.0–8.7)	7.6 ± 0.4 (6.4–8.4)	6.9	7.8 ± 0.2 (7.5–8.0)	7.4 ± 0.3 (6.6–7.8)
DPT	0.5 ± 0.1 (0.5–0.7)	0.5 ± 0.1 (0.4–0.7)	0.5	0.6 ± 0.1 (0.5–0.7)	0.6 ± 0.1 (0.5–0.7)
WTT	0.2 ± 0.0 (0.2–0.3)	0.2 ± 0.1 (0.2–0.4)	0.3	0.4 ± 0.1 (0.3–0.5)	0.4 ± 0.1 (0.3–0.5)
WTD	0.7 ± 0.1 (0.5–0.8)	0.7 ± 0.1 (0.6–0.9)	0.7	0.7 ± 0.1 (0.6–0.8)	0.7 ± 0.1 (0.6–0.8)
WPF	0.3 ± 0.0 (0.3–0.4)	0.3 ± 0.1 (0.2–0.4)	0.3	0.3 ± 0.0 (0.3–0.4)	0.4 ± 0.1 (0.3–0.5)

**Notes.**

Abbreviations n sample size

* An asterisk (*) includes the holotype.

*Allobates kamilae* sp. nov. exhibits sexual dimorphism in five morphological traits. Whereas females are larger than males on both banks of the Madeira River (SVL: *S*^2^ = 13.7, *F* = 28.33, *df* = 71, *p* = 0.0076), males are larger than females in FAL (*S*^2^ = 8.72, *F* = 11.97, *df* = 70, *p* = 0.0076), WFD (*S*^2^ = 9.41, *F* = 11.07, *df* = 71, *p* = 0.0076) and WPF (*S*^2^ = 10.96, *F* = 48.70, *df* = 64, *p* < 0.0001). Riverbank-sex interaction has a significant effect on DPT (*S*^2^ = 2.83, *F* = 4.95, *df* = 64, *p* = 0.0295): females are larger than males in DPT on the east bank, but males are larger in the west bank (*S*^2^ = 3.93, *F* = 6.88, *df* = 64, *p* = 0.0478).

**Figure 6:**
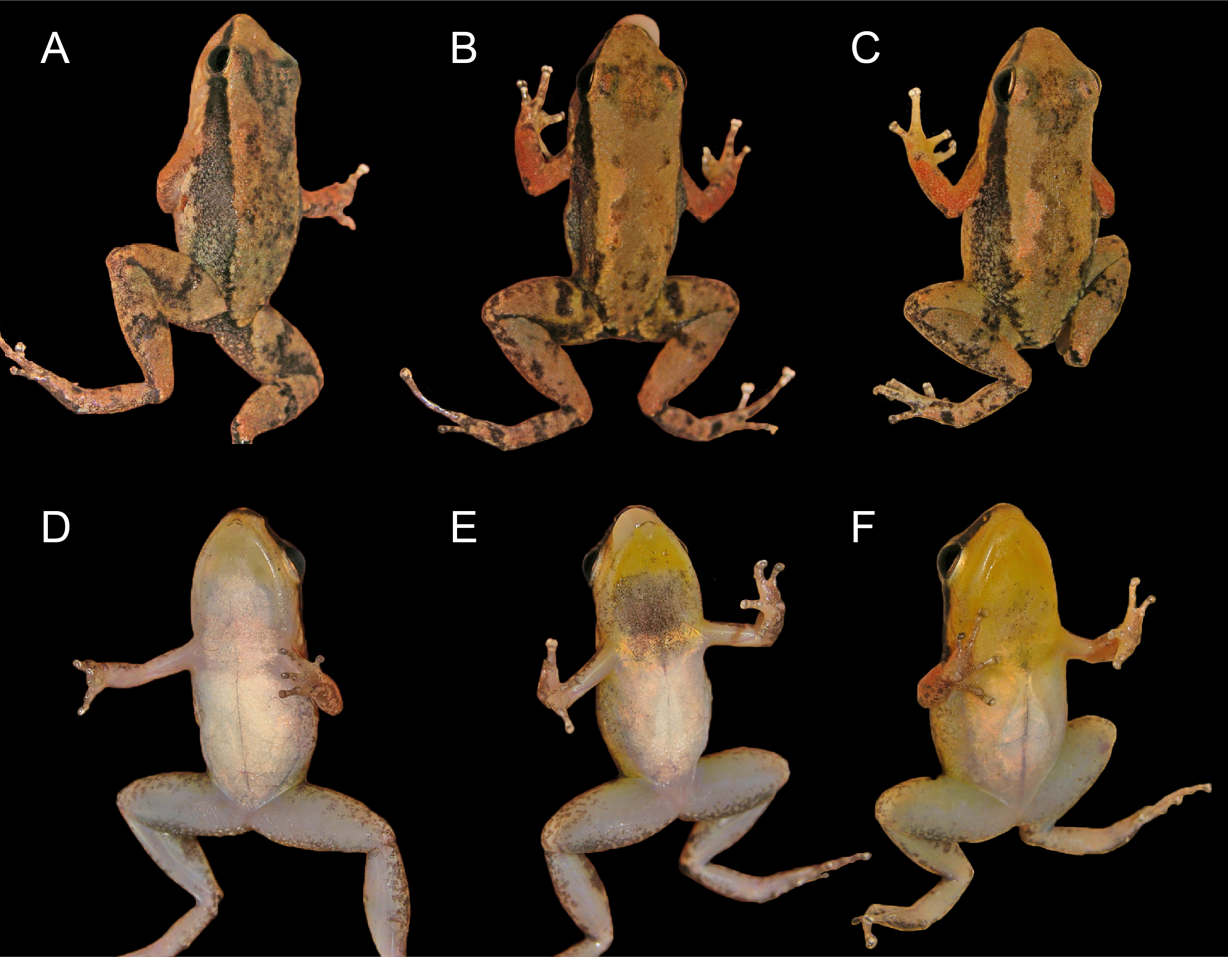
Color in life of adult *Allobates kamilae* sp. nov. (A, B) Female from the *Abunã Direito* sampling module, INPAH 42958, SVL = 16.1 mm. (C, D) Male from the *Abunã Direito* sampling module, INPAH 42932, SVL = 15.8 mm. (E, F) Male from the *Abunã Esquerdo* sampling module, INPAH 42881, SVL = 16.4 mm.

Coloration in life is described from photographs of two males and one female (Fig. 6). The dorsum is yellowish brown to light brown with a conspicuous or inconspicuous dark hourglass-shaped mark (cf. Figs. 6B, 6F and 6D); dorsal surface of arm reddish brown; dorsal surfaces of fingers and hindlimbs yellowish brown to light brown, with one diffuse dark brown band on thigh and shank; anterior surface of thigh light grey to yellowish brown with diffuse dark blotches, posterior surface mostly dark brown to dark grey; a half-moon-shaped mark on each side of the cloaca, colored cream to yellowish cream, is more conspicuous in some individuals than in others (cf. Figs. 6D and 6C, Fig. 6E). A dark lateral stripe from the tip of the snout to the inguinal region is diffuse posteriorly; oblique line absent; dorsolateral and ventrolateral stripes absent. Iris is golden with a horizontal black band centrally.

Calling males have a golden yellow throat and chest, with melanophores concentrated on the vocal sac and chest; belly is white centrally but tan to yellowish white peripherally (Figs. 6C, 6E). Females have a white throat with randomly scattered melanophores and edges of the jaw yellowish; chest is whitish tan, belly is white (Fig. 6A). In both sexes, ventral surfaces of the limbs are light grey to whitish grey, palmar surfaces of the hands are light brown, and plantar surfaces of the feet are dark brown.

In preservative, coloration of the type series generally resembles that of the holotype and illustrated paratype (Fig. 3). Dark dorsal marks on individuals collected in 2004 and 2005 are completely faded except on four individuals in which the hourglass-shaped mark is barely visible. The anterior portion of the lateral stripe remains dark in most old individuals but is completely faded on three of them. All specimens collected after 2010 show the hourglass-shaped dorsal mark, which is more conspicuous in some individuals than others.

### Description of tadpoles

The following morphological description is based on 12 tadpoles at Gosner stage 39 (lots INPAH 42851–53) from the *Jirau Direito* and *Morrinhos* sampling modules, east bank of the Madeira River. Table 4 summarizes 17 morphometric traits measured on 51 tadpoles, stages 34–40.

**Table 4:** Morphometric measurements of tadpoles of *Allobates kamilae* sp. nov. Gosner stages 34–40, from Jirau Direito (lots INPAH 42851 and 42853) and Morrinhos (lot INPAH 42852) sampling modules, upper Madeira River, municipality of Porto Velho, Rondônia, Brazil. Values depict mean ± standard error (range). Trait acronyms are defined in the text.

Traits	Stages 34–36 (*n* = 6)	Stage 37 (*n* = 10)	Stage 38 (*n* = 9)	Stage 39 (*n* = 12)	Stage 40 (*n* = 14)
BH	3.0 ± 0.4 (2.3–3.5)	3.3 ± 0.4 (2.3–3.7)	3.5 ± 0.4 (2.9–4.0)	3.8 ± 0.2 (3.5–4.1)	3.8 ± 0.3 (2.8–4.2)
BL	2.8 ± 0.8 (1.7–3.9)	2.4 ± 0.5 (2.0–3.5)	3.4 ± 0.7 (2.0–4.1)	3.7 ± 0.2 (3.5–4.0)	3.9 ± 0.3 (3.6–4.4)
BW	4.6 ± 0.4 (3.9–5.0)	4.9 ± 0.3 (4.3–5.5)	5.0 ± 0.3 (4.6–5.6)	4.8 ± 0.2 (4.4–5.2)	5.0 ± 0.4 (4.3–5.5)
ED	1.1 ± 0.1 (1.0–1.2)	1.2 ± 0.1 (1.0–1.3)	1.2 ± 0.1 (1.1–1.3)	1.2 ± 0.1 (1.1–1.4)	1.3 ± 0.1 (1.1–1.4)
END	0.4 ± 0.1 (0.3–0.5)	0.4 ± 0.1 (0.3–0.6)	0.5 ± 0.1 (0.4–0.6)	0.5 ± 0.1 (0.3–0.7)	0.6 ± 0.1 (0.4–0.7)
HWLE	3.8 ± 0.6 (3.0–4.5)	4.1 ± 0.5 (3.0–4.8)	4.8 ± 0.5 (4.1–5.6)	4.7 ± 0.2 (4.2–5.0)	5.0 ± 0.4 (4.1–5.6)
IND	0.9 ± 0.1 (0.8–1.0)	0.9 ± 0.1 (0.8–1.1)	0.9 ± 0.1 (0.8–1.0)	1.0 ± 0.1 (0.8–1.1)	1.0 ± 0.1 (0.9–1.1)
IOD	1.1 ± 0.1 (0.9–1.2)	1.1 ± 0.1 (1.0–1.2)	1.1 ± 0.1 (1.0–1.2)	1.1 ± 0.1 (1.0–1.3)	1.2 ± 0.1 (1.1–1.3)
MTH	2.7 ± 0.3 (2.2–3.0)	2.7 ± 0.3 (2.3–3.0)	2.8 ± 0.3 (2.3–3.3)	2.9 ± 0.3 (2.4–3.2)	2.9 ± 0.5 (1.5–3.4)
NSD	0.4 ± 0.1 (0.3–0.5)	0.5 ± 0.2 (0.3–0.7)	0.5 ± 0.1 (0.4–0.7)	0.5 ± 0.1 (0.3–0.7)	0.5 ± 0.1 (0.4–0.7)
ODW	1.4 ± 0.2 (1.1–1.5)	1.3 ± 0.2 (1.0–1.7)	1.5 ± 0.3 (1.1–2.0)	1.7 ± 0.3 (1.1–2.0)	1.7 ± 0.2 (1.5–2.0)
SLT	1.5 ± 0.2 (1.3–1.8)	1.4 ± 0.3 (1.0–1.8)	1.4 ± 0.2 (1.0–1.6)	1.3 ± 0.2 (1.0–1.5)	1.2 ± 0.2 (1.0–1.5)
TAL	12.6 ± 0.8 (11.3–13.6)	13.0 ± 1.6 (9.4–14.4)	14.4 ± 0.9 (12.5–15.5)	14.7 ± 0.9 (12.8–15.6)	14.8 ± 0.6 (13.8–15.8)
TL	18.8 ± 1.3 (16.7–20.0)	19.3 ± 1.4 (16.0–20.5)	20.3 ± 0.4 (19.8–21.2)	20.2 ± 0.1 (20.0–20.4)	20.3 ± 0.1 (20.1–20.5)
TMH	1.9 ± 0.1 (1.7–2.0)	2.0 ± 0.1 (1.8–2.2)	2.0 ± 0.2 (1.8–2.3)	2.0 ± 0.1 (1.9–2.3)	2.1 ± 0.2 (1.8–2.3)
TMW	2.0 ± 0.2 (1.6–2.3)	2.0 ± 0.1 (1.9–2.2)	2.2 ± 0.2 (2.0–2.5)	2.3 ± 0.2 (2.1–2.6)	2.4 ± 0.2 (2.2–2.7)
VTL	1.2 ± 0.1 (1.0–1.4)	1.2 ± 0.2 (0.9–1.5)	1.2 ± 0.2 (0.8–1.4)	1.2 ± 0.2 (1.0–1.5)	1.2 ± 0.2 (0.8–1.5)

**Notes.**

Abbreviations n sample size

**Figure 7:**
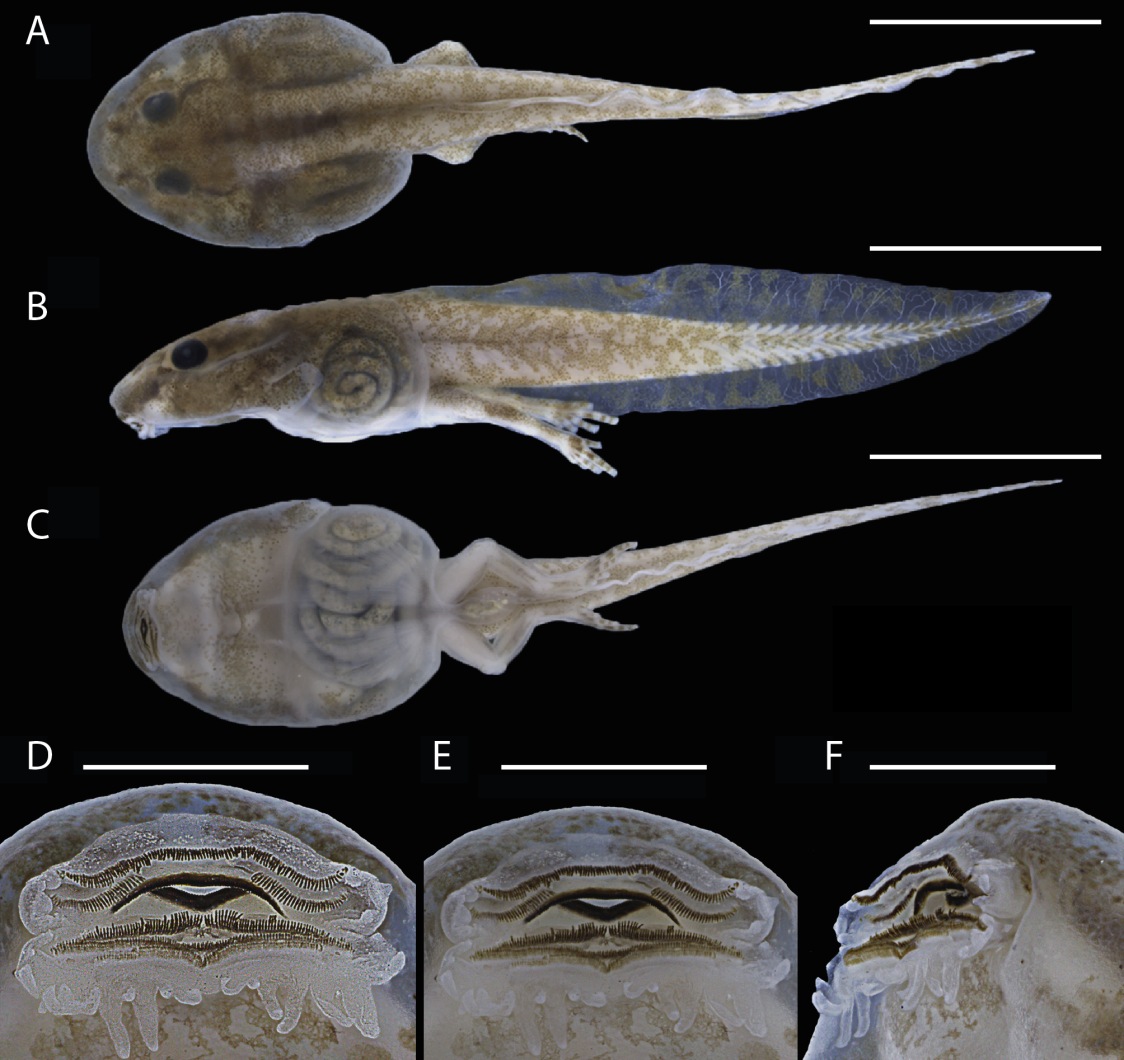
Preserved tadpole of *Allobates kamilae* sp. nov. (lot INPAH 42851), Gosner stage 39, from the *Jirau Direito* sampling module. (A) Dorsal, (B) lateral and (C) ventral views of the body; (D, E) ventral and (F) lateroventral views of the oral disc. Scale bar: A–C, five mm; D–F, one mm.

Body ovoid in dorsal view, rounded anteriorly but truncate posteriorly (Figs. 7A–7C) and longer than wide (BL/BW = 115% ± 18), but flattened in lateral view (BH/BL = 69% ± 9). Body length 27% ± 4 of total length; head width 98% ± 5 of body width. Snout rounded in dorsal view but slightly pointed in lateral view; snout length shorter than eye diameter (NSD/ED = 43% ± 11). Large eyes positioned dorsally and directed laterally; eye diameter 231%  ± 32 of END; interorbital distance 117% ± 16 of IND and 24% ± 2 of HWEL. Small nares located dorsally and directed anterolaterally, visible in dorsal and lateral views; internarial distance 21% ± 3 of HWEL. Spiracle sinistral, visible in dorsal, lateral and ventral views, and directed transversely; inner wall free from body; tube length 27% ± 5 of HWEL. Gut coiled and visible through the skin to the naked eye; its axis is directed to the left side of the body. Vent tube dextral, right wall displaced dorsally; tube length 92% ± 23 of spiracle. Tail length 73% ± 4 of total length; tail maximum height 77% ± 9 of BH; tail muscle robust, tail muscle maximum height 70% ± 7 of MTH. Dorsal fin emerges at the thigh-body insertion and reaches its maximum depth on the central portion of the tail; upper fin deeper than the lower one along the first two thirds of the tail. Tail tip acuminate and lacks a flagellum.

Oral disc is located anteroventrally and is emarginated laterally (Figs 7D, 7E); oral disc width 35%  ± 6 of body width. Anterior labium with three or four short pyramidal papillae on each lateral margin. Posterior labium with a single marginal row of pyramidal papillae of variable length; short papillae are located close to the labium angle and on the central portion of the labium, all other papillae are elongated (Figs. 7D, 7E); elongate papillae are three times longer than shorter ones. Submarginal papillae absent. Upper jaw sheath arch-shaped; lower jaw sheath “V”-shaped, deeper than upper one; cutting edge of each jaw sheath is serrated along its entire length. Labial keratodont row formula (LKRF) 2(2)/3(1); tooth row A-1 complete; tooth row A-2 interrupted by a medial gap of ∼0.5mm, each segment ∼0.4 mm long; tooth row P-1 interrupted by a small medial gap; P-1 and P-2 of similar length (1.13 mm) but shorter than P-3 (1.25 mm).

**Figure 8:**
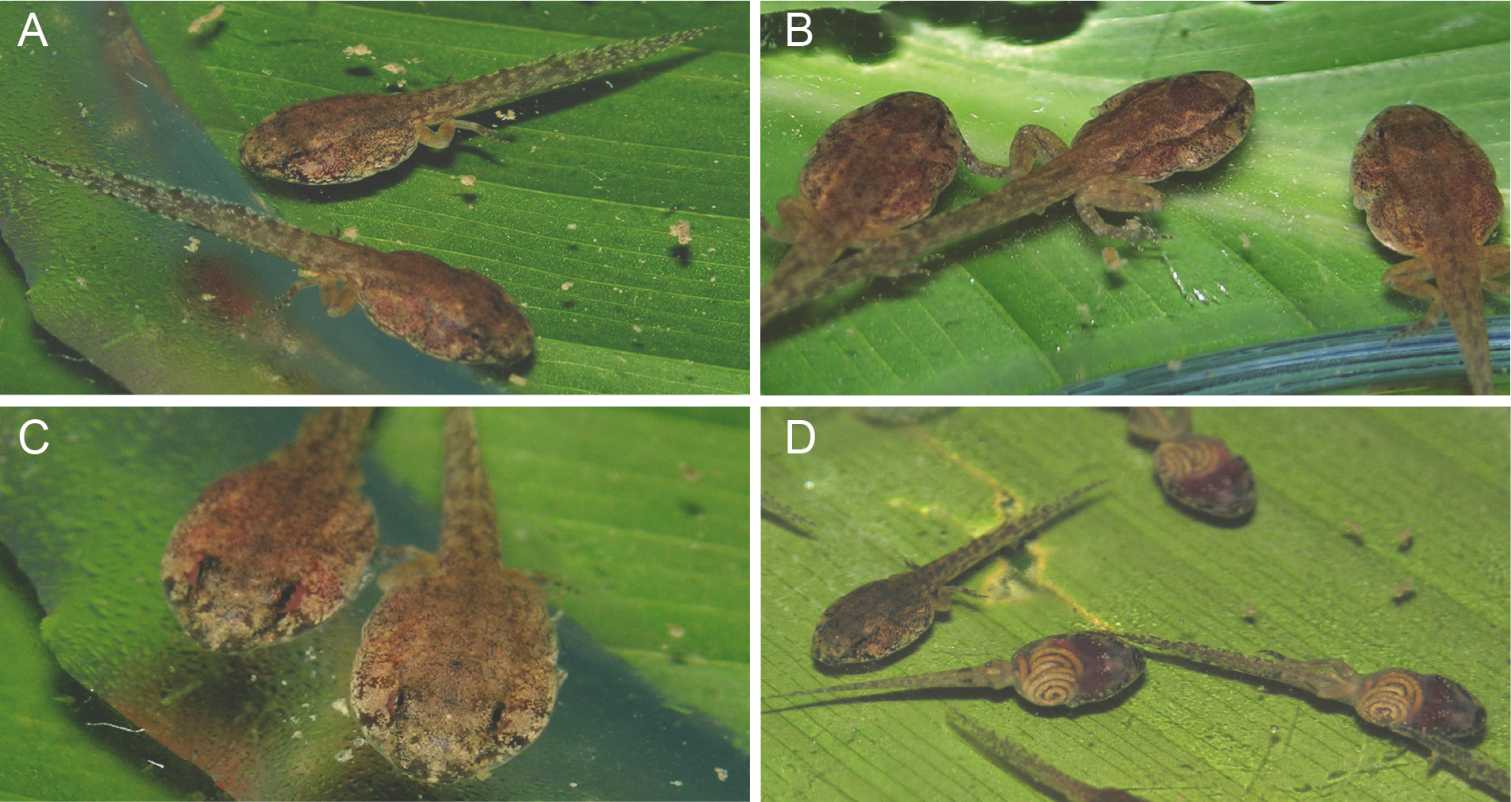
Live tadpoles (lot INPAH 42851) of *Allobates kamilae* sp. nov., Gosner stages 39–40, from the *Jirau Direito* sampling module.

In life, dorsal skin of head and body cream to brownish cream, covered with dark melanophores (Fig. 8). A brown hourglass-shaped mark covers the dorsum of some tadpoles; a thin dark brown dorsolateral stripe from the tip of the snout to the inguinal region is present in some specimens. Dark brown blotches on the latero-frontal portion of the head. Iris metallic cream with a horizontal dark bar. Tail muscle cream and fins translucent with brown to dark brown blotches; white dots mostly on anterior portion of the tail. Ventral surfaces translucent, internal organs visible through the skin; brown reticular-shaped blotches surround the posterior labium; dark melanophores and white dots randomly distributed on posterior portion of ventral surface of the head.

Coloration in preservative is similar to that in life (Figs. 7 and 8). Cream-colored surfaces become whitish cream, and brown marks become faded or greyish brown. White dots on the tail and on the posterior portion of the head disappear.

### Vocalization

**Figure 9:**
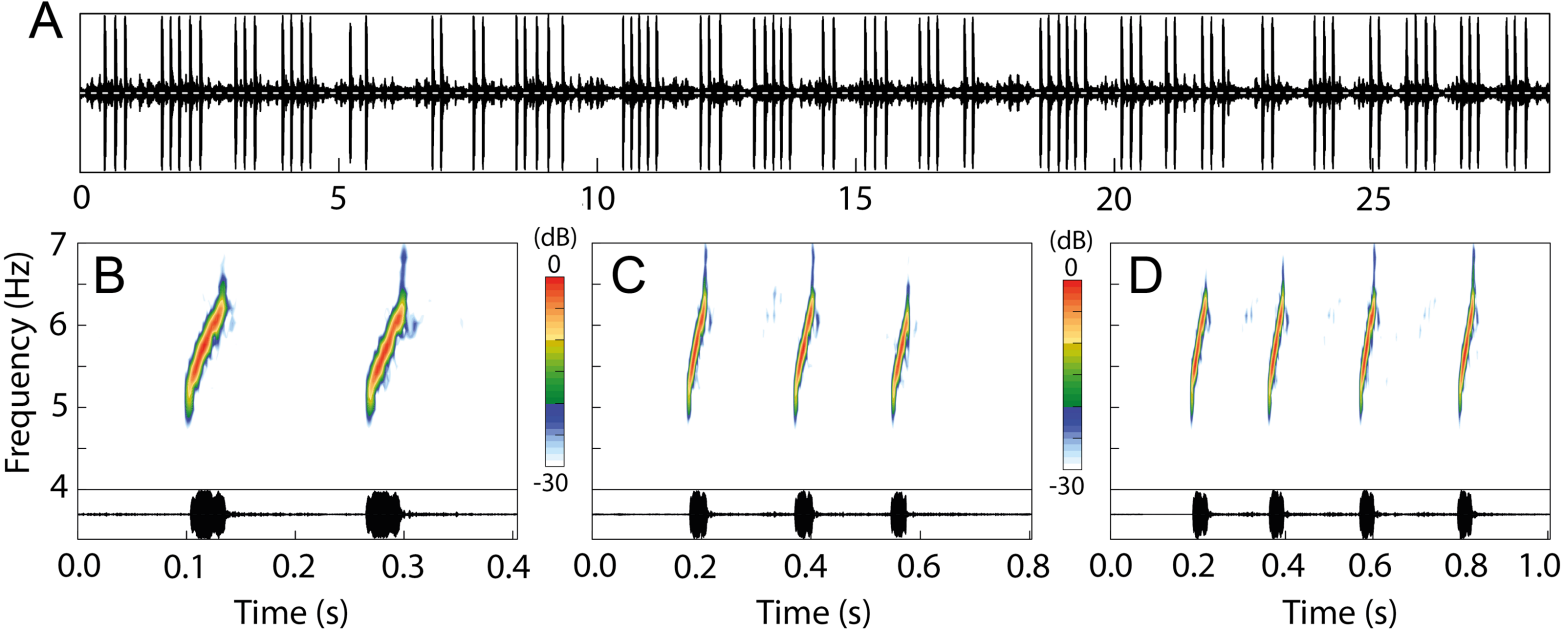
Advertisement call of *Allobates kamilae.* sp. nov. from the upper Madeira River. (A) Oscillogram showing the continuous emission of 25 calls with variable note arrangements. Spectrograms and oscillograms of the three most common note arrangements: (B) two-, (C) three- and (D) four-note calls. Recorded males: (A) INPAH 42923, air temperature 25.7 ° C, *Teotônio* sampling module; (B–D) INPAH 42882, air temperature 25 °C, *Abunã Esquerdo* sampling module.

The advertisement call of *Allobates kamilae* sp. nov. comprises continuously emitted groups of notes arranged mostly in pairs, trios or quartets (Fig. 9; Table 2), and rarely as single notes or groups that contain up to 14 notes (Table S8). The following summary describes the most common arrangements (2–4-note calls). Two-note calls have a duration of 211 ± 35 ms (141–333 ms), while three-note and four-note calls have a duration of 376 ± 60 ms (260–593 ms) and 527 ± 73 ms (387–794 ms), respectively. Inter-call intervals are similar among 2–4-note calls and range from 299 to 2,236 ms. Notes are tonal with an ascending frequency modulation (Fig. 9B). Note duration is also similar among the most frequent arrangements and ranges from 18 to 49 ms, as are inter-note intervals, which last from 85 to 268 ms (Table 2). The dominant frequency of calls ranges from 5,211 to 6,201 Hz.

### Distribution and natural history notes

*Allobates kamilae* sp. nov. is known from four localities on the west bank of the upper Madeira River and three localities on the east bank, close to the municipality of Porto Velho, state of Rondônia, Brazil. A single specimen (HJ 285) reported by Vacher et al. (2020) as collected from the Jirau Dam is tentatively attributed by us to *A. kamilae* sp. nov. based on molecular data. However, geographic coordinates provided by Vacher et al. (2020) for this specimen put its locality within an unforested and developed area in the heart of Porto Velho City, along the east bank of Madeira River. The K2P genetic distance between HJ 285 and other specimens collected by us suggests that this specimen did come from the east bank population, but its reported sampling locality is likely incorrect: Porto Velho City is about 136 km from the Jirau Dam, and this large urban area is not capable of maintaining a population of this species.

*Allobates kamilae* sp. nov. inhabits the leaf litter of riparian forests of the main course of the upper Madeira River. Despite an extensive sampling effort in plots located up to 5 km inland from the river, *A. kamilae* sp. nov. has been recorded only within 500 m of each riverbank. Males and females are active between dawn and dusk. Breeding coincides with the rainy season (November to April). Males call mostly from 0530 to 0830 h and from 1630 h to dusk, and rarely during the day. Breeding males are likely territorial; they respond to advertisement call playback and attack speakers placed close to them. However, territory size remains unknown.

**Figure 10:**
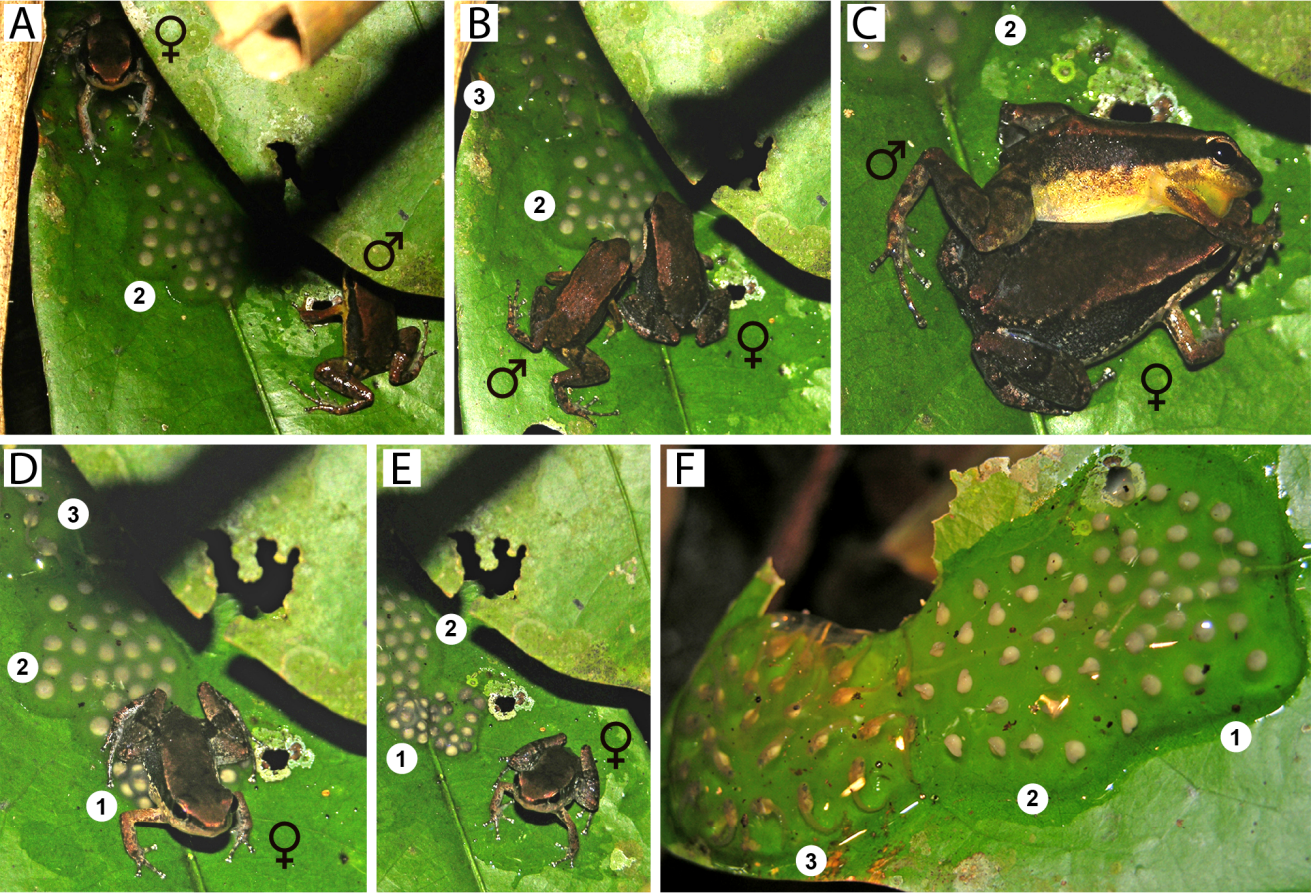
*Allobates kamilae* sp. nov. mating in the *Abunã Esquerdo* sampling module. (A) Female climbing over a previously deposited clutch to approach the male. (B) Male approaching the female to embrace her in a lateral cephalic amplexus (C). (D) Female depositing eggs. (E) Female moments before she left the egg deposition site. (F) The freshly deposited clutch has merged with a previously deposited clutch one day after deposition. Male (♂), INPAH 42944, SVL 16.9 mm; female (♀), uncollected. Numbers denote the temporal sequence of clutch deposition from newest (1) to oldest (3).

On the west bank, *Allobates kamilae* sp. nov. deposits eggs on adaxial surfaces of green leaves of small shrubs (*n* = 3), while on the east bank eggs are deposited inside rolled dried leaves on the forest floor (*n* = 4). Unlike most Amazonian *Allobates*, more than one clutch of a given male can be found on a single leaf (Fig. 10). Two male territories were found in the *Abunã Direito* sampling module, with one nest in each territory. The first nest had three clutches with 24 freshly laid eggs, 23 embryos and 26 tadpoles. Similarly, the second nest had three clutches with 20, 19 and 28 eggs. Eggs are enclosed initially within transparent jelly (Fig. 10), which becomes slightly cloudy over subsequent days. Although adults have not been observed transporting tadpoles to any body of water, we hypothesize that tadpoles complete their development in shallow waters of the Madeira River. In January 2019, several breeding individuals and clutches were found in an area with no puddles next to the east bank of the Madeira River.

Mating behavior was observed in early morning at the *Abunã Esquerdo* sampling module in December 2004 (Fig. 10). An adult male was calling while perched ∼20 cm above the ground on the adaxial surface of a live leaf of a small shrub. A female approached the shrub around 0730. The male led the female with courtship calls to a nearby shrub, where a live leaf was being used as an egg deposition site; it already held two other clutches. The male positioned himself on an unoccupied area on the leaf extremity and continued to call; the female jumped over the existing clutches to access the area close to the male (Fig. 10A). The male then approached the female (Fig. 10B) and embraced her in a lateral cephalic amplexus five times over the next 40 min (Fig. 10C). Following the last amplexus, the female began depositing eggs (*n* = 25) beside the existing clutches (Fig. 10D), whereupon the male jumped to the first shrub and after 5 min started calling again. The female stopped ovipositing after ∼10 min (Fig. 10E) and left the clutch. The next day, jelly of the freshly deposited clutch had swollen to such an extent that it was now connected to the nearest previously deposited clutch (Fig. 10F).

## Discussion

Cryptic diversity within *Allobates tapajos* was revealed initially by Vacher et al. (2020), who delimited five species through the automatic barcode gap discovery algorithm (ABGD; Vacher et al., 2020, Appendix S1). Based on the consensus among three molecular delimitation approaches (ABGD, GMYC and mPTP) and using a similar molecular database, Réjaud et al. (2020) delimited only four species, lumping *A*. *tapajos* clades C and E (*A. kamilae*). Subsequently, Silva et al. (2022) delimited five species within *A. tapajos* (GMYC and PTP) by adding a new lineage from southeastern Amazonia (clade F; Fig. 1) to those delimited previously by Vacher et al. (2020). Consensus among molecular species delimitation approaches used in the present study (ASAP, GMYC and mPTP) suggests that *A. tapajos* comprises six species (clades A–F). Molecular (Fig. 1) and bioacoustic (Table 2) evidence does not support the lumping of *A*. *tapajos* clades C and E (*A. kamilae*) by Réjaud et al. (2020).

*Allobates tapajos* clade F was recently reported by Silva et al. (2022) from dense ombrophilous forests of the Teles-Pires River, southeastern Brazilian Amazonia. Acoustic parameters of this candidate species overlap with those of *A. tapajos* sensu stricto (clade A), which could hamper its formal description as a distinct species. Indeed, the call structures of the two clades share a similar note arrangement, and temporal parameters overlap when distinct call arrangements (*e.g.*, two- and three-note calls) are summarized together in descriptive analyses. However, their calls differ in other temporal parameters when each arrangement is summarized separately (Table 2). As subtle acoustic differentiation within a given species complex is common in *Allobates* (Lima, Ferrão & Silva, 2020; Moraes & Lima, 2021; present study), detailed comparisons of acoustic traits are strongly recommended for species delimitation and diagnosis. Moreover, presenting detailed acoustic data or otherwise making it available through supplementary material (*e.g.*, Silva et al., 2022) or public repositories represents a best-practice that will enhance further taxonomic studies of other species complexes within *Allobates* and other anurans.

*Allobates kamilae* appears to be restricted geographically to riparian forests of the upper Madeira River (UMR); its known range falls entirely within the influence zone of the Santo Antônio and Jirau dams, the two largest dams constructed in the Madeira Basin. Recently, Dayrell et al. (2021) showed detrimental impacts of reservoir filling on *A. kamilae* (as *Allobates* sp. 1). Before reservoir filling behind the Santo Antônio dam, the species was commonly recorded in several plots near riverbanks, but four years later it was found in only one plot (Dayrell et al., 2021, Fig. 3). In January 2019, seven years after reservoir filling, we unexpectedly found a population of *A. kamilae* breeding in a very disturbed secondary forest on what is the new east bank, 5 km from its former location. Based on this observation and the species’ likely ability to respond to natural environmental changes in its habitat associated with the annual riverine flood pulse, *A. kamilae* may have colonized additional areas along newly formed riverbanks. However, both its current geographic distribution and its conservation status need to be assessed through widescale, long-term study.

*Allobates kamilae* is the second species described from the *A. tapajos* species complex and the fourth *Allobates* described from the upper Madeira Basin in recent years. With nine species, the upper Madeira Basin near the municipality of Porto Velho harbors the richest assemblage of *Allobates* reported in Brazilian Amazonia (Simões, Lima & Farias, 2010; Dias-Terceiro et al., 2015; Lima, Ferrão & Silva, 2020; present study). Seven of the species occur in the area with *A. kamilae*: four species exclusively on the west bank (*A.* aff. *gasconi*, *A. hodli*, *A. nidicola* and *A. tinae*), two others on the east bank (*A. flaviventris* and *A.* aff. *tinae*) and *A. femoralis* on both banks. The remaining three candidate species (*A.* aff. *gasconi*, *A.* aff. *tinae* and *A. tapajos* clade C) await formal description. These candidate species, together with other recently described species (Ferrão et al., 2017; Ferrão et al., 2020a; Ferrão et al., 2020b; Fouquet et al., 2021a; Fouquet et al., 2021b; Melo-Sampaio, Ferrão & Moraes, 2021; Ferrão et al., 2022), highlight both the importance of the upper Madeira Basin as a center of regional biodiversity and the need for additional taxonomic studies in this threatened part of Amazonia.

## Conclusions

The *Allobates tapajos* species complex comprises six species distributed in southwestern, southeastern, eastern and northeastern Amazonia. Yet, sampling remote and poorly explored areas will likely reveal additional candidate species (*e.g.*, Silva et al., 2022). Although *A. kamilae* resembles *A. tapajos* sensu stricto in morphology, these species nevertheless are easily diagnosed. Acoustic differentiation between these species, however, is achieved mainly through detailed comparisons of temporal traits within the same call arrangement. Recognition of additional candidate species in the *A. tapajos* species complex—as well as other species complexes in the genus—will likely require such fine-scale analyses. *Allobates kamilae* is known only from riparian forests in the impact zone of two large dams in the upper Madeira River, underscoring the urgent need for comprehensive assessments of its geographic distribution and conservation status.

## Supplemental Information

10.7717/peerj.13751/supp-1 Supplemental Information 1 Table S1–S8 and Figure S1

## Appendix

***Allobates caeruleodactylus***. Adults. Brazil: Amazonas: km 12 on the road to Autazes (INPAH 7238 [holotype]; 7229–32, 7234–37 [paratypes]). Tadpoles. Brazil: Amazonas: km 12 on the road to Autazes (lots INPAH 8037–46, 8085).

***Allobates caldwellae***. Adults. Brazil: Amazonas: Careiro, RAPELD M1 at km 32 of Brazilian federal highway BR-319 (INPAH 41047 [holotype]; 41045–46, 41049–53, 41055–57, 41059, 41061, 41063, 41067–69 [paratopotypes]).

***Allobates femoralis***. Adults. Brazil: Pará: Treviso (INPAH 11657–71, 15232, 30769–78); Itaituba (INPAH 26342–54).

***Allobates gasconi***. Adults. Brazil: Amazonas: Jainu, west bank of Juruá River (INPAH 3082 [holotype]; 3073, 3079, 3085, 3090, 3150–51, 3172, 3249, 3406, 3415, 3483–84, 3491, 3494, 3496, 3512–13 [paratypes]).

***Allobates grillicantus***. Adults. Brazil: Pará: Trairão (MPEG 43046 [holotype]; 43038–41, 43045, 43047–50 [paratopotypes]); km 24 on Brazilian federal highway BR-163 (INPAH 41352–56 [paratypes]). Tadpoles. Brazil: Pará: Trairão (lots MPEG 43051–53, INPAH 41357).

***Allobates grillisimilis***. Adults. Brazil: Amazonas: Borba (INPAH 30779 [holotype]; 30780–30808 [paratopotypes]); Nova Olinda do Norte (INPAH 30809–23 [paratypes]).

***Allobates hodli***. Adults. Brazil: Acre: Fazenda Experimental Catuaba (INPAH 11621–40 [paratypes]); Rondônia: Cachoeira do Jirau (INPAH 16555 [holotype]; 16541–54, 16556–69 [paratopotypes]), near Mutum-Paraná (INPAH 16596, 16730, 16739, 16756, 16758, 16767, 16771, 16777–78, 16788, 16805, 16818–19 [paratypes]).

***Allobates nidicola***. Adults. Brazil: Amazonas: km 12 on road to Autazes (INPAH 8093 [holotype]; 7253–59, 7261–62, 8094 [paratypes]; 28122, 28124, 28127, 28129, 28131, 28144, 28159, 28163, 28166, 28169, 28171–72, 28174, 28179, 28184–85 [topotypes]). Tadpoles. Brazil: Amazonas: km 12 on road to Autazes (INPAH 8021–33, 8137–39).

***Allobates tapajos*** sensu stricto (clade A). Adults. Brazil: Pará: Parque Nacional da Amazônia (INPAH 34425 [holotype]; 34402–24 [paratypes]). Tadpoles. Brazil: Pará: Parque Nacional da Amazônia (Lots INPAH 34426–27).

***Allobates tapajos*** sensu lato (clade C). Adults. Brazil: Rondônia: Porto Velho: Jací-Paraná-River (APL 19408, 20015, 20042).

***Allobates tinae***. Adults. Brazil: Rondônia: Porto Velho, west bank of upper Madeira River (INPAH 41012–21, 41029–36, 41041–44); Amazonas: Boca do Acre (INPAH 40976, 41022, 41027, 41037, 41040).

***Allobates velocicantus***. Adults. Brazil: Acre: Mâncio Lima (INPAH 41342 [holotype]; 41338–41, 41343–47, 41349 [paratopotypes]), Cruzeiro do Sul (INPAH 41350 [paratype]). Tadpoles. Brazil: Acre: Mâncio Lima (lot INPAH 41347).

## Additional Information and Declarations

### Field Study Permissions

The following information was supplied relating to field study approvals (i.e., approving body and any reference numbers): The Instituto Brasileiro do Meio Ambiente e dos Recursos Naturais Renováveis –IBAMA (Ministry of Environment, Government of Brazil) approved the field permit (permit numbers 1337-1 and 02001.000508/2008-99).

### DNA Deposition

The following information was supplied regarding the deposition of DNA sequences: The sequences are available at GenBank: MZ667618 to MZ667633.

### Data Availability

The following information was supplied regarding data availability: The raw morphometric and bioacoustic data of adults used in morphometric analyses, raw morphometric data of tadpoles, raw bioacoustic data of the new species used in intraspecific bioacoustic analyses, and raw bioacoustic data of the new species and its sister species used in interspecific bioacoustic analyses are available in the Supplemental Files. The call recordings are available at The Audiovisual Collection of the Museum of Biological Diversity (MDBio), Fonoteca Neotropical Jacques Vielliard (FNJV), UNICAMP (https://www2.ib.unicamp.br/fnjv/): - FNJV 50556 - https://www2.ib.unicamp.br/fnjv/collection.php?fnjv=50556. - FNJV 50557 - https://www2.ib.unicamp.br/fnjv/collection.php?fnjv=50557
- FNJV 50558 - https://www2.ib.unicamp.br/fnjv/collection.php?fnjv=50558
- FNJV 50559 - https://www2.ib.unicamp.br/fnjv/collection.php?fnjv=50559
- FNJV 50560 - https://www2.ib.unicamp.br/fnjv/collection.php?fnjv=50560
- FNJV 50561 - https://www2.ib.unicamp.br/fnjv/collection.php?fnjv=50561
- FNJV 50562 - https://www2.ib.unicamp.br/fnjv/collection.php?fnjv=50562
- FNJV 50563 - https://www2.ib.unicamp.br/fnjv/collection.php?fnjv=50563
- FNJV 50564 - https://www2.ib.unicamp.br/fnjv/collection.php?fnjv=50564
- FNJV 50565 - https://www2.ib.unicamp.br/fnjv/collection.php?fnjv=50565
- FNJV 50566 - https://www2.ib.unicamp.br/fnjv/collection.php?fnjv=50566
- FNJV 50567 - https://www2.ib.unicamp.br/fnjv/collection.php?fnjv=50567
- FNJV 50568 - https://www2.ib.unicamp.br/fnjv/collection.php?fnjv=50568
- FNJV 50569 - https://www2.ib.unicamp.br/fnjv/collection.php?fnjv=50569
- FNJV 50570 - https://www2.ib.unicamp.br/fnjv/collection.php?fnjv=50570
- FNJV 50571 - https://www2.ib.unicamp.br/fnjv/collection.php?fnjv=50571
- FNJV 50572 - https://www2.ib.unicamp.br/fnjv/collection.php?fnjv=50572
- FNJV 50573 - https://www2.ib.unicamp.br/fnjv/collection.php?fnjv=50573
- FNJV 50574 - https://www2.ib.unicamp.br/fnjv/collection.php?fnjv=50574
- FNJV 50575 - https://www2.ib.unicamp.br/fnjv/collection.php?fnjv=50575
- FNJV 50576 - https://www2.ib.unicamp.br/fnjv/collection.php?fnjv=50576
- FNJV 50577 - https://www2.ib.unicamp.br/fnjv/collection.php?fnjv=50577
- FNJV 50578 - https://www2.ib.unicamp.br/fnjv/collection.php?fnjv=50578
- FNJV 50579 - https://www2.ib.unicamp.br/fnjv/collection.php?fnjv=50579
- FNJV 50580 - https://www2.ib.unicamp.br/fnjv/collection.php?fnjv=50580
- FNJV 50581 - https://www2.ib.unicamp.br/fnjv/collection.php?fnjv=50581
- FNJV 50582 - https://www2.ib.unicamp.br/fnjv/collection.php?fnjv=50582
- FNJV 50583 - https://www2.ib.unicamp.br/fnjv/collection.php?fnjv=50583
- FNJV 50584 - https://www2.ib.unicamp.br/fnjv/collection.php?fnjv=50584
- FNJV 50585 - https://www2.ib.unicamp.br/fnjv/collection.php?fnjv=50585
- FNJV 50586 - https://www2.ib.unicamp.br/fnjv/collection.php?fnjv=50586
- FNJV 50587 - https://www2.ib.unicamp.br/fnjv/collection.php?fnjv=50587
- FNJV 50588 - https://www2.ib.unicamp.br/fnjv/collection.php?fnjv=50588
- FNJV 50589 - https://www2.ib.unicamp.br/fnjv/collection.php?fnjv=50589
- FNJV 50590 - https://www2.ib.unicamp.br/fnjv/collection.php?fnjv=50590
- FNJV 50591 - https://www2.ib.unicamp.br/fnjv/collection.php?fnjv=50591
- FNJV 50592 - https://www2.ib.unicamp.br/fnjv/collection.php?fnjv=50592
- FNJV 50593 - https://www2.ib.unicamp.br/fnjv/collection.php?fnjv=50593

### New Species Registration

The following information was supplied regarding the registration of a newly described species: Publication LSID: urn:lsid:zoobank.org:pub:E2CB2367-561F-461E-87A4-672AC6205875 *Allobates kamilae*, species LSID: urn:lsid:zoobank.org:act:350D7688-B957-41EF-AD81-82A417661ED9
