# Supplementary material for: A new nurse frog of the Allobates tapajos species complex (Anura: Aromobatidae) from the upper Madeira River, Brazilian Amazonia
Source: PeerJ. 2022 Aug 3;10:e13751. doi: 10.7717/peerj.13751 (PMC9356586; doi:10.7717/peerj.13751)
Supplement: Supplemental Information 1 [file peerj-10-13751-s001.docx]

A new nurse frog of the *Allobates tapajo*s species complex (Anura: Aromobatidae) from the upper Madeira River, Brazilian Amazonia

Miquéias Ferrão^1,2^, James Hanken^1^ and Albertina P. Lima^2^

^1^ Museum of Comparative Zoology, Harvard University, Cambridge, Massachusetts, USA. ^2^ Coordenação de Pesquisas em Biodiversidade, Instituto Nacional de Pesquisas da Amazônia, Manaus, Amazonas, Brazil.

**Supplementary files**

**Table S1.** Museum voucher and GenBank accession number of specimens used in phylogenetic analyses. Accession numbers in bold font denote sequences generated by this study.

| Species | Voucher | 12S | 16S | ND1 | COI | CYTB |
| --- | --- | --- | --- | --- | --- | --- |
| *Allobates* aff. *granti* | 125PG | JN690205 | JN690931 | – | – | – |
| *A.* aff. *magnussoni* | 977126 | MT627173 | MT627173 | MT627173 | MT627173 | MT627173 |
| *A.* aff. *melanolaemus* | MTR28013 | MT627203 | MT627203 | MT627203 | MT627203 | MT627203 |
| *A.* aff. *olfersioides 1* | MTR17821 | – | KDQF01003353 | – | – | – |
| *A.* aff. *olfersioides 2* | MTR16435 | MT627202 | MT627202 | MT627202 | MT627202 | MT627202 |
| *A.* aff. *olfersioides 3* | JFT959 | – | KDQF01002701 | – | – | – |
| *A.* aff. *trilineatus 1* | FGZC3247 | MT627185 | MT627185 | MT627185 | MT627185 | MT627185 |
| *A.* aff. *trilineatus 2* | JMP2313 | MT627195 | MT627195 | MT627195 | MT627195 | MT627195 |
| *A.* aff. *undulatus* | AMNHA159139 | DQ283044 | DQ283044 | – | DQ502756 | DQ502459 |
| *A. algorei* | TNHCFS5551 | HQ290950 | HQ290950 | HQ290950 | – | HQ290530 |
| *A. amissibilis* | PK3798 | MT627204 | MT627204 | MT627204 | MT627204 | MT627204 |
| *A. bacurau* | INPAH35401 | – | KU195701 | – | – | – |
| *A. caeruleodactylus* | MTR10227 | MT627199 | MT627199 | MT627199 | MT627199 | MT627199 |
| *A. carajas* | BM163 | MT627183 | MT627183 | MT627183 | MT627183 | MT627183 |
| *A. chalcopis* | Alca1 | MT627182 | MT627182 | MT627182 | MT627182 | – |
| *A. conspicuus/subfolionidificans* | FGZC3279 | MT627186 | MT627186 | MT627186 | MT627186 | MT627186 |
| *A. crombiei* | AF1097 | MT627174 | MT627174 | MT627174 | MT627174 | MT627174 |
| *A. femoralis* | AF3224 | MT627179 | MT627179 | MT627179 | MT627179 | MT627179 |
| *A. flaviventris* | HJ545 | MT627192 | MT627192 | MT627192 | MT627192 | MT627192 |
| *A. fratisenescus* | QCAZ54377 | – | MF624172 | – | – | MF614174 |
| *A. gasconi* SL1 | MNRJ 91682 | – | KY886573 | – | – | KY886614 |
| *A. gasconi* SL1 | OMNH36636 | DQ502209 | DQ502209 | – | DQ502898 | DQ502644 |
| *A. gasconi* SL1 | MPEG13003 | DQ502052 | DQ502052 | – | DQ502777 | DQ502483 |
| *A. gasconi* SL1 | MNRJ 90229 | – | KY886570 | – | – | KY886612 |
| *A. gasconi* SL1 | MNRJ 90230 | – | KY886571 | – | – | KY886613 |
| *A. gasconi* SL2 | MCP13630 | – | KY886577 | – | – | KY886618 |
| *A. gasconi* SL2 | MNRJ91665 | – | KY886576 | – | – | KY886617 |
| *A. gasconi* SL2 | MNRJ91679 | – | KY886578 | – | – | KY886619 |
| *A. gasconi* SL2 | MNRJ91683 | – | KY886574 | – | – | KY886615 |
| *A. gasconi* SL2 | MNRJ91684 | – | KY886575 | – | – | KY886616 |
| *A. gasconi* SL3 | APL14410 | – | KJ747333 | – | – | – |
| *A. gasconi* SL3 | APL14411 | – | KJ747334 | – | – | – |
| *A. gasconi* SL4 | HJ480 | MT627191 | MT627191 | MT627191 | MT627191 | MT627191 |
| *A. gasconi* SL4 | HJ299 | – | KDQF01002640 | – | – | – |
| *A. goianus* | SAMA8574 | MT627207 | MT627207 | MT627207 | MT627207 | MT627207 |
| *A. granti* | AF1998 | MT627176 | MT627176 | MT627176 | MT627176 | MT627176 |
| *A. grillicantus* | MPEG43046 | – | MW220039 | – | – | – |
| *A. grillisimilis* | MTR12749 | MT627200 | MT627200 | MT627200 | MT627200 | MT627200 |
| *A. hodli* | ABU2194 | – | KX044279 | – | – | – |
| *A. humilis/pittieri* | CVULA5690 | KJ940454 | KJ940454 | – | – | – |
| *A. insperatus/juami* | JMP1703 | MT627193 | MT627193 | MT627193 | MT627193 | MT627193 |
| *A. juanii/ranoides* | ARA2394 | DQ502271 | DQ502271 | – | DQ502933 | DQ502702 |
| *A. kamilae* sp. nov. clade E | HJ285 | MT627189 | MT627189 | MT627189 | MT627189 | MT627189 |
| *A. kamilae* sp. nov. clade E | INPAH42921 | – | **MZ667620** | – | – | – |
| *A. kamilae* sp. nov. clade E | INPAH42888 | – | **MZ667621** | – | – | – |
| *A. kamilae* sp. nov. clade E | INPAH42896 | – | **MZ667622** | – | – | – |
| *A. kamilae* sp. nov. clade E | INPAH42919 | – | **MZ667626** | – | – | – |
| *A. kamilae* sp. nov. clade E | INPAH42964 | – | **MZ667627** | – | – | – |
| *A. kamilae* sp. nov. clade E | INPAH42937 | – | **MZ667628** | – | – | – |
| *A. kamilae* sp. nov. clade E | INPAH42965 | – | **MZ667629** | – | – | – |
| *A. kamilae* sp. nov. clade E | INPAH42948 | – | **MZ667630** | – | – | – |
| *A. kamilae* sp. nov. clade E | INPAH42920 | – | **MZ667631** | – | – | – |
| *A. kamilae* sp. nov. clade E | INPAH42967 | – | **MZ667632** | – | – | – |
| *A. kamilae* sp. nov. clade E | INPAH42901 | – | **MZ667633** | – | – | – |
| *A. kingsburyi* | QCAZ16523 | AY364549 | HQ290963 | – | – | HQ290541 |
| *A. magnussoni* | BM168 | MT627184 | MT627184 | MT627184 | MT627184 | MT627184 |
| *A. marchesianus* | AJC2498 | MT627180 | MT627180 | MT627180 | MT627180 | MT627180 |
| *A. masniger* | MTR10155 | MT627198 | MT627198 | MT627198 | MT627198 | MT627198 |
| *A. melanolaemus* | NMP6V711404 | – | MT524148 | – | – | – |
| *A. nidicola* | MPEG13821 | DQ502101 | DQ502101 | – | – | DQ502533 |
| *A. niputidea* | MUJ3520 | DQ502272 | DQ502272 | – | DQ502934 | DQ502703 |
| *A. nunciatus* | MPEG36777 | MT627196 | MT627196 | MT627196 | MT627196 | MT627196 |
| *A. olfersioides* | MNRJ79897 | MF624178 | MF624178 | – | – | MF614175 |
| *A. ornatus* | MHNSM22863 | – | EU342550 | – | – | – |
| *A. paleci* | CHUFPB30253 | – | OK349681 | – | – | – |
| *A. paleovarzensis* | JMP2196 | MT627194 | MT627194 | MT627194 | MT627194 | – |
| *A.* sp. "Huanuco" | FGZC3348 | MT627187 | MT627187 | MT627187 | MT627187 | MT627187 |
| *A.* sp. "Neblina" | MTR15537 | MT627201 | MT627201 | MT627201 | MT627201 | MT627201 |
| *A.* sp. "Ucayali" | GGU684 | – | MT524137 | – | – | – |
| *A. sumtuosus* | AF2212 | MT627177 | MT627177 | MT627177 | MT627177 | MT627177 |
| *A. talamancae* | QCAZ35236 | MT627205 | MT627205 | MT627205 | MT627205 | MT627205 |
| *A. tapajos* clade A | MJH3973 | DQ502110 | DQ502110 | – | DQ502820 | DQ502542 |
| *A. tapajos* clade A | LSUMZ15176 | DQ502046 | DQ502046 | – | DQ502772 | DQ502477 |
| *A. tapajos* clade A | LSUMZ15296 | EU342545 | EU342545 | – | – | – |
| *A. tapajos* clade A | LSUMZ15291 | EU342546 | EU342546 | – | – | – |
| *A. tapajos* clade A | INPAH36568 | – | KX524489 | – | – | – |
| *A. tapajos* clade A | APL12965 | – | KR047027 | – | – | – |
| *A. tapajos* clade A | APL12968 | – | KR047028 | – | – | – |
| *A. tapajos* clade A | BM004 | – | KDQF01001798 | – | – | – |
| *A. tapajos* clade A | BM032 | – | KDQF01001805 | – | – | – |
| *A. tapajos* clade B | MTR10084 | MT627197 | MT627197 | MT627197 | MT627197 | MT627197 |
| *A. tapajos* clade C | MPEG13383 | DQ502241 | DQ502241 | – | DQ502912 | DQ502673 |
| *A. tapajos* clade C | MPEG13393 | DQ502218 | DQ502218 | – | – | DQ502652 |
| *A. tapajos* clade C | MPEG13387 | DQ502217 | DQ502217 | – | – | DQ502651 |
| *A. tapajos* clade C | MPEG13394 | DQ502204 | DQ502204 | – | – | DQ502639 |
| *A. tapajos* clade C | LSUMZ17554 | EU342547 | EU342547 | – | – | – |
| *A. tapajos* clade C | APL20042 | – | **MZ667619** | – | – | – |
| *A. tapajos* clade C | APL20015 | – | **MZ667618** | – | – | – |
| *A. tapajos* clade D | AF1906 | MT627175 | MT627175 | MT627175 | MT627175 | MT627175 |
| *A. tapajos* clade D | 100PG | – | EU201069 | – | – | – |
| *A. tapajos* clade D | AF1658 | – | KDQF01000622 | – | – | – |
| *A. tapajos* clade D | AF2072 | – | KDQF01000821 | – | – | – |
| *A. tapajos* clade F | AAGARDA12452 | – | OK349679 | – | – | – |
| *A. tapajos* clade F | CHUFPB30308 | – | OK349680 | – | – | – |
| *A. tinae* | HJ298 | MT627190 | MT627190 | MT627190 | MT627190 | MT627190 |
| *A. trilineatus* | AF4493 | – | MT524111 | – | – | – |
| *A. undulatus* | AJC3040 | MT627181 | MT627181 | MT627181 | MT627181 | MT627181 |
| *A. zaparo* | USNM546405 | DQ502026 | DQ502026 | – | DQ502752 | DQ502455 |
| *Ameerega hahneli* | AF2673 | MT627178 | MT627178 | MT627178 | MT627178 | MT627178 |
| *Anomaloglossus stepheni* | MJH3928 | DQ502107 | DQ502107 | – | DQ502818 | DQ502539 |
| *Aromobates saltuensis/nocturnus* | TNHCFS5541/AMNHA130042 | HQ290970 | HQ290970 | HQ290970 | DQ502860 | DQ502592 |
| *Leucostethus fugax* | QCAZ16513 | HQ290958 | HQ290958 | HQ290958 | – | HQ290538 |
| *Colostethus brachistriatus* | CZPDUV4603 | MF624204 | MF624204 | – | MF614304 | MF614198 |
| *Dendrobates auratus* | MVZHerp149723 | JX564862 | JX564862 | JX564862 | JX564862 | JX564862 |
| *Epipedobates boulengeri* | UMMZ227952/QCAZ16574 | HQ290997 | HQ290997 | HQ290997 | DQ502742 | DQ502447 |
| *Mannophryne collaris* | FS5523 | MT627188 | MT627188 | MT627188 | MT627188 | MT627188 |
| *Phyllobates terribilis* | TNHC64420/AMNHA118566 | HQ291006 | HQ291006 | HQ291006 | DQ502861 | DQ502593 |
| *Rheobates palmatus* | RHEOPALM | MT627206 | MT627206 | MT627206 | MT627206 | MT627206 |
| *Silverstoneia nubicola/erasmios* | TNHCFS4942/MAR336 | HQ290966 | HQ290966 | HQ290966 | MF614333 | MF614237 |

**Table S2.** Morphometric measurements (in mm) of adults of the type series of *Allobates kamilae* sp. nov. (clade E) and *Allobates tapajos* (clade A). Measurement acronyms are defined in the text. Abbreviations: INPAH, museum voucher catalog numbers; FN, field numbers; SP, species; AK, *A. kamilae* sp. nov.; AT, *A. tapajos*; M, male; F, female.

| INPAH | FN | SP | Sex | SVL | HL | HW | SL | EN | IN | EL | IO | TYM | FAL | UAL | HANDI | HANDII | HANDIII | HANDIV | WFD | TL | FL | THL | DPT | WTT | WTD | WPF |
| --- | --- | --- | --- | --- | --- | --- | --- | --- | --- | --- | --- | --- | --- | --- | --- | --- | --- | --- | --- | --- | --- | --- | --- | --- | --- | --- |
| 42878 | APL16619 | AK | M | 14.53 | 5.1 | 5.3 | 2.3 | 1.65 | 2 | 2.15 | 4.1 | 1.1 | 3.3 | 3.3 | 2.6 | 2.8 | 3.75 | 2.3 | 0.5 | 7.2 | 6.7 | 7.5 | 0.5 | 0.15 | 0.6 | 0.4 |
| 42879 | APL16963 | AK | M | 16.68 | 5.5 | 5.8 | 2.5 | 1.8 | 2.4 | 2.2 | 4.6 | 1.25 | 3.3 | 3.5 | 2.75 | 2.6 | 3.8 | 2.55 | 0.55 | 7.8 | 6.8 | 7.8 | 0.5 | 0.25 | 0.6 | 0.4 |
| 42880 | APL16421 | AK | M | 15.64 | 5.1 | 5.7 | 1.95 | 1.5 | 2.1 | 2.05 | 4.6 | 0.9 | 3.6 | 3.8 | 2.75 | 2.85 | 3.85 | 2.5 | 0.55 | 7.8 | 7.2 | 7.9 | 0.55 | 0.25 | 0.75 | 0.35 |
| 42881 | APL2236 | AK | M | 16.39 | 5.6 | 5.3 | 2.1 | 1.55 | 1.9 | 2.35 | 4.8 | 0.8 | 3.1 | 3 | 2.8 | 2.7 | 3.75 | 2.15 | 0.6 | 7.6 | 6.4 | 7.9 | 0.5 | 0.15 | 0.7 | 0.4 |
| 42882 | APL2233 | AK | M | 16.89 | 5.3 | 5.7 | 2.5 | 1.75 | 2.2 | 2.25 | 4.9 | 0.8 | 3 | 3.5 | 2.95 | 2.8 | 3.85 | 2.6 | 0.5 | 7.9 | 7.3 | 8.1 | 0.45 | 0.3 | 0.65 | 0.4 |
| 42884 | APL16432 | AK | M | 15.81 | 5.5 | 5.2 | 2.3 | 2.25 | 2.1 | 1.75 | 4.3 | 0.85 | 3.2 | 3.3 | 2.55 | 2.55 | 3.7 | 2.7 | 0.55 | 7.6 | 6.4 | 7.7 | 0.5 | 0.2 | 0.65 | 0.35 |
| 42886 | APL16854 | AK | M | 15.89 | 5.4 | 5.4 | 2.15 | 1.55 | 2.3 | 2.25 | 4.5 | 1.4 | 3.4 | 3.4 | 2.75 | 2.7 | 3.9 | 2.5 | 0.55 | 7.8 | 6.2 | 7.6 | 0.55 | 0.2 | 0.65 | 0.35 |
| 42887 | RM81 | AK | M | 15.26 | 5.5 | 5.7 | 2.7 | 1.75 | 2.1 | 2.2 | 4.7 | 0.75 | 3.1 | 3.6 | 2.6 | 3.4 | 3.8 | 2.65 | 0.6 | 7.9 | 7 | 8 | 0.5 | 0.3 | 0.75 | 0.35 |
| 42890 | APL15887 | AK | M | 16.77 | 6.9 | 6.4 | 2.35 | 1.7 | 2.4 | 1.95 | 4.5 | 1.1 | 3.3 | 3.3 | 3.05 | 2.85 | 4.15 | 2.8 | 0.65 | 8.2 | 6.8 | 7.6 | 0.55 | 0.3 | 0.75 | 0.4 |
| 42892 | APL16611 | AK | M | 15.57 | 4.8 | 4.4 | 2.3 | 1.55 | 2.1 | 2.35 | 4.1 | 0.8 | 3.5 | 3.4 | 2.95 | 2.9 | 4.05 | 2.55 | 0.65 | 7.5 | 7.9 | 7.9 | 0.7 | 0.2 | 0.65 | 0.35 |
| 42893 | APL15848 | AK | M | 16.66 | 5.1 | 5.5 | 2.2 | 1.65 | 2.2 | 2.25 | 4.7 | 0.9 | 3.4 | 3.7 | 2.35 | 2.85 | 4.1 | 2.75 | 0.55 | 7.8 | 6.9 | 7.9 | 0.6 | 0.3 | 0.65 | 0.35 |
| 42897 | APL16615 | AK | M | 15.98 | 5.9 | 5.5 | 2.5 | 1.65 | 2.2 | 2.35 | 4.3 | 1.1 | 3.5 | 3.4 | 2.95 | 2.95 | 4.1 | 2.75 | 0.65 | 7.8 | 7.1 | 7.7 | 0.65 | 0.2 | 0.75 | 0.4 |
| 42899 | APL14630 | AK | M | 15.71 | 5.3 | 5.2 | 2 | 1.5 | 2.3 | 2.3 | 4.3 | 0.85 | 3.5 | 3.8 | 2.6 | 2.55 | 3.25 | 2.35 | 0.5 | 8.2 | 7 | 8 | 0.5 | 0.25 | 0.65 | 0.3 |
| 42900 | APL2242 | AK | M | 14.97 | 5.5 | 5.6 | 2.05 | 1.5 | 1.9 | 2.3 | 4.6 | 0.8 | 3.1 | 2.8 | 2.55 | 2.45 | 3.25 | 2.2 | 0.55 | 6.8 | 6.1 | 6.8 | 0.45 | 0.2 | 0.6 | 0.35 |
| 42902 | APL14837 | AK | M | 15.34 | 5.2 | 5.3 | 2.45 | 1.6 | 2.1 | 2.25 | 4.4 | 0.75 | 3.1 | 3.3 | 2.65 | 2.7 | 3.75 | 2.6 | 0.55 | 7.4 | 6.6 | 7.5 | 0.45 | 0.15 | 0.7 | 0.4 |
| 42903 | APL16464 | AK | M | 15.88 | 6.7 | 6.3 | 2.05 | 1.55 | 2.2 | 2.35 | 4.7 | 0.65 | 3.4 | 3 | 2.5 | 2.45 | 3.6 | 2.4 | 0.6 | 6.8 | 6.7 | 6.4 | 0.55 | 0.25 | 0.7 | 0.35 |
| 42904 | APL16616 | AK | M | 15.32 | 4.8 | 5.5 | 2.75 | 1.8 | 2.2 | 2.35 | 4.4 | 0.9 | 3.2 | 3.1 | 2.65 | 2.4 | 3.85 | 2.5 | 0.6 | 7.5 | 6.3 | 7.4 | 0.55 | 0.3 | 0.75 | 0.4 |
| 42905 | APL16620 | AK | M | 15.28 | 4.8 | 5.3 | 2.2 | 1.6 | 2.1 | 2.3 | 4.3 | 0.9 | 3.2 | 3.4 | 2.5 | 2.7 | 3.75 | 2.65 | 0.6 | 7.5 | 6.7 | 7.6 | 0.5 | 0.3 | 0.75 | 0.4 |
| 42909 | APL15878 | AK | M | 15.64 | 4.5 | 5.5 | 2.05 | 1.6 | 2.3 | 2.1 | 4.4 | 1.1 | 3.2 | 3.1 | 2.25 | 2.65 | 4.05 | 2.85 | 0.7 | 7.6 | 6.4 | 7.4 | 0.65 | 0.15 | 0.85 | 0.4 |
| 42911 | APL2241 | AK | M | 15.31 | 5.3 | 5.3 | 2.3 | 1.65 | 2.1 | 2.1 | 4.6 | 0.9 | 3.3 | 3.2 | 2.85 | 2.95 | 3.9 | 2.5 | 0.6 | 7.3 | 6.7 | 7.4 | 0.5 | 0.2 | 0.75 | 0.4 |
| 42913 | APL14593 | AK | M | 15.2 | 5 | 5.3 | 2.25 | 1.75 | 2.2 | 2.5 | 4.8 | 1.0 | 3.1 | 4 | 3.25 | 3.1 | 3.55 | 3.35 | 0.8 | 7.7 | 7 | 7.5 | 0.6 | 0.3 | 0.85 | 0.275 |
| 42917 | APL14608 | AK | M | 15.79 | 5.5 | 5.9 | 1.95 | 1.45 | 2.2 | 2.05 | 4.4 | 1 | 3.3 | 3.6 | 2.6 | 2.7 | 3.85 | 2.45 | 0.65 | 7.7 | 6.6 | 7.7 | 0.5 | 0.25 | 0.7 | 0.4 |
| 42919 | APL14791 | AK | M | 16.6 | 5.3 | 5.8 | 2.6 | 1.75 | 2.2 | 2.65 | 4.7 | 1.0 | 3.5 | 4.2 | 3.25 | 3.1 | 4.3 | 2.75 | 0.6 | 7.6 | 7.3 | 7.3 | 0.5 | 0.35 | 0.6 | 0.2 |
| 42920 | APL14970 | AK | M | 15.4 | 5.2 | 5.5 | 2.7 | 1.8 | 2.1 | 2.85 | 4.4 | 1.0 | 3.4 | 4.1 | 3.25 | 3 | 4.35 | 2.95 | 0.7 | 7.7 | 6.9 | 7.7 | 0.65 | 0.3 | 0.85 | 0.2 |
| 42923 | APL14840 | AK | M | 16.06 | 5.5 | 5.4 | 2.3 | 1.55 | 2.1 | 2.25 | 4.6 | 1 | 3.2 | 3.5 | 2.5 | 2.75 | 3.95 | 2.55 | 0.6 | 6.8 | 6.8 | 6.9 | 0.5 | 0.3 | 0.65 | 0.3 |
| 42924 | APL2040 | AK | M | 14.76 | 5.4 | 5.3 | 1.75 | 1.35 | 2 | 2.05 | 4.7 | 1 | 3.4 | 3.4 | 2.7 | 2.75 | 3.85 | 2.4 | 0.5 | 7.6 | 6.5 | 7.8 | 0.45 | 0.2 | 0.65 | 0.35 |
| 42926 | RM83 | AK | M | 16.34 | 6 | 6 | 2.25 | 1.75 | 2.2 | 2.2 | 4.6 | 1.75 | 3.4 | 3.3 | 2.95 | 3.1 | 4.35 | 2.85 | 0.65 | 8.19 | 6.7 | 7.6 | 0.7 | 0.35 | 0.85 | 0.4 |
| 42928 | APL2240 | AK | M | 14.96 | 5.1 | 5 | 2.2 | 1.45 | 2.1 | 2.4 | 4.8 | 0.8 | 2.3 | 3.2 | 2.4 | 2.4 | 3.45 | 2.05 | 0.5 | 6.5 | 6.4 | 7.4 | 0.45 | 0.25 | 0.65 | 0.35 |
| 42929 | APL14975 | AK | M | 15.04 | 5 | 5.1 | 1.9 | 1.55 | 2 | 1.75 | 4.5 | 1.15 | 3.3 | 3.2 | 2.65 | 2.6 | 3.65 | 2.3 | 0.5 | 7 | 6.5 | 7.4 | 0.35 | 0.15 | 0.6 | 0.35 |
| 42930 | APL14592 | AK | M | 15.89 | 5.4 | 5.5 | 2.05 | 1.55 | 2.1 | 2.15 | 4.6 | 1.15 | 3.5 | 2.9 | 2.7 | 2.85 | 4.15 | 2.75 | 0.6 | 7.4 | 6.6 | 7.7 | 0.65 | 0.25 | 0.65 | 0.4 |
| 42932 | APL2243 | AK | M | 15.85 | 5 | 5.4 | 2.25 | 1.6 | 2.1 | 2.15 | 4.8 | 1 | 3.3 | 3.5 | 2.65 | 2.8 | 3.8 | 2.55 | 0.55 | 7.2 | 6.8 | 8 | 0.55 | 0.25 | 0.75 | 0.35 |
| 42935 | APL14751 | AK | M | 15.77 | 5.4 | 5.3 | 2.15 | 1.45 | 2.1 | 2.2 | 4.5 | 0.8 | 3 | 3.2 | 2.9 | 2.95 | 3.95 | 2.55 | 0.6 | 7.3 | 6.6 | 7.5 | 0.5 | 0.2 | 0.75 | 0.4 |
| 42936 | RM643 | AK | M | 17.01 | 6 | 5.8 | 2.3 | 1.65 | 2.2 | 2.55 | 4.6 | 1 | 3.4 | 3.5 | 2.9 | 2.85 | 3.8 | 2.4 | 0.45 | 7.8 | 6.8 | 7.7 | 0.5 | 0.3 | 0.65 | 0.3 |
| 42937 | APL14838 | AK | M | 16.39 | 5.3 | 5.4 | 2.15 | 1.55 | 2.2 | 2.45 | 4.6 | 0.95 | 3.1 | 3.5 | 2.1 | 2.65 | 4.05 | 2.8 | 0.65 | 7.3 | 6.9 | 7.4 | 0.5 | 0.2 | 0.85 | 0.35 |
| 42943 | APL14841 | AK | M | 15.4 | 5.2 | 5.3 | 2.1 | 1.6 | 2.1 | 2.05 | 4.6 | 0.85 | 3.3 | 3.6 | 2.75 | 2.65 | 3.9 | 2.55 | 0.55 | 6.9 | 7 | 7.1 | 0.5 | 0.2 | 0.65 | 0.35 |
| 42944 | APL2237 | AK | M | 16.22 | 5.9 | 6 | 2.2 | 1.6 | 2.1 | 2.2 | 5 | 0.9 | 3.1 | 3.2 | 2.85 | 2.8 | 3.85 | 2.5 | 0.6 | 7.4 | 6.8 | 7.5 | 0.5 | 0.25 | 0.9 | 0.4 |
| 42945 | APL14702 | AK | M | 14.75 | 5.1 | 4.5 | 1.8 | 1.4 | 2.2 | 2.15 | 4.3 | 0.8 | 3.4 | 3.8 | 2.6 | 2.65 | 3.4 | 2.4 | 0.45 | 6.9 | 6.9 | 7 | 0.55 | 0.25 | 0.65 | 0.35 |
| 42947 | APL14974 | AK | M | 14.99 | 5.4 | 5 | 1.95 | 1.45 | 2 | 2.25 | 4.1 | 0.8 | 3 | 3.2 | 2.55 | 2.5 | 3.6 | 2.4 | 0.5 | 7.1 | 5.8 | 7.2 | 0.35 | 0.15 | 0.6 | 0.35 |
| 42948 | APL14972 | AK | M | 14.9 | 5 | 5.4 | 2.6 | 1.75 | 2.3 | 2.5 | 4.5 | 0.85 | 3.1 | 4 | 3.1 | 3.1 | 4.3 | 2.75 | 0.7 | 7.7 | 7 | 7.7 | 0.5 | 0.3 | 0.9 | 0.2 |
| 42952 | APL14839 | AK | M | 15.96 | 5.1 | 5.4 | 2.15 | 1.6 | 2.2 | 2.2 | 4.3 | 1.05 | 3.3 | 3.6 | 2.65 | 2.35 | 3.85 | 2.55 | 0.55 | 7.7 | 6.5 | 7.8 | 0.5 | 0.2 | 0.55 | 0.35 |
| 42955 | RM781 | AK | M | 16.13 | 5.4 | 5.4 | 1.9 | 1.2 | 2.1 | 2.25 | 4.8 | 1 | 3.3 | 3.5 | 2.8 | 2.85 | 3.9 | 2.5 | 0.6 | 6.7 | 7.1 | 7.4 | 0.45 | 0.25 | 0.7 | 0.4 |
| 42956 | APL2234 | AK | M | 16.85 | 5.5 | 5.8 | 2.05 | 1.5 | 2.1 | 2.25 | 4.8 | 0.8 | 3.2 | 3.2 | 2.7 | 2.7 | 3.9 | 2.4 | 0.55 | 7.8 | 7.2 | 8.1 | 0.55 | 0.25 | 0.65 | 0.35 |
| 42957 | APL15917 | AK | M | 15.25 | 4.9 | 5.5 | 2.1 | 1.6 | 2 | 1.95 | 4.4 | 0.7 | 3.3 | 3.3 | 2.75 | 2.7 | 3.75 | 2.35 | 0.5 | 7.1 | 6.8 | 7.5 | 0.5 | 0.25 | 0.65 | 0.35 |
| 42959 | APL16413 | AK | M | 16.09 | 5 | 5.6 | 2.15 | 1.65 | 2.1 | 2.05 | 4.5 | 1.05 | 3.2 | 3.5 | 2.6 | 2.95 | 4.05 | 2.75 | 0.6 | 7.8 | 6.6 | 7.6 | 0.65 | 0.3 | 0.7 | 0.35 |
| 42961 | RM476 | AK | M | 14.94 | 5.5 | 5.8 | 2.05 | 1.6 | 2.1 | 2.05 | 4.7 | 0.7 | 3 | 3.5 | 2.65 | 2.8 | 3.85 | 2.5 | 0.55 | 7.1 | 6.6 | 8 | 0.55 | 0.25 | 0.6 | 0.35 |
| 42963 | APL14789 | AK | M | 16.61 | 5.7 | 6 | 2.1 | 1.65 | 2.4 | 2.55 | 5 | 0.75 | 3.5 | 3.9 | 2.9 | 3 | 4 | 2.8 | 0.5 | 8.19 | 7.6 | 8.4 | 0.65 | 0.3 | 0.7 | 0.35 |
| 42965 | APL14700 | AK | M | 16.9 | 5.2 | 5.9 | 2.85 | 1.85 | 2.3 | 2.65 | 4.9 | 0.9 | 3.3 | 4.5 | 3.4 | 3.25 | 4.35 | 3 | 0.65 | 8.2 | 7.8 | 7.7 | 0.65 | 0.25 | 0.9 | 0.225 |
| 42966 | APL14973 | AK | M | 15.19 | 5.1 | 5.3 | 2.6 | 1.75 | 2.1 | 2.25 | 4.3 | 1 | 3.3 | 3 | 2.55 | 2.6 | 3.8 | 2.55 | 0.55 | 7.4 | 6.6 | 7.5 | 0.5 | 0.2 | 0.6 | 0.35 |
| 42967 | APL14609 | AK | M | 17.4 | 5.7 | 5.9 | 2.35 | 1.45 | 2.3 | 2.6 | 5.1 | 1.00 | 3.8 | 4.6 | 3.65 | 3.5 | 4.75 | 3.25 | 0.7 | 8.3 | 7.7 | 7.7 | 0.5 | 0.3 | 0.8 | 0.225 |
| 42877 | APL16617 | AK | F | 17.71 | 5.9 | 5.6 | 2.6 | 1.95 | 2.5 | 2.45 | 4.7 | 1.25 | 3.1 | 3.3 | 2.8 | 2.5 | 4 | 2.8 | 0.7 | 7.5 | 7.3 | 7.7 | 0.55 | 0.3 | 0.75 | 0.35 |
| 42885 | APL2247 | AK | F | 17.65 | 5.7 | 5.9 | 2 | 1.6 | 2.4 | 2.05 | 4.9 | 0.95 | 3.6 | 3.8 | 3.45 | 3.3 | 4.2 | 2.85 | 0.6 | 8 | 8 | 8.5 | 0.5 | 0.2 | 0.75 | 0.35 |
| 42889 | APL2235 | AK | F | 17.48 | 5.5 | 5.9 | 2.55 | 1.75 | 2.3 | 2.25 | 4.8 | 1 | 3.6 | 3.3 | 2.8 | 2.4 | 3.7 | 2.6 | 0.55 | 7.7 | 7.3 | 8.2 | 0.5 | 0.25 | 0.7 | 0.3 |
| 42894 | APL15832 | AK | F | 16.53 | 5.1 | 5.8 | 2.5 | 1.75 | 2.3 | 2.1 | 4.7 | 0.95 | 3.6 | 3.7 | 2.7 | 2.9 | 4 | 2.9 | 0.55 | 7.8 | 5 | 7.6 | 0.55 | 0.25 | 0.55 | 0.35 |
| 42906 | APL2198 | AK | F | 16.12 | 5.3 | 5.6 | 2.4 | 1.7 | 2.2 | 2.3 | 4.9 | 0.75 | 3 | 3.6 | 2.75 | 2.75 | 3.95 | 2.65 | 0.6 | 8 | 6.8 | 7.8 | 0.6 | 0.2 | 0.65 | 0.35 |
| 42908 | APL16612 | AK | F | 17.02 | 6.2 | 5.7 | 2.45 | 1.7 | 2.4 | 2.3 | 4.7 | 1.15 | 3.4 | 3.6 | 2.5 | 2.65 | 3.8 | 2.55 | 0.5 | 8 | 6.6 | 8.2 | 0.55 | 0.25 | 0.65 | 0.3 |
| 42912 | APL16349 | AK | F | 15.48 | 5.1 | 5.4 | 2.1 | 1.65 | 2 | 2.2 | 3.9 | 0.95 | 3.3 | 3.8 | 2.5 | 2.55 | 3.75 | 2.25 | 0.45 | 7.7 | 6.6 | 7.8 | 0.7 | 0.2 | 0.5 | 0.3 |
| 42914 | RM281 | AK | F | 17.24 | 5.5 | 5.5 | 2.15 | 1.7 | 2.3 | 2.3 | 4.7 | 0.9 | 3.6 | 3.6 | 2.7 | 2.65 | 3.95 | 2.55 | 0.55 | 7.8 | 7.2 | 7.9 | 0.5 | 0.25 | 0.6 | 0.3 |
| 42915 | APL2043 | AK | F | 15.17 | 5.5 | 5.3 | 2.25 | 1.65 | 2.2 | 2.2 | 4.4 | 0.6 | 3.1 | 3.4 | 2.85 | 2.8 | 3.7 | 2.45 | 0.55 | 7.5 | 6.5 | 7.1 | 0.55 | 0.2 | 0.65 | 0.3 |
| 42916 | RM280 | AK | F | 16.02 | 5.5 | 6 | 2.05 | 1.55 | 2.5 | 2.25 | 5 | 1.4 | 3.3 | 3.8 | 2.6 | 2.55 | 3.75 | 2.35 | 0.5 | 8.30 | 7.1 | 8 | 0.5 | 0.3 | 0.6 | 0.3 |
| 42925 | APL14959 | AK | F | 17 | 6 | 6.6 | 2.3 | 1.65 | 2.4 | 2.25 | 4.7 | 0.75 | 3.5 | 3.9 | 2.5 | 2.55 | 3.65 | 2.25 | 0.5 | 8.2 | 7 | 8.1 | 0.6 | 0.25 | 0.65 | 0.3 |
| 42927 | APL16462 | AK | F | 16.89 | 6.7 | 6.5 | 2.15 | 1.55 | 2.5 | 2.15 | 5 | 0.65 | 3.7 | 3.4 | 2.8 | 2.9 | 4.1 | 2.85 | 0.5 | 8.1 | 7.4 | 8.7 | 0.55 | 0.15 | 0.7 | 0.3 |
| 42931 | APL2197 | AK | F | 17.05 | 5.7 | 5.9 | 2.3 | 1.6 | 2.3 | 2.2 | 4.9 | 0.9 | 3.2 | 3.6 | 2.75 | 2.8 | 3.9 | 2.45 | 0.55 | 7.6 | 7.3 | 8 | 0.6 | 0.3 | 0.8 | 0.3 |
| 42933 | APL2245 | AK | F | 16.42 | 5.4 | 5.3 | 2.3 | 1.6 | 2 | 2.4 | 4.7 | 1 | 2.8 | 3.5 | 2.8 | 2.8 | 3.85 | 2.35 | 0.55 | 7.4 | 6.4 | 7.7 | 0.5 | 0.2 | 0.65 | 0.35 |
| 42934 | APL2042 | AK | F | 15.98 | 5.7 | 6 | 2.25 | 1.7 | 2.2 | 2.25 | 4.7 | 0.9 | 3.1 | 3.6 | 2.7 | 2.8 | 3.8 | 2.5 | 0.6 | 7.6 | 7 | 7.9 | 0.45 | 0.3 | 0.65 | 0.3 |
| 42938 | APL2222 | AK | F | 16.83 | 5.6 | 6 | 2.25 | 1.65 | 2.2 | 2.1 | 4.9 | 1.3 | 2.9 | 3 | 2.5 | 2.55 | 3.4 | 2.05 | 0.55 | 7.8 | 6.9 | 7.8 | 0.6 | 0.25 | 0.65 | 0.35 |
| 42939 | APL16962 | AK | F | 16.38 | 5.5 | 5.6 | 2.2 | 1.7 | 2.3 | 2.25 | 4.4 | 0.85 | 3.4 | 3.2 | 2.65 | 2.5 | 3.7 | 2.55 | 0.5 | 7.3 | 6.5 | 7.5 | 0.5 | 0.2 | 0.6 | 0.3 |
| 42940 | APL16461 | AK | F | 17.64 | 5.5 | 5.5 | 2.25 | 1.55 | 2.3 | 2.25 | 4.5 | 0.9 | 3.2 | 3.9 | 2.6 | 2.65 | 3.95 | 2.75 | 0.45 | 7.5 | 7 | 7 | 0.55 | 0.25 | 0.7 | 0.3 |
| 42941 | APL16434 | AK | F | 16.69 | 6.5 | 6.7 | 2.7 | 2 | 2.4 | 2.2 | 5 | 1.15 | 3.6 | 3.9 | 2.9 | 2.95 | 4.1 | 2.65 | 0.6 | 8 | 7.6 | 7.8 | 0.5 | 0.2 | 0.7 | 0.35 |
| 42942 | APL16433 | AK | F | 17.51 | 6.2 | 6.2 | 2.55 | 1.85 | 2.5 | 2.3 | 4.6 | 1.2 | 3.5 | 3.4 | 2.95 | 2.85 | 4.25 | 2.9 | 0.65 | 8.3 | 7.5 | 8.2 | 0.5 | 0.25 | 0.65 | 0.35 |
| 42950 | RM57 | AK | F | 16.6 | 5.5 | 5.9 | 2.3 | 1.6 | 2.2 | 2.55 | 4.9 | 0.7 | 2.9 | 3.3 | 2.6 | 2.95 | 4.1 | 2.85 | 0.55 | 7.7 | 6.9 | 8 | 0.55 | 0.25 | 0.7 | 0.35 |
| 42953 | RM309 | AK | F | 17.68 | 6.1 | 6 | 2.3 | 1.6 | 2.5 | 2.4 | 4.9 | 0.85 | 3.4 | 3.5 | 2.9 | 2.85 | 4.05 | 2.75 | 0.45 | 7.6 | 7 | 7.7 | 0.7 | 0.25 | 0.55 | 0.35 |
| 42958 | APL2244 | AK | F | 16.07 | 5.4 | 5.8 | 2.6 | 1.75 | 2.2 | 2 | 4.5 | 0.85 | 2.8 | 3.3 | 2.6 | 2.65 | 3.75 | 2.45 | 0.55 | 6.4 | 6.4 | 7.5 | 0.5 | 0.2 | 0.75 | 0.35 |
| 42962 | APL2041 | AK | F | 16.53 | 5.4 | 5.7 | 2.5 | 1.75 | 2.3 | 2.5 | 5 | 1.05 | 3.3 | 3.4 | 3.1 | 2.95 | 4.15 | 2.6 | 0.5 | 7.8 | 6.9 | 7.9 | 0.55 | 0.25 | 0.6 | 0.35 |
| 34402 | APL12980 | AT | M | 15.02 | 4.6 | 5.1 | 2 | 1.35 | 2.25 | 2.45 | 4.6 | 1.05 | 3.5 | 4.2 | 3 | 2.8 | 3.6 | 2.5 | 1.3 | 7.6 | 7 | 7.4 | 0.5 | 0.35 | 0.65 | 0.4 |
| 34403 | APL12100 | AT | M | 13.97 | 4.4 | 5.1 | 1.9 | 1.25 | 2.05 | 2.45 | 4.5 | 1 | 3.5 | 4 | 3 | 2.7 | 3.5 | 2.7 | 1.2 | 7.6 | 7 | 7.2 | 0.5 | 0.45 | 0.65 | 0.4 |
| 34404 | APL12965 | AT | M | 14.75 | 4.6 | 5 | 2 | 1.4 | 2.2 | 2.4 | 4.7 | 1.15 | 3.4 | 4 | 3 | 2.7 | 3.7 | 2.6 | 1.1 | 7.5 | 7.1 | 7.7 | 0.5 | 0.35 | 0.65 | 0.4 |
| 34405 | APL12976 | AT | M | 14.9 | 4.4 | 5.1 | 2.25 | 1.5 | 2.2 | 2.55 | 4.5 | 1 | 3.3 | 3.9 | 2.9 | 2.7 | 3.8 | 2.7 | 1.1 | 7.3 | 6.9 | 6.6 | 0.6 | 0.3 | 0.65 | 0.35 |
| 34406 | APL12101 | AT | M | 14.24 | 4.2 | 5.1 | 1.75 | 1.3 | 2.25 | 2.2 | 4.3 | 1 | 3.4 | 4 | 3.1 | 2.7 | 3.7 | 2.7 | 1.2 | 7.5 | 7 | 7 | 0.65 | 0.4 | 0.65 | 0.35 |
| 34410 | APL12975 | AT | M | 14.87 | 4.6 | 5.6 | 1.8 | 1.5 | 2.15 | 2.35 | 4.4 | 1 | 3.5 | 4 | 3.2 | 2.8 | 4 | 2.5 | 1.1 | 7.5 | 7.1 | 7.1 | 0.7 | 0.5 | 0.6 | 0.3 |
| 34411 | APL12982 | AT | M | 16.08 | 4.8 | 5.3 | 2.5 | 1.5 | 2.2 | 2.65 | 4.6 | 1.15 | 3.5 | 4 | 3.2 | 2.9 | 4 | 2.7 | 1 | 7.4 | 7 | 7.5 | 0.6 | 0.45 | 0.65 | 0.4 |
| 34412 | APL12102 | AT | M | 15.48 | 4.5 | 5.3 | 2 | 1.35 | 2.2 | 2.5 | 4.7 | 1 | 3.4 | 4 | 3 | 2.7 | 4 | 2.7 | 1.2 | 7.9 | 7.5 | 7.8 | 0.45 | 0.25 | 0.8 | 0.45 |
| 34414 | APL12977 | AT | M | 15.15 | 4.7 | 5.2 | 2.25 | 1.5 | 2.15 | 2.5 | 4.4 | 0.95 | 3.1 | 4.2 | 3.2 | 2.9 | 4.1 | 2.8 | 1.1 | 7.6 | 7.2 | 7.5 | 0.5 | 0.3 | 0.65 | 0.35 |
| 34415 | APL12974 | AT | M | 14.94 | 4.5 | 5.1 | 1.9 | 1.4 | 2.25 | 2.5 | 4.4 | 1 | 3 | 3.5 | 3 | 2.6 | 3.8 | 2.6 | 1 | 6.6 | 7 | 7.3 | 0.65 | 0.45 | 0.55 | 0.3 |
| 34416 | APL12979 | AT | M | 15.01 | 4.5 | 5 | 2 | 1.5 | 2.05 | 2.45 | 4.4 | 1 | 3.6 | 4.5 | 3 | 2.9 | 4 | 2.9 | 1.1 | 7.2 | 7 | 7.6 | 0.65 | 0.3 | 0.75 | 0.35 |
| 34418 | APL12983 | AT | M | 15.09 | 4.7 | 5.6 | 2.15 | 1.55 | 2.2 | 2.25 | 4.8 | 1.15 | 3.4 | 4 | 2.7 | 2.5 | 3.6 | 2.5 | 1.2 | 7.5 | 6.6 | 7.2 | 0.7 | 0.3 | 0.65 | 0.4 |
| 34423 | APL12981 | AT | M | 15.32 | 4.5 | 5.2 | 2.25 | 1.5 | 2.1 | 2.5 | 4.5 | 1.2 | 3.5 | 4.58 | 3.1 | 2.8 | 3.5 | 2.5 | 1 | 8 | 7.5 | 7.5 | 0.65 | 0.45 | 0.7 | 0.5 |
| 34425 | APL12978 | AT | M | 15.87 | 4.8 | 5.2 | 2.3 | 1.6 | 2.25 | 2.45 | 4.6 | 1.05 | 3.6 | 4 | 3 | 2.9 | 4 | 2.7 | 1.2 | 7.6 | 7.6 | 7.6 | 0.5 | 0.4 | 0.7 | 0.35 |
| 34407 | APL12972 | AT | F | 16.57 | 4.9 | 5.3 | 2.5 | 1.55 | 2.35 | 2.55 | 4.9 | 1.2 | 3.9 | 4 | 3.2 | 2.7 | 3.6 | 2.7 | 1.1 | 8.1 | 6.8 | 7.9 | 0.6 | 0.45 | 0.65 | 0.35 |
| 34408 | APL12969 | AT | F | 15.46 | 4.5 | 5.2 | 2.25 | 1.55 | 2.25 | 2.7 | 4.8 | 1.1 | 3.5 | 4.2 | 3 | 2.7 | 3.8 | 2.5 | 1.1 | 7.8 | 6.5 | 7.7 | 0.55 | 0.25 | 0.65 | 0.35 |
| 34409 | APL12968 | AT | F | 16.2 | 5 | 5.2 | 2.65 | 1.7 | 2.45 | 2.7 | 4.8 | 1 | 3.9 | 4 | 3.2 | 3 | 4 | 2.9 | 1.2 | 7.9 | 7.6 | 8 | 0.6 | 0.35 | 0.7 | 0.35 |
| 34413 | APL12973 | AT | F | 16.58 | 4.9 | 5.4 | 2.55 | 1.6 | 2.25 | 2.5 | 4.7 | 1 | 3.7 | 4.5 | 3.2 | 2.8 | 3.9 | 2.8 | 1.1 | 8 | 6.5 | 7.8 | 0.65 | 0.35 | 0.7 | 0.25 |
| 34417 | APL12984 | AT | F | 15.3 | 4.8 | 5.5 | 2.25 | 1.5 | 2.35 | 2.7 | 4.8 | 1.15 | 3.1 | 4 | 3.2 | 3 | 4 | 2.7 | 1.3 | 7.3 | 7 | 7.7 | 0.65 | 0.3 | 0.7 | 0.35 |
| 34419 | APL12970 | AT | F | 16.48 | 5 | 5.4 | 2.6 | 1.65 | 2.4 | 2.3 | 4.9 | 1.15 | 3.7 | 4 | 3.1 | 2.8 | 4.1 | 2.6 | 1.1 | 8 | 7.5 | 7.9 | 0.45 | 0.3 | 0.6 | 0.3 |
| 34420 | APL12967 | AT | F | 15.36 | 4.9 | 5.4 | 2.5 | 1.6 | 2.5 | 2.45 | 4.7 | 1.1 | 3.5 | 4.0 | 3 | 2.9 | 4 | 2.7 | 1.3 | 7.9 | 7.3 | 7.5 | 0.5 | 0.3 | 0.7 | 0.35 |
| 34421 | APL12971 | AT | F | 15.85 | 5.1 | 5.4 | 2.4 | 1.5 | 2.45 | 2.5 | 4.9 | 1 | 3.2 | 4.2 | 3.1 | 2.7 | 3.9 | 2.7 | 1.3 | 7.6 | 7.1 | 7.5 | 0.6 | 0.3 | 0.55 | 0.35 |
| 34422 | APL12985 | AT | F | 15.69 | 4.8 | 5.5 | 2.25 | 1.6 | 2.5 | 2.55 | 4.3 | 0.75 | 3.4 | 4 | 3 | 2.7 | 4 | 2.7 | 1.1 | 7.8 | 7 | 7.9 | 0.5 | 0.45 | 0.7 | 0.3 |
| 34424 | APL12966 | AT | F | 16.3 | 5 | 5.6 | 2.5 | 1.65 | 2.45 | 2.55 | 4.7 | 1 | 3.6 | 4.1 | 3.2 | 2.8 | 4 | 2.7 | 1.2 | 7.7 | 6.7 | 7.6 | 0.6 | 0.5 | 0.75 | 0.35 |

**Table S3.** Morphometric measurements (in mm) of tadpoles of *Allobates kamilae* sp. nov. (clade E). Abbreviations: INPAH, museum voucher catalog numbers; FN, field number; GS, Gosner stage. Measurement acronyms are described in the text.

| INPAH | FN | Sampling module | GS | TL | BL | TAL | BW | BH | HWLE | TMW | MTH | TMH | IOD | IND | END | NSD | ED | VTL | SLT | ODW |
| --- | --- | --- | --- | --- | --- | --- | --- | --- | --- | --- | --- | --- | --- | --- | --- | --- | --- | --- | --- | --- |
| 42851 | 17069 | *Jirau Direito* | 39 | 20.2 | 3.6 | 15.1 | 4.7 | 3.8 | 4.4 | 2.2 | 2.4 | 1.9 | 1.1 | 1.0 | 0.7 | 0.4 | 1.3 | 1.0 | 1.5 | 1.4 |
| 42851 | 17069 | *Jirau Direito* | 39 | 20.3 | 4.0 | 15.6 | 5.2 | 4.1 | 4.8 | 2.6 | 3.0 | 2.3 | 1.2 | 1.0 | 0.5 | 0.6 | 1.1 | 1.5 | 1.3 | 1.3 |
| 42851 | 17069 | *Jirau Direito* | 39 | 20.0 | 3.6 | 12.8 | 4.7 | 4.0 | 4.2 | 2.1 | 2.5 | 2.0 | 1.1 | 1.0 | 0.6 | 0.5 | 1.2 | 1.1 | 1.5 | 1.1 |
| 42851 | 17069 | *Jirau Direito* | 39 | 20.0 | 3.9 | 13.3 | 4.9 | 3.9 | 5.0 | 2.4 | 3.0 | 2.0 | 1.2 | 0.9 | 0.4 | 0.5 | 1.2 | 1.2 | 1.3 | 1.5 |
| 42851 | 17069 | *Jirau Direito* | 39 | 20.0 | 4.0 | 13.8 | 5.0 | 3.9 | 4.7 | 2.1 | 2.8 | 2.2 | 1.0 | 0.8 | 0.5 | 0.5 | 1.1 | 1.4 | 1.5 | 1.9 |
| 42851 | 17069 | *Jirau Direito* | 40 | 20.4 | 4.4 | 15.1 | 5.2 | 4.1 | 5.5 | 2.7 | 3.0 | 2.3 | 1.3 | 0.9 | 0.6 | 0.6 | 1.2 | 1.3 | 1.1 | 1.5 |
| 42851 | 17069 | *Jirau Direito* | 40 | 20.3 | 4.0 | 15.0 | 5.4 | 4.0 | 5.2 | 2.4 | 3.0 | 2.2 | 1.1 | 1.0 | 0.5 | 0.7 | 1.3 | 1.1 | 1.0 | 1.5 |
| 42851 | 17069 | *Jirau Direito* | 40 | 20.3 | 3.6 | 14.5 | 5.3 | 3.7 | 5.0 | 2.4 | 3.4 | 2.0 | 1.1 | 1.0 | 0.4 | 0.7 | 1.2 | 1.5 | 1.0 | 1.6 |
| 42851 | 17069 | *Jirau Direito* | 40 | 20.1 | 3.6 | 14.4 | 5.0 | 3.5 | 5.0 | 2.2 | 2.5 | 1.8 | 1.2 | 1.0 | 0.6 | 0.5 | 1.2 | 1.3 | 1.0 | 1.8 |
| 42851 | 17069 | *Jirau Direito* | 40 | 20.2 | 4.1 | 13.9 | 5.1 | 4.0 | 5.2 | 2.5 | 3.0 | 2.1 | 1.2 | 0.9 | 0.5 | 0.6 | 1.3 | 1.3 | 1.3 | 1.5 |
| 42851 | 17069 | *Jirau Direito* | 40 | 20.1 | 4.0 | 13.8 | 5.2 | 3.9 | 5.0 | 2.3 | 2.8 | 2.0 | 1.1 | 0.9 | 0.5 | 0.5 | 1.1 | 1.1 | 1.0 | 1.5 |
| 42851 | 17069 | *Jirau Direito* | 40 | 20.3 | 4.2 | 14.4 | 5.4 | 3.8 | 5.4 | 2.7 | 1.5 | 2.2 | 1.2 | 1.0 | 0.5 | 0.7 | 1.2 | 1.1 | 1.5 | 1.5 |
| 42851 | 17069 | *Jirau Direito* | 40 | 20.3 | 4.3 | 15.3 | 5.5 | 4.2 | 5.6 | 2.4 | 2.5 | 2.3 | 1.1 | 1.0 | 0.7 | 0.6 | 1.2 | 1.1 | 1.3 | 1.7 |
| 42851 | 17069 | *Jirau Direito* | 37 | 17.7 | 3.5 | 11.3 | 5.2 | 3.7 | 4.2 | 1.9 | 2.3 | 2.1 | 1.0 | 0.9 | 0.6 | 0.7 | 1.1 | 1.1 | 1.3 | 1.5 |
| 42851 | 17069 | *Jirau Direito* | 38 | 20.3 | 3.8 | 15.1 | 5.6 | 4.0 | 5.6 | 2.5 | 2.7 | 2.3 | 1.1 | 0.9 | 0.5 | 0.7 | 1.2 | 1.4 | 1.5 | 2.0 |
| 42851 | 17069 | *Jirau Direito* | 38 | 20.2 | 4.1 | 13.9 | 5.3 | 3.9 | 5.0 | 2.4 | 3.2 | 2.2 | 1.2 | 1.0 | 0.5 | 0.7 | 1.2 | 1.0 | 1.3 | 1.5 |
| 42851 | 17069 | *Jirau Direito* | 38 | 20.1 | 3.6 | 14.0 | 5.0 | 3.5 | 4.7 | 2.2 | 3.0 | 1.8 | 1.2 | 0.9 | 0.6 | 0.4 | 1.1 | 1.4 | 1.5 | 1.6 |
| 42851 | 17069 | *Jirau Direito* | 38 | 20.2 | 4.0 | 14.5 | 4.9 | 3.2 | 5.1 | 2.4 | 2.3 | 2.1 | 1.1 | 0.9 | 0.5 | 0.4 | 1.2 | 1.1 | 1.5 | 1.5 |
| 42851 | 17069 | *Jirau Direito* | 38 | 20.2 | 3.8 | 15.0 | 5.0 | 3.5 | 5.5 | 2.0 | 3.0 | 1.9 | 1.1 | 0.8 | 0.6 | 0.6 | 1.2 | 0.9 | 1.3 | 1.3 |
| 42852 | 17070 | *Morrinhos* | 38 | 20.3 | 3.5 | 15.5 | 4.7 | 3.7 | 5.0 | 2.4 | 3.0 | 1.8 | 1.2 | 0.9 | 0.5 | 0.5 | 1.3 | 0.8 | 1.3 | 1.6 |
| 42852 | 17070 | *Morrinhos* | 39 | 20.3 | 3.7 | 15.4 | 4.7 | 3.7 | 4.7 | 2.5 | 3.2 | 2.1 | 1.2 | 1.0 | 0.5 | 0.5 | 1.2 | 1.0 | 1.5 | 2.0 |
| 42852 | 17070 | *Morrinhos* | 39 | 20.3 | 3.6 | 15.1 | 4.8 | 3.7 | 4.9 | 2.4 | 3.1 | 2.1 | 1.0 | 1.1 | 0.7 | 0.5 | 1.3 | 1.5 | 1.0 | 2.0 |
| 42852 | 17070 | *Morrinhos* | 39 | 20.3 | 3.5 | 15.2 | 4.4 | 3.5 | 4.5 | 2.2 | 3.0 | 1.9 | 1.2 | 1.1 | 0.6 | 0.3 | 1.2 | 1.0 | 1.0 | 1.6 |
| 42852 | 17070 | *Morrinhos* | 39 | 20.3 | 3.8 | 15.1 | 5.1 | 3.7 | 4.8 | 2.3 | 2.8 | 1.9 | 1.1 | 1.1 | 0.6 | 0.6 | 1.4 | 1.1 | 1.0 | 2.0 |
| 42852 | 17070 | *Morrinhos* | 39 | 20.2 | 3.7 | 15.0 | 4.9 | 3.5 | 4.6 | 2.2 | 2.8 | 2.0 | 1.1 | 0.8 | 0.5 | 0.7 | 1.2 | 1.1 | 1.4 | 1.8 |
| 42852 | 17070 | *Morrinhos* | 39 | 20.4 | 3.7 | 15.1 | 4.7 | 3.7 | 4.9 | 2.4 | 3.1 | 2.0 | 1.2 | 1.0 | 0.5 | 0.4 | 1.3 | 1.1 | 1.2 | 1.8 |
| 42852 | 17070 | *Morrinhos* | 39 | 20.3 | 3.7 | 15.1 | 4.8 | 3.8 | 5.0 | 2.4 | 3.2 | 2.0 | 1.3 | 0.9 | 0.3 | 0.7 | 1.1 | 1.1 | 1.5 | 1.8 |
| 42852 | 17070 | *Morrinhos* | 40 | 20.3 | 3.8 | 15.1 | 4.7 | 4.0 | 5.0 | 2.3 | 3.1 | 1.9 | 1.1 | 0.9 | 0.7 | 0.4 | 1.3 | 1.0 | 1.5 | 1.8 |
| 42852 | 17070 | *Morrinhos* | 40 | 20.2 | 3.6 | 15.0 | 4.5 | 3.7 | 4.1 | 2.2 | 2.7 | 2.0 | 1.1 | 0.9 | 0.7 | 0.4 | 1.3 | 1.0 | 1.1 | 1.5 |
| 42852 | 17070 | *Morrinhos* | 40 | 20.3 | 3.7 | 15.8 | 4.4 | 3.7 | 4.7 | 2.3 | 3.1 | 2.1 | 1.2 | 1.1 | 0.7 | 0.4 | 1.4 | 1.2 | 1.1 | 2.0 |
| 42852 | 17070 | *Morrinhos* | 40 | 20.5 | 4.0 | 15.1 | 5.2 | 3.9 | 5.2 | 2.6 | 3.4 | 1.8 | 1.1 | 1.1 | 0.7 | 0.4 | 1.3 | 0.8 | 1.4 | 1.8 |
| 42852 | 17070 | *Morrinhos* | 40 | 20.3 | 3.7 | 15.1 | 4.8 | 2.8 | 4.8 | 2.4 | 3.2 | 2.3 | 1.2 | 1.1 | 0.7 | 0.5 | 1.3 | 1.5 | 1.2 | 1.8 |
| 42852 | 17070 | *Morrinhos* | 40 | 20.2 | 3.6 | 15.1 | 4.3 | 3.8 | 4.5 | 2.2 | 3.1 | 1.9 | 1.2 | 1.0 | 0.5 | 0.5 | 1.3 | 0.9 | 1.4 | 1.7 |
| 42853 | 17071 | *Jirau Direito* | 38 | 20.2 | 2.8 | 13.8 | 4.6 | 3.0 | 4.2 | 2.0 | 2.5 | 2.0 | 1.0 | 0.8 | 0.4 | 0.5 | 1.1 | 1.2 | 1.0 | 1.2 |
| 42853 | 17071 | *Jirau Direito* | 38 | 21.2 | 2.0 | 15.1 | 4.6 | 3.4 | 4.3 | 2.0 | 3.3 | 1.9 | 1.0 | 0.8 | 0.4 | 0.4 | 1.2 | 1.3 | 1.5 | 1.5 |
| 42853 | 17071 | *Jirau Direito* | 38 | 19.8 | 3.0 | 12.5 | 4.9 | 2.9 | 4.1 | 2.0 | 2.5 | 2.0 | 1.1 | 0.9 | 0.5 | 0.4 | 1.2 | 1.3 | 1.6 | 1.1 |
| 42853 | 17071 | *Jirau Direito* | 34 | 20.0 | 2.7 | 13.6 | 4.8 | 3.5 | 4.1 | 2.0 | 3.0 | 1.9 | 1.2 | 1.0 | 0.4 | 0.4 | 1.1 | 1.2 | 1.8 | 1.1 |
| 42853 | 17071 | *Jirau Direito* | 35 | 18.2 | 3.9 | 12.3 | 4.3 | 3.3 | 3.2 | 2.1 | 2.2 | 1.9 | 0.9 | 0.9 | 0.5 | 0.4 | 1.0 | 1.0 | 1.5 | 1.5 |
| 42853 | 17071 | *Jirau Direito* | 36 | 16.7 | 1.7 | 11.3 | 3.9 | 2.7 | 3.0 | 1.6 | 2.3 | 1.7 | 1.0 | 0.8 | 0.5 | 0.3 | 1.0 | 1.3 | 1.5 | 1.3 |
| 42853 | 17071 | *Jirau Direito* | 36 | 19.7 | 2.0 | 13.1 | 5.0 | 2.3 | 4.5 | 2.0 | 3.0 | 2.0 | 1.1 | 0.9 | 0.4 | 0.4 | 1.0 | 1.4 | 1.3 | 1.5 |
| 42853 | 17071 | *Jirau Direito* | 36 | 19.7 | 3.5 | 13.1 | 4.6 | 3.1 | 4.0 | 2.3 | 2.7 | 2.0 | 1.1 | 0.8 | 0.3 | 0.5 | 1.2 | 1.2 | 1.3 | 1.5 |
| 42853 | 17071 | *Jirau Direito* | 36 | 18.7 | 3.0 | 12.3 | 4.8 | 3.2 | 4.1 | 2.0 | 2.7 | 1.9 | 1.1 | 0.9 | 0.4 | 0.3 | 1.1 | 1.1 | 1.5 | 1.5 |
| 42853 | 17071 | *Jirau Direito* | 37 | 20.0 | 2.6 | 13.9 | 4.7 | 3.6 | 4.2 | 2.0 | 3.0 | 1.8 | 1.0 | 0.9 | 0.6 | 0.3 | 1.0 | 1.3 | 1.3 | 1.5 |
| 42853 | 17071 | *Jirau Direito* | 37 | 20.0 | 2.1 | 14.4 | 5.0 | 3.4 | 4.5 | 2.2 | 2.8 | 2.0 | 1.0 | 0.9 | 0.3 | 0.3 | 1.1 | 1.0 | 1.2 | 1.5 |
| 42853 | 17071 | *Jirau Direito* | 37 | 20.3 | 2.0 | 13.6 | 5.1 | 3.4 | 4.0 | 2.1 | 2.4 | 1.9 | 1.1 | 0.9 | 0.4 | 0.4 | 1.2 | 1.3 | 1.3 | 1.1 |
| 42853 | 17071 | *Jirau Direito* | 37 | 19.3 | 2.1 | 13.1 | 4.3 | 3.2 | 3.0 | 2.0 | 2.6 | 2.0 | 1.0 | 0.9 | 0.3 | 0.3 | 1.2 | 1.0 | 1.5 | 1.2 |
| 42853 | 17071 | *Jirau Direito* | 37 | 20.0 | 2.0 | 13.9 | 4.8 | 3.7 | 4.1 | 2.0 | 2.9 | 2.2 | 1.1 | 0.9 | 0.4 | 0.5 | 1.2 | 1.0 | 1.0 | 1.2 |
| 42853 | 17071 | *Jirau Direito* | 37 | 16.0 | 2.4 | 9.4 | 5.5 | 3.6 | 4.8 | 2.0 | 2.8 | 2.0 | 1.2 | 1.1 | 0.5 | 0.7 | 1.3 | 1.4 | 1.4 | 1.5 |
| 42853 | 17071 | *Jirau Direito* | 37 | 20.5 | 2.0 | 14.4 | 4.5 | 3.2 | 3.5 | 2.0 | 2.4 | 2.2 | 1.1 | 0.9 | 0.3 | 0.3 | 1.3 | 0.9 | 1.0 | 1.0 |
| 42853 | 17071 | *Jirau Direito* | 37 | 19.3 | 2.0 | 12.5 | 4.9 | 2.3 | 4.3 | 2.0 | 2.7 | 2.0 | 1.0 | 0.8 | 0.3 | 0.5 | 1.3 | 1.0 | 1.7 | 1.7 |
| 42853 | 17071 | *Jirau Direito* | 37 | 20.0 | 3.0 | 13.8 | 5.0 | 3.2 | 4.1 | 2.0 | 3.0 | 1.9 | 1.2 | 1.1 | 0.6 | 0.5 | 1.2 | 1.5 | 1.8 | 1.2 |

**Table S4.** Acoustic parameters of the two-note calls of *Allobates kamilae* sp. nov. (clade E) and *A. tapajos* sensu stricto (clade A). Abbreviations: SP, species; AK, *A. kamilae* sp. nov.; AT, *A. tapajos*; INPAH, museum voucher catalog numbers; FNJV, call voucher recordings; AT, air temperature (ºC); CD, call duration (ms); ND, note duration (ms), INI, inter-note interval (ms); ICI, inter-call interval (ms); DF, dominant frequency (Hz).

| SP | INPAH | FNJV | AT | CD | ND | INI | ICI | DF |
| --- | --- | --- | --- | --- | --- | --- | --- | --- |
| AT | 34415 | 50556 | 24.4 | 199.0 | 31.0 | 135.8 | 388.2 | 5951.6 |
| AT | 34410 | 50557 | 24.0 | 215.6 | 39.8 | 134.2 | 354.2 | 5934.4 |
| AT | 34405 | 50558 | 24.0 | 233.6 | 40.2 | 152.8 | 608.8 | 5779.0 |
| AT | 34414 | 50559 | 24.0 | 205.8 | 35.4 | 134.2 | 418.2 | 5434.6 |
| AT | 34425 | 50560 | 23.8 | 220.0 | 39.6 | 141.6 | 409.8 | 5529.0 |
| AT | 34416 | 50561 | 23.9 | 222.6 | 41.0 | 136.6 | 458.0 | 5477.6 |
| AT | 34402 | 50562 | 23.9 | 181.0 | 31.6 | 116.2 | 457.6 | 5314.2 |
| AK | 42882 | 50573 | 25 | 396.00 | 35.33 | 138.90 | 699.80 | 5666.80 |
| AK | 42956 | 50574 | 24.5 | 409.00 | 29.80 | 159.80 | 872.80 | 5297.00 |
| AK | 42881 | 50575 | 25.1 | 360.20 | 32.93 | 130.40 | 952.80 | 5340.00 |
| AK | 42944 | 50576 | 26.1 | 358.40 | 34.40 | 127.80 | 888.80 | 5830.80 |
| AK | Unvouchered1 | 50569 | 23.8 | 372.00 | 30.73 | 139.50 | 676.00 | 5779.40 |
| AK | Unvouchered2 | 50570 | 24.5 | 391.67 | 30.67 | 149.50 | 1346.50 | 5569.33 |
| AK | Unvouchered3 | 50571 | 24.8 | 372.20 | 31.60 | 138.50 | 1163.00 | 5813.60 |
| AK | Unvouchered4 | 50572 | 24.5 | 376.00 | 38.17 | 130.75 | 1402.50 | 5641.00 |
| AK | 42902 | 50583 | 25.3 | 382.60 | 31.80 | 143.70 | 596.00 | 5770.00 |
| AK | 42937 | 50584 | 25.5 | 418.20 | 30.93 | 162.20 | 700.20 | 5727.00 |
| AK | 42952 | 50581 | 25.3 | 400.80 | 34.00 | 149.70 | 422.40 | 5934.40 |
| AK | 42923 | 50582 | 25.7 | 396.20 | 31.60 | 149.90 | 535.40 | 5770.00 |
| AK | Unvouchered5 | 50580 | 25.9 | 368.20 | 27.87 | 142.20 | 724.40 | 5546.40 |
| AK | 42965 | 50592 | 25.7 | 317.20 | 28.87 | 115.50 | 627.20 | 5563.60 |
| AK | 42901 | 50593 | 25.3 | 377.40 | 34.53 | 137.00 | 561.80 | 5537.80 |
| AK | Unvouchered6 | 50585 | 28 | 300.60 | 24.20 | 114.00 | 564.20 | 6132.20 |
| AK | 42911 | 50577 | 27.1 | 361.40 | 27.33 | 139.50 | 632.40 | 5951.60 |
| AK | 42900 | 50578 | 24.3 | 433.60 | 41.53 | 154.50 | 560.40 | 5933.60 |
| AK | 42932 | 50579 | 24 | 497.71 | 43.33 | 183.64 | 676.57 | 6072.00 |
| AK | 42935 | 50564 | 28.6 | 280.40 | 19.67 | 110.20 | 537.60 | 5537.80 |
| AK | 42951 | 50565 | 27.4 | 297.00 | 23.00 | 114.50 | 425.00 | 5512.00 |
| AK | 42897 | 50567 | 25 | 409.60 | 33.80 | 153.30 | 555.80 | 6115.00 |
| AK | 42904 | 50568 | 25 | 393.40 | 32.07 | 148.60 | 515.60 | 6089.20 |
| AK | 42878 | 50566 | 25 | 429.00 | 36.56 | 159.67 | 457.33 | 6129.33 |
| AK | 42909 | 50590 | 26.2 | 332.20 | 33.93 | 115.20 | 700.80 | 5451.80 |
| AK | 42921 | 50591 | 26.2 | 314.80 | 29.73 | 113.20 | 452.60 | 5331.40 |
| AK | 42960 | 50586 | 25 | 370.00 | 32.6 | 136.3 | 873.00 | 5650.00 |
| AK | 42883 | 50587 | 25 | 386.00 | 33.8 | 141.7 | 819.00 | 5306.00 |
| AK | 42898 | 50588 | 25 | 371.00 | 30.67 | 139.00 | 556.00 | 5727.00 |
| AK | 42891 | 50589 | 25 | 388.00 | 30.60 | 149.10 | 852.00 | 5435.00 |

**Table S5.** Acoustic parameters of the three-note call of *Allobates kamilae* sp. nov. (clade E) from the west and east banks of the upper Madeira River. Abbreviations: INPAH, museum voucher catalog numbers; FNJV, call voucher recordings; AT, air temperature (ºC); CD, call duration (ms); ND, note duration (ms), INI, inter-note interval (ms); ICI, inter-call interval (ms); DF, dominant frequency (Hz).

| INPAH | FNJV | AT | Sampling site | Riverbank | CD | ND | INI | ICI | DF |
| --- | --- | --- | --- | --- | --- | --- | --- | --- | --- |
| 42882 | 50573 | 25 | Abunã Esquerdo | West | 396.00 | 35.33 | 138.90 | 699.80 | 5,666.80 |
| 42956 | 50574 | 24.5 | Abunã Esquerdo | West | 409.00 | 29.80 | 159.80 | 872.80 | 5,297.00 |
| 42881 | 50575 | 25.1 | Abunã Esquerdo | West | 360.20 | 32.93 | 130.40 | 952.80 | 5,340.00 |
| 42944 | 50576 | 26.1 | Abunã Esquerdo | West | 358.40 | 34.40 | 127.80 | 888.80 | 5,830.80 |
| Unvouchered | 50569 | 23.8 | Abunã Esquerdo | West | 372.00 | 30.73 | 139.50 | 676.00 | 5,779.40 |
| Unvouchered | 50570 | 24.5 | Abunã Esquerdo | West | 391.67 | 30.67 | 149.50 | 1,346.50 | 5,569.33 |
| Unvouchered | 50571 | 24.8 | Abunã Esquerdo | West | 372.20 | 31.60 | 138.50 | 1,163.00 | 5,813.60 |
| Unvouchered | 50572 | 24.5 | Abunã Esquerdo | West | 376.00 | 38.17 | 130.75 | 1,402.50 | 5,641.00 |
| 42902 | 50583 | 25.3 | Búfalos | West | 382.60 | 31.80 | 143.70 | 596.00 | 5,770.00 |
| 42937 | 50584 | 25.5 | Búfalos | West | 418.20 | 30.93 | 162.20 | 700.20 | 5,727.00 |
| 42952 | 50581 | 25.3 | Búfalos | West | 400.80 | 34.00 | 149.70 | 422.40 | 5,934.40 |
| 42923 | 50582 | 25.7 | Búfalos | West | 396.20 | 31.60 | 149.90 | 535.40 | 5,770.00 |
| Unvouchered | 50580 | 25.9 | Búfalos | West | 368.20 | 27.87 | 142.20 | 724.40 | 5,546.40 |
| 42965 | 50592 | 25.7 | Jirau Esquerdo | West | 317.20 | 28.87 | 115.50 | 627.20 | 5,563.60 |
| 42901 | 50593 | 25.3 | Jirau Esquerdo | West | 377.40 | 34.53 | 137.00 | 561.80 | 5,537.80 |
| Unvouchered | 50585 | 28 | Pedras | West | 300.60 | 24.20 | 114.00 | 564.20 | 6,132.20 |
| 42911 | 50577 | 27.1 | Abunã Direito | East | 361.40 | 27.33 | 139.50 | 632.40 | 5,951.60 |
| 42900 | 50578 | 24.3 | Abunã Direito | East | 433.60 | 41.53 | 154.50 | 560.40 | 5,933.60 |
| 42932 | 50579 | 24 | Abunã Direito | East | 497.71 | 43.33 | 183.64 | 676.57 | 6,072.00 |
| 42935 | 50564 | 28.6 | Jirau Direito | East | 280.40 | 19.67 | 110.20 | 537.60 | 5,537.80 |
| 42951 | 50565 | 27.4 | Jirau Direito | East | 297.00 | 23.00 | 114.50 | 425.00 | 5,512.00 |
| 42897 | 50567 | 25 | Jirau Direito | East | 409.60 | 33.80 | 153.30 | 555.80 | 6,115.00 |
| 42904 | 50568 | 25 | Jirau Direito | East | 393.40 | 32.07 | 148.60 | 515.60 | 6,089.20 |
| 42878 | 50566 | 25 | Jirau Direito | East | 429.00 | 36.56 | 159.67 | 457.33 | 6,129.33 |
| 42909 | 50590 | 26.2 | Morrinhos | East | 332.20 | 33.93 | 115.20 | 700.80 | 5,451.80 |
| 42921 | 50591 | 26.2 | Morrinhos | East | 314.80 | 29.73 | 113.20 | 452.60 | 5,331.40 |
| 42960 | 50586 | 25 | Morrinhos | East | 370.00 | 32.6 | 136.3 | 873.00 | 5,650.00 |
| 42883 | 50587 | 25 | Morrinhos | East | 386.00 | 33.8 | 141.7 | 819.00 | 5,306.00 |
| 42898 | 50588 | 25 | Morrinhos | East | 371.00 | 30.67 | 139.00 | 556.00 | 5,727.00 |
| 42891 | 50589 | 25 | Morrinhos | East | 388.00 | 30.60 | 149.10 | 852.00 | 5,435.00 |

**Table S6**. Loadings of and variance explained by the first two shape principal components (PC) based on 23 morphometric measurements of *Allobates* *kamilae* sp. nov. and *A. tapajos* (Interspecific Shape PCAs) and of *A*. *kamilae* sp. nov. from opposite banks of the upper Madeira River (Intraspecific Shape PCAs). Trait acronyms are defined in the text. Abbreviations: n, sample size.

| Traits | Interspecific Shape PCA | | | | Intraspecific Shape PCA | | | |
| --- | --- | --- | --- | --- | --- | --- | --- | --- |
|  | Females (n = 34) | | Males (n = 63) | | Females (n = 24) | | Males (n = 49) | |
|  | shape.PC1 | shape.PC2 | shape.PC1 | shape.PC2 | shape.PC1 | shape.PC2 | shape.PC1 | shape.PC2 |
| DPT | 0.058 | 0.067 | 0.081 | -0.047 | 0.135 | **0.552** | 0.059 | -0.044 |
| EL | -0.052 | 0.052 | 0.036 | -0.107 | 0.004 | 0.016 | 0.112 | -0.056 |
| EN | 0.135 | -0.061 | **-0.194** | -0.114 | 0.008 | 0.054 | -0.018 | -0.036 |
| FAL | 0.018 | 0.035 | -0.030 | 0.047 | 0.094 | **0.190** | -0.066 | -0.041 |
| FL | 0.075 | 0.035 | -0.020 | -0.035 | 0.045 | -0.067 | 0.013 | 0.040 |
| HANDI | -0.053 | 0.138 | 0.055 | -0.105 | 0.101 | -0.164 | 0.106 | -0.108 |
| HANDII | 0.064 | 0.172 | -0.058 | -0.113 | **0.168** | -0.087 | 0.071 | -0.073 |
| HANDIII | 0.079 | 0.105 | -0.083 | -0.058 | 0.105 | -0.018 | 0.003 | -0.112 |
| HANDIV | 0.034 | **0.177** | -0.013 | -0.072 | **0.163** | -0.108 | 0.075 | -0.157 |
| HL | **0.255** | -0.001 | **-0.252** | -0.045 | 0.117 | 0.133 | -0.108 | 0.168 |
| HW | **0.177** | 0.000 | -0.124 | -0.032 | 0.075 | 0.101 | -0.046 | 0.131 |
| IN | 0.044 | 0.011 | -0.054 | -0.001 | 0.027 | 0.050 | -0.036 | 0.036 |
| IO | 0.079 | 0.001 | -0.083 | 0.001 | 0.039 | -0.013 | -0.043 | 0.113 |
| SL | 0.024 | -0.025 | -0.143 | -0.157 | -0.025 | -0.151 | 0.079 | -0.094 |
| SVL | 0.133 | -0.037 | -0.130 | -0.011 | 0.027 | 0.079 | -0.068 | 0.067 |
| THL | 0.104 | 0.021 | -0.120 | 0.026 | 0.067 | 0.102 | -0.094 | 0.054 |
| TL | 0.076 | -0.012 | -0.077 | 0.008 | 0.032 | 0.159 | -0.041 | -0.013 |
| TYM | -0.076 | **-0.804** | 0.061 | **0.260** | **-0.777** | 0.071 | **-0.169** | **-0.401** |
| UAL | -0.070 | 0.085 | 0.124 | -0.116 | 0.032 | -0.017 | **0.185** | -0.089 |
| WFD | **-0.787** | **0.293** | **0.650** | **0.360** | -0.051 | **-0.436** | 0.048 | **-0.281** |
| WPF | 0.060 | 0.008 | -0.101 | **0.740** | 0.055 | 0.076 | **-0.711** | **0.441** |
| WTD | 0.046 | 0.116 | -0.101 | -0.046 | 0.062 | **-0.555** | 0.062 | -0.167 |
| WTT | **-0.425** | **-0.375** | **0.576** | **-0.384** | **-0.503** | 0.035 | **0.587** | **0.624** |
| Variance (%) | 55.0 | 12.4 | 43.0 | 16.4 | 30.2 | 14.5 | 31.6 | 14.4 |

**Table S7.** Loadings of and variance explained by the first two principal components based on 5 acoustic parameters of *Allobates* *kamilae* sp. nov. and *A. tapajos* (Interspecific PCA) and on 4 acoustic parameters of *A*. *kamilae* sp. nov. from opposite banks of the upper Madeira River (Intraspecific PCA). Trait acronyms are defined in the text. Abbreviations: n, sample size; PC, principal component; PCAs, principal component analyses.

| Traits | Interspecific PCA (n = 37) | | Intraspecific PCA (n = 30) | |
| --- | --- | --- | --- | --- |
|  | PC1 | PC2 | PC1 | PC2 |
| Call duration | **0.527** | -0.391 | – | – |
| Note duration | 0.379 | 0.340 | **-0.608** | 0.266 |
| Inter-note interval | **0.623** | 0.091 | **-0.641** | 0.097 |
| Inter-call interval | 0.187 | **-0.746** | -0.028 | **0.803** |
| Dominant frequency | 0.393 | **0.408** | -0.468 | **-0.524** |
| Variance (%) | 41.7 | 26.4 | 46.3 | 32.1 |

**Table S8.** Advertisement call parameters of *Allobates kamilae* sp. nov. Values depict mean ± standard error (range). Abbreviations: n, number of calls analyzed; Hz, Hertz.

| Call arrangement | Call duration (ms) | Note duration (ms) | Inter-note interval (ms) | Inter-call interval (ms) | Dominant frequency (Hz) |
| --- | --- | --- | --- | --- | --- |
| 2 notes (n = 100) | 211 ± 35 (141–333) | 31 ± 6 (19–47) | 148 ± 29 (85–241) | 692 ± 299 (302–2,077) | 5,744 ± 274 (5,211–6,201) |
| 3 notes (n = 125) | 376 ± 60 (260–593) | 32 ± 6 (18–49) | 140 ± 27 (96–247) | 673 ± 299 (309–2,236) | 5,736 ± 267 (5,211–6,201) |
| 4 notes (n = 118) | 527 ± 73 (387–794) | 32 ± 5 (19–49) | 136 ± 28 (94–268) | 678 ± 238 (299–1,778) | 5,719 ± 248 (5,211–6,201) |
| 5 notes (n = 60) | 699 ± 93 (542–1,120) | 32 ± 5 (15–48) | 136 ± 28 (96–258) | 810 ± 610 (393–4,743) | 5,636 ± 225 (5,254–6,115) |
| 6 notes (n = 22) | 890 ± 145 (704–1,327) | 32 ± 5 (16–39) | 142 ± 28 (98–216) | 1,023 ± 766 (391–3,893) | 5,590 ± 174 (5,383–5,857) |
| 7 notes (n = 12) | 1,075 ± 196 (894–1,514) | 31 ± 6 (14–38) | 144 ± 37 (107–225) | 2,328 ± 3,157 (552–9,926) | 5,717 ± 203 (5,426–6,115) |
| 8 notes (n = 11) | 1,128 ± 82 (1,028–1,266) | 32 ± 6 (16–40) | 127 ± 16 (102–156) | 984 ± 350 (631–1,801) | 5,731 ± 157 (5,469–5,986) |
| 9 notes (n = 13) | 1,287 ± 144 (1,144–1,584) | 32 ± 5 (21–40) | 131 ± 25 (95–191) | 1,253 ± 1,698 (447–6,607) | 5,775 ± 110 (5,426–5,770) |
| 10 notes (n = 4) | 1,507 ± 233 (1,288–1,744) | 27 ± 4 (20–33) | 135 ± 27 (94–173) | 9,790 ± 14,159 (703–30,521) | 5,512 ± 50 (5,469–5,555) |
| 11 notes (n = 4) | 1,865 ± 241 (1,504–1,995) | 27 ± 2 (22–31) | 156 ± 26 (115–183) | 1,1593 ± 7,234 (1,099–17,678) | 5,405 ± 129 (5,297–5,555) |
| 12 notes (n = 5) | 2,126 ± 125 (1,952–2,284) | 28 ± 2 (24–33) | 171 ± 17 (141–198) | 5,165 ± 4,304 (748–10,474) | 5,495 ± 135 (5,254–5,555) |
| 14 notes (n = 1) | 2,308 | 25 ± 4 (21–28) | 160 ± 4 (157–163) | 946 | 5,555 |
| 17 notes (n = 1) | 3,062 | 23 ± 7 (17–30) | 165 ± 5 (161–168) | 9,616 | 5,555 |


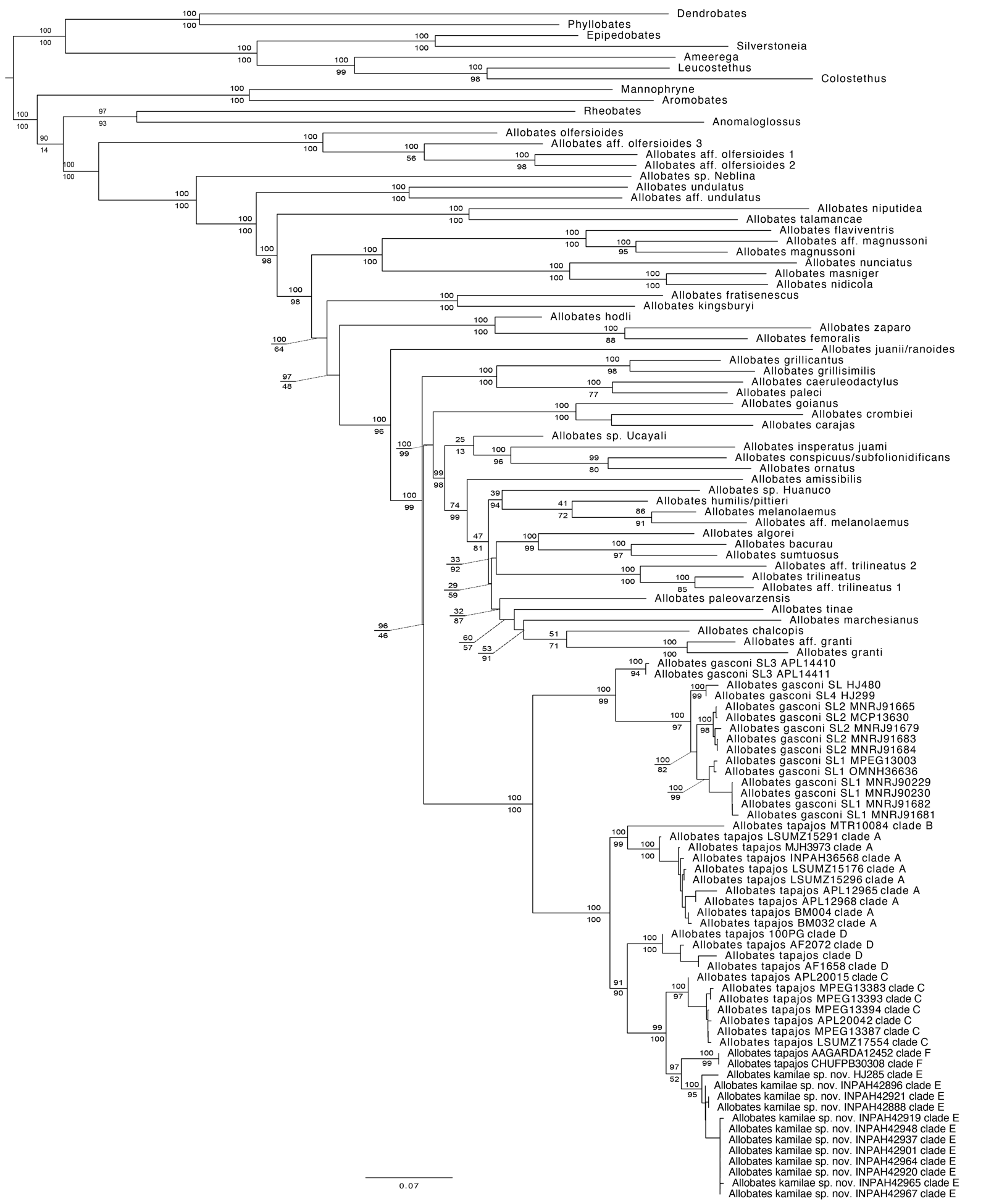


**Figure S1.** Maximum Likelihood phylogenetic tree based on five mitochondrial genes (12S, 16S, COI, ND1 and CYTB) of *Allobates*. Bootstrap and SH-aLRT support of major clades is shown above and below nodes, respectively.

**Script S1.** Script of Shape PCA and LDA Ratio Extractor (Baur and Leuenberger 2011) adapted from Baur et al. (2020). The script is exemplified with morphometric data from males of *Allobates tapajos* Clade A and Clade E. Change the file name to run the script with another dataset.

## source (https://doi.org/10.5281/zenodo.4250142) and data files must be in the same folder

rm(list=ls()); ls() # Tidying up workspace

library(ggplot2)

library(plyr)

source("Shape_PCA_v1.02.R", chdir = TRUE) # Load the script

#### Example using males of *Allobates tapajos* and A. sp. nov. ####

#### Load dataset

filename <- "Males_interespecific.csv"

dat0 <- read.csv(filename, sep = ";")

# Using all data

dat <- dat0

names(dat)

X <- dat[,5:27] # Selection of numeric variables

X <- X[, order(names(X))] # Ordering variables alphabetically

species <- factor(dat$SP) # Set groups as factor, here species

no <- dat$INPAH # Individual code number for specimens, here museum vouchers

############## Shape PCA

# Calculating Shape PCA

SPCA <- ShapePCA(U=X,

npc=4, # Number of shapePCs retained, default setting

rpc=1 # Rounding of variance explained by shapePCs, default setting

)

SPCA # Printing output

## Plotting the variance explained by the first 4 shapePCs

barplot(SPCA$pc_var, main="Screeplot", xlab="shapePC", ylab="% variance explained")

# Extracting the isosize, shapePC1 and shapePC2 to be plotted

isosize <- as.numeric(SPCA$isosize)

shapePC1 <- SPCA$PCmatrix[,1]

shapePC2 <- SPCA$PCmatrix[,2]

############## ANOVAs

anova_isosize<- aov(isosize~species) ## ANOVA isosize

summary.aov(anova_isosize)

anova_shapePC1<- aov(shapePC1~species) ## ANOVA shape PC1

summary.aov(anova_shapePC1)

anova_shapePC2<- aov(shapePC2~species) ## ANOVA shape PC2

summary.aov(anova_shapePC2)

## Plotting shapePC1, shapePC2 and isosize

# Selecting colors for symbols in scatterplot

colors <- c("AK"="#6b4f9b", "AT"="#f28133")

# Selecting shape of symbols

shapes <- c("AK"=15, "AT"=16)

# Specifying how the group names should appear in the legend

labels <- c("A. sp.nov.", "A. tapajos")

# Specifying the sequence of those names

breaks <- c("A. sp.nov.", "A. tapajos")

# Grouping variables together in a data frame

df <- data.frame(isosize, shapePC1, shapePC2, species, no)

############## Plotting shapePC1 against shapePC2

plot <- ggplot(df, aes(shapePC1, shapePC2, shape = species, color = species)) # Main ggplot2 function

find_hull <- function(df)df[chull(df$shapePC1, df$shapePC2),] # Function used for finding the points for drawing the convex hulls

hulls <- ddply(df, "species", find_hull) # Apply the above function by using the grouping variable 'species'

plot <- plot + geom_polygon(data = hulls, aes(group=species), colour="gray80", fill = NA) # Draw convex hulls around species

plot <- plot + geom_point(size=4) # Adjust the size of points

plot <- plot + theme(aspect.ratio=1) # Determine the aspect ratio of plot

plot <- plot + xlab(paste("shape PC1 (",SPCA$pc_var[1],"%)", sep="")) # Label for x-axis; note, the percentage of variance explained by shapePC1 is automatically inserted from the output of the ShapePCA function

plot <- plot + ylab(paste("shape PC2 (",SPCA$pc_var[2],"%)", sep="")) # Label for y-axis

plot <- plot + scale_shape_manual(values=shapes, labels=labels, breaks=breaks) # Applying values and sequence of symbol shape for the plot and its legend

plot <- plot + scale_colour_manual(values=colors, labels=labels, breaks=breaks) # Applying values and sequence of symbol colours for the plot and its legend

plot <- plot + labs(colour = "species", shape= "species") # Title of legend

plot_A <- plot + theme(axis.title.x = element_text(size=14), axis.title.y = element_text(size=14),

plot.title = element_text(size=14), legend.text = element_text(face="italic", size=13))

plot_A

############## Plotting isosize against shapePC1

plot <- ggplot(df, aes(isosize, shapePC1, shape = species, color = species))

find_hull <- function(df)df[chull(df$isosize, df$shapePC1),]

hulls <- ddply(df, "species", find_hull)

plot <- plot + geom_polygon(data = hulls, aes(group=species), colour="gray80", fill = NA)

plot <- plot + geom_point(size=4) + theme(aspect.ratio=1)

plot <- plot + xlab("isometric size")

plot <- plot + ylab(paste("shape PC1 (",SPCA$pc_var[1],"%)", sep=""))

plot <- plot + scale_shape_manual(values=shapes, labels=labels, breaks=breaks)

plot <- plot + scale_colour_manual(values=colors, labels=labels, breaks=breaks)

plot <- plot + labs(colour = "species", shape= "species")

plot_B <- plot + theme(axis.title.x = element_text(size=14), axis.title.y = element_text(size=14), plot.title = element_text(size=14), legend.text = element_text(face="italic", size=13))

plot_B

############## LDA Ratio Extractor

source("LDA_Ratio_Extractor_v1.03.R", chdir = TRUE) # Load the script

## calculating LDA ratio extractor

RE <- ldaRE(U=X, g=species,

k.max=2,

rx=1, ry=2, # Default setting

g1=1, g2=2, # Default setting

col=c(1:nlevels(species))[species], # Default setting

pch=c(1:nlevels(species))[species], # Default setting

placelegend="topright", # Default setting

cexlegend=0.9 # Default setting

)
